# Cytotoxic Compounds from Alcyoniidae: An Overview of the Last 30 Years

**DOI:** 10.3390/md20020134

**Published:** 2022-02-11

**Authors:** Federico Cerri, Francesco Saliu, Davide Maggioni, Simone Montano, Davide Seveso, Silvia Lavorano, Luca Zoia, Fabio Gosetti, Marina Lasagni, Marco Orlandi, Orazio Taglialatela-Scafati, Paolo Galli

**Affiliations:** 1 Department of Biotechnology and Biosciences, University of Milano Bicocca, Piazza della Scienza 2, 20126 Milano, Italy; federico.cerri@unimib.it; 2 Department of Earth and Environmental Sciences DISAT, University of Milano Bicocca, Piazza della Scienza 1, 20126 Milano, Italy; davide.maggioni@unimib.it (D.M.); simone.montano@unimib.it (S.M.); davide.seveso@unimib.it (D.S.); luca.zoia@unimib.it (L.Z.); fabio.gosetti@unimib.it (F.G.); marina.lasagni@unimib.it (M.L.); marco.orlandi@unimib.it (M.O.); paolo.galli@unimib.it (P.G.); 3 MaRHE Centre (Marine Research and High Education Center), Magoodhoo Island, Faafu Atoll 12030, Maldives; 4 Costa Edutainment SpA—Acquario di Genova, Area Porto Antico, Ponte Spinola, 16128 Genoa, Italy; slavorano@costaedutainment.it; 5 Department of Pharmacy, University of Naples Federico II, Via D. Montesano 49, 80131 Napoli, Italy; scatagli@unina.it

**Keywords:** coral reefs, biodiversity, bioprospecting, marine drugs, cytotoxicity, Alcyoniidae

## Abstract

The octocoral family Alcyoniidae represents a rich source of bioactive substances with intriguing and unique structural features. This review aims to provide an updated overview of the compounds isolated from Alcyoniidae and displaying potential cytotoxic activity. In order to allow a better comparison among the bioactive compounds, we focused on molecules evaluated in vitro by using the 3-(4,5-dimethylthiazol-2-yl)-2,5-diphenyl-2H-tetrazolium bromide (MTT) assay, by far the most widely used method to analyze cell proliferation and viability. Specifically, we surveyed the last thirty years of research, finding 153 papers reporting on 344 compounds with proven cytotoxicity. The data were organized in tables to provide a ranking of the most active compounds, to be exploited for the selection of the most promising candidates for further screening and pre-clinical evaluation as anti-cancer agents. Specifically, we found that (22*S*,24*S*)-24-methyl-22,25-epoxyfurost-5-ene-3β,20β-diol (**16**), 3β,11-dihydroxy-24-methylene-9,11-secocholestan-5-en-9-one (**23**), (24*S*)-ergostane-3β,5α,6β,25 tetraol (**146**), sinulerectadione (**227**), sinulerectol C (**229**), and cladieunicellin I (**277**) exhibited stronger cytotoxicity than their respective positive control and that their mechanism of action has not yet been further investigated.

## 1. Introduction

Marine environments, and especially coral reefs, are among the richest and most complex Earth ecosystems in terms of biodiversity [1]. Although their surface may appear small when compared to the total ocean floor, coral reefs provide a plethora of ecosystem goods and services [2,3] and represent the most important marine biodiversity hotspots [4]. Generally, biodiversity is the result of complex interactions between biotic and abiotic factors, resulting in a wide array of forms, behaviors, and consequently molecules. Over the years, the vast marine biodiversity has been largely investigated as a resource of therapeutic drugs with alternate success. Roughly 30,000 marine natural products are known so far and more than 1000 new compounds are isolated every year [5]. The oldest marine-derived drug of this type is cytarabine, which is the synthetic analog of sponge-isolated cytostatics spongothymidine and spongouridine. Cytarabine was approved as an antileukemic agent in 1969 and has been in clinical use for decades. At the present time, 17 marine drugs are approved, four are in phase III, nine in phase II, and 16 in phase I clinical trials [6]. Among these 46 molecules, 35 are anti-cancer agents. Corals have been the target of study for drug discovery since the nineteenth century and since the 70s, many natural products with diverse and important biological activities have been isolated and characterized. The phylum Cnidaria, and especially corals, accounts, along with Porifera, for 57% of the total bioactive marine compounds discovered [7]. However, despite the extreme coral biodiversity, none of these products have been placed under clinical evaluation yet. 

**Figure 1:**
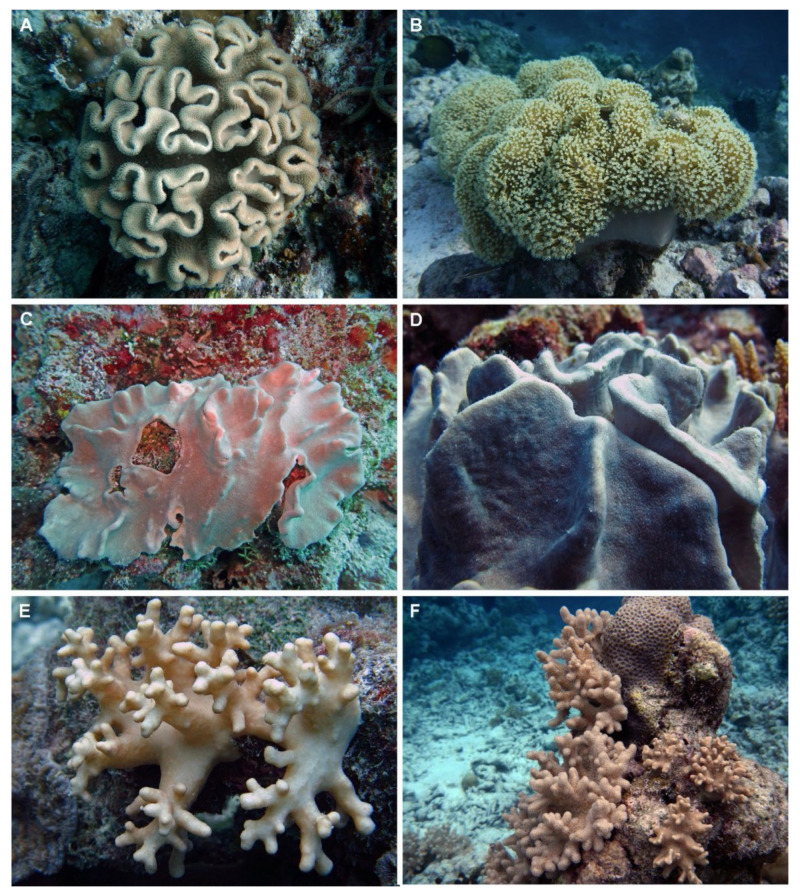
Overview of some of the most common soft corals found in Maldivian shallow reefs. (**A**,**B**) *Sarcophyton* sp. (**C**,**D**) *Lobophytum* sp. (**E**,**F**) *Sinularia* sp.

Corals mostly belong to the class Anthozoa, which is currently divided into two subclasses: Hexacorallia, including anemones, black corals, hard corals, and Octocorallia, including blue corals, sea pens, gorgonians, and soft corals [8]. Soft corals (Figure 1) have so far received more attention as potential sources of drug candidates, as they produce a greater number of secondary metabolites compared to hard corals. The most typical metabolites produced by soft corals are steroids, polyhydroxysterols, sesquiterpenes, diterpenoids, and biscembranoids [9]. These molecules may act as chemical defense compounds toward predators, competing reef organisms, and also against bacteria and parasites, to secure their protection and survival, and to control epibiont overgrowth [10]. For example, sarcophytoxide (**132**) is an allelopathic molecule usually found in *Sarcophyton* and used by this soft coral genus in the competition for space with scleractinian corals [11]; sarcophine (**121**) is another metabolite used for ecological defense, displaying toxic effects in fish [12]. In addition, several compounds may not be directly produced by the coral itself, but rather originate from or be made in concert with single-celled organisms (e.g., algae and bacteria) that act as symbionts and contribute significantly to the nutrition of their host. Production of these compounds may also be specific to the host-symbiont interaction, as the case of gogosterol, synthesized by zooxanthellae [13]. Recent studies also highlighted strong correlation between diversity of microbial communities and coral metabolome [14].

These compounds have been largely investigated given their potential to possess pharmacological properties of interest for human health [15]. Compounds extracted from soft corals have been frequently investigated in relation to their possible anticancer activity by testing their cytotoxic and antiproliferative activity against cancer cell lines, due the postulated natural function of these compounds as part of the chemical defense machinery of the producing organism or the host organism harboring the producing species [5]. In this framework, since the 2000s, the number of studies and scientific publications has grown exponentially, and consequently also the number of compounds isolated from soft corals with cytotoxic activity has reached considerable dimension. This is not surprising since cancer is a disease with a very high incidence in the developed countries, rising year by year and occupying the second ranking as cause for death after strokes. About 19.3 million new cancer cases and almost 10.0 million cancer deaths are estimated to have occurred in 2020 worldwide [16]. Currently, anticancer treatments involve the use of cytotoxic agents supplemented by targeted therapies to improve treatment efficacy and reduce side effects. The idea of using natural compounds to fight cancer is a long- and well-established tradition mainly originating from the availability of natural products and their traditional use that render public opinion open to their use as drugs. Indeed, almost 60% of drugs approved for cancer therapy are of natural origin, mostly from plants, such as vincristine (VCR), irinotecan etoposide, taxanes, and campthotecins, and from microbes, such as actinomycin D, mitomycin C, bleomycin, doxorubicin, and l-asparaginase [17]. In this context, the marine environment can be considered still underexplored, even if many papers that describe the application of marine metabolites are available, highlighting how the variety that populates the oceans overwhelms our discovery possibility. For instance, several halogenated compounds that are not present in the terrestrial environment were discovered in the sea [18]. Therefore, it is expected that future research, supported by the current knowledge, will bring to light new therapeutics.

Starting from this basis, in this review work, we focused on Alcyoniidae, one of the largest octocoral family belonging to the order Alcyonacea, with a circumglobal distribution, especially in the tropics and subtropics, and including several genera associated with intracellular symbiotic dinoflagellates [19]. Multiple genera in this family have been investigated for the presence of cytotoxic metabolites against cancer cell lines, namely *Lobophytum*, *Sarcophyton*, *Sinularia*, *Cladiella*, *Klyxum*, *Alcyonium*, *Bellonella*, *Anthomastus*, *Paraminabea* and *Protodendron*.

This literature review has been carried out using three different online databases (Scopus, Web of Science, and Google Scholar) and applying the following keywords: cytotoxic; anticancer, cytotoxicity; Alcyoniidae; *Lobophytum*; *Sarcophyton*; *Sinularia*; *Cladiella*; *Klyxum*. Among the retrieved papers, we selected only those reporting the full structural characterization of metabolites (i.e., with multi-dimensional NMR and HRMS data available) and the data regarding their cytotoxic activity against any type of cancer cell line evaluated with 3-(4,5-dimethylthiazol-2-yl)-2,5-diphenyl-2*H*-tetrazolium bromide (MTT) assay or a similar test related to the NAD(P)H-dependent oxidoreductase enzymes activity. As a result, a total of 164 articles were surveyed containing information related to 344 compounds. Most of the papers dated from 1992 to 2021. Data were then organized into three tables on the basis of the in vitro results of the MTT method [20,21] as reported in the related paper. 

Overall, the aim of the presented review was to provide researchers with a dataset handily exploitable for the selection of the most promising leads for further experiments and pre-clinical development studies, obtained from the actual body of knowledge regarding the last discovered cytotoxic compounds from soft corals under the lens of the same in vitro assay. Specifically, this work is the first providing a ranking of the cytotoxic coral metabolites based on the results of the same assay from the beginning of reporting of active compounds from Alcyoniidae.

## 2. Alcyoniidae as a Source of Cytotoxic Compounds 

### 2.1. Cytotoxic Compounds from the Genus Lobophytum

Whang et al. [22] investigated *Lobophytum michaelae***,** reporting the isolation of cembranolides, lobomichaolide (**1**) and crassolide (**2**). These molecules exhibited cytotoxicity against A-549 (human lung epithelial carcinoma), HT-29 (human colon adenocarcinoma), KB (human nasopharyngeal carcinoma), and P-388 (murine lymphocytic leukemia) cell lines with ED_50_ values of 0.38, 0.37, 0.59, and 0.34 μg/mL, respectively, for lobomichaolide, and with ED_50_ values of 0.39, 0.26, 0.85, and 0.08 μg/mL, respectively, for crassolide. 

An extract of *Lobophytum crassum*, investigated by Duh et al. [23], led to the isolation of a cembrane diterpene, lobocrassolide (**3**), and a cembrenolide, lobohedleolide (**4**). Lobocrassolide (**3**) was found to be cytotoxic for A549, HT-29, KB, and P-388 cancer cell lines with ED_50_ values of 2.99, 2.70, 2.91, and 0.012 μg/mL, respectively, while lobohedleolide (**4**) showed cytotoxicity only toward P-388 cells (ED_50_: 2.44 μg/mL). 

Five cembranoids, crassumolides A, B, D, E, and F, along with four known metabolites (including the aforementioned lobohedleolide) were isolated by Chao et al. [24] from *L. crassum*. The cytotoxicity of these compounds against human liver carcinoma (HepG2 and HepG3 cell lines), human breast carcinoma (MCF-7 and MDA-MB-231 cell lines), human lung carcinoma (A-549 cell line), and human gingival squamous cell carcinoma (Ca9–22 cell line) was evaluated, and the results showed that crassumolide A (**5**), crassumolide C (**6**), and lobohedleolide (**4**) exhibited cytotoxicity against Ca9-22 cells (IC_50_ values of 3.2, 1.7, and 2.8 μg/mL, respectively).

Four α-methylene-γ-lactone-bearing cembranoids, 20-acetylsinularolide B, presinularolide B, 3-dehydroxylpresinularolide B, and 3-dehydroxyl-20-acetylpresinularolide B, along with five known analogues (sinularolides B–E and 20-acetylsinularolide C), were isolated from *L. crassum* by Zhang et al. [25]. 3-dehydroxylpresinularolide B (**7**) showed cytotoxicity against A-549 and P-388 cell lines with 95.7% and 100% growth-inhibition at 10^−5^ mol/L, while sinularolide B (**8**) displayed cytotoxic activity toward P-388 cells with a growth-inhibition of 100% at 10^−5^ mol/L; cytotoxic activity was observed for presinularolide B (**9**) against A-549 cells and for sinularolide C (**10**) toward P-388 cells with 64.0% and 63.8% growth-inhibition at a concentration of 10^−5^ mol/L, respectively.

A macrocyclic diterpenoid, lobocrasol (**11**), was isolated from *L. crassum* and was found to be cytotoxic against P-388 cells with ED_50_ of 3.2 μg/mL [26].

Another chemical investigation of *Lobophytum* sp. resulted in the identification of a new squalene derivative, named lobophytene, three cembranoid diterpenes, and two sterols. Lobophytene (**12**) (IC_50_ values of 8.2 and 5.6 μM) and the cembranoid diterpene (1*S*,2*S*,3*E*,7*E*,11*E*)-3,7,11,15-cembratetraen-17,2-olide (**13**) (IC_50_ values of 5.1 and 1.8 μM) showed cytotoxic activity against lung (A-549) and colon (HT-29) cancer cell lines, respectively [27]. 

Cheng et al. [28] isolated five new cembranolides, durumolides M–Q from *Lobophytum durum* and found that durumolide P (**14**) exhibited cytotoxicity against P-388 cells with an ED_50_ of 3.8 μg/mL.

An unusual sterol including a 17,20-epoxy group, named lobophytosterol (**15**), was isolated from *Lobophytum laevigatum* along with (22*S*,24*S*)-24-methyl-22,25- epoxyfurost-5-ene-3β,20β-diol (**16**), and (24*S*)-24-methylcholest-5-ene-3β,25-diol (**17**). These compounds were found to exhibit cytotoxicity against HCT-116 (human colonic carcinoma) cells with IC_50_ values of 3.2 ± 0.9, 6.9 ± 0.8, and 18.1 ± 1.2 μM, respectively. Additionally, lobophytosterol (**15**) showed cytotoxicity toward A-549 (human lung carcinoma) and HL-60 (acute promyelocytic leukemia) cells with IC_50_ values of 4.5 ± 0.5 and 5.6 ± 0.4 μM, respectively [29].

Lobocrassin B (**18**) was found to be the most cytotoxic compound isolated by Kao et al. [30] from *Lobophytum crassum* among five cembrane-type diterpenoids (lobocrassins A–E), but it exhibited cytotoxicity against K-562 (human erythromyeloblastoid leukemia), CCRF-CEM (human T-cell acute lymphoblastic leukemia), Molt-4 (human acute lymphoblastic leukemia), and HepG2 (hepatocellular carcinoma) cells with IC_50_ values ranging from 0.34 to 3.44 μM. 

A 10-membered-ring diterpene, cyclolobatriene (**19**), along with three others diterpenes (with an already described structure), lobatriene (**20**), eunicol (**21**), and fuscol (**22**), were isolated from *Lobophytum pauciflorum* by Govindam et al. [31]. These natural products showed cytotoxic effects against human epidermoid carcinoma (A-431) cells with IC_50_ values of 0.64, 0.41, 0.35, and 0.52 μM, respectively.

Two diterpenes, lobocompactols A and B, and five known compounds were extracted from the methanol extract of *Lobophytum compactum* by Minh et al. [32]. Among them, 3β,11-dihydroxy-24-methylene-9,11-secocholestan-5-en-9-one (**23**) was found to exhibit cytotoxic activity against the A-549 cancer cell line with an IC_50_ of 4.97 ± 0.06 μM, while lobatrienolide (**24**), lobatriene (**20**), and (24*S*)-ergostane-3β,5α,6β,25-tetraol 25-monoacetate (**25**) showed activity with IC_50_ values of 23.03 ± 0.76, 31.13 ± 0.08, and 36.45 ± 0.01 μM, respectively. The cytotoxicity of 3β,11-dihydroxy-24-methylene-9,11-secocholestan-5-en-9-one on the A-549 cancer cells was comparable to that of the positive control, mitoxantrone (MX). Furthermore, all compounds exhibited cytotoxicity against the HL-60 cell line, with IC_50_ values ranging from 17.80 ± 1.43 to 59.06 ± 2.31 μM.

**Figure 2:**
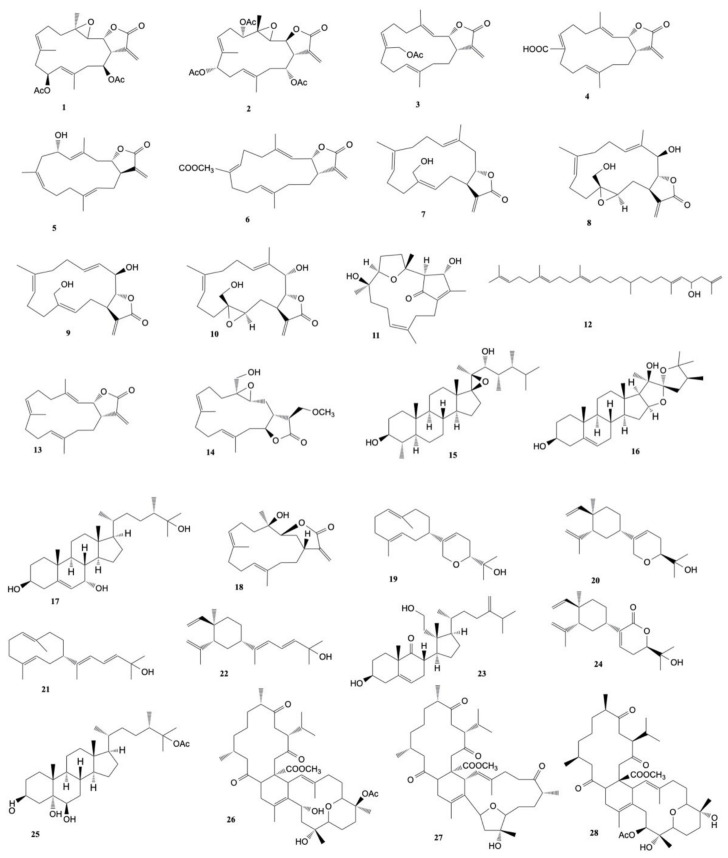
Cytotoxic compounds **1**–**28** derived from the genus *Lobophytum*.

Four cembranoids, namely laevigatols A–D, and six known metabolites, were isolated by Quang et al. [33] from coral *L. laevigatum.* The known ximaolide F (**26**), methyl tortuoate B (**27**), and nyalolide (**28**) exhibited cytotoxicity toward HL-60, A-549, and HCT-116 cell lines with IC_50_ values ranging from 9.0 to 28.7 μM. Moreover, nyalolide was cytotoxic toward MCF-7 cells with IC_50_ values of 35.5 μM. The structures of the cytotoxic compounds **1**–**28** derived from the genus *Lobophytum* are shown in Figure 2.

Six cembranolides, michaolides L–Q, and a known cembranolide, lobomichaolide, were isolated from the soft coral *L. michaelae* [34]. Lobomichaolide (**1**) and michaolides L, M, N, P, Q (**29**–**33**) were found to exhibit cytotoxicity against P-388, HT-29, and A-549 cells with ED_50_ values ranging from 0.3 to 4.9 μM/mL and the most potent effect of these compounds was against P-388 cancer cells (ED_50_ from 0.3 to 2.0 μM/mL).

(1*S*,2*S*,3*E*,7*E*,11*E*)-3,7,11,15-cembratetraen-17,2-olide (**13**), a known cembranoid diterpene isolated from *Lobophytum* sp., showed cytotoxic activity against HT-29 cells with IC_50_ value of 3.7 µM [35].

(1*S*,2*S*,3*E*,7*E*,11*E*)-3,7,11,15-cembratetraen-17,2-olide (**13**) was also investigated by Kim et al. [36] and exhibited cytotoxicity against human colorectal cancer cells (SNU-C5) with IC_50_ value of 3.7 µM.

Cembrene A (**34**), a cembranoid type diterpene isolated from *Lobophytum* sp. by Al-Footy et al. [37] showed cytotoxic activity against Ehrlich ascites carcinoma cells with LD_50_ of 50 μg/mL.

Roy et al. [38] isolated three compounds (**35**–**37**) from *Lobophytum* sp. showing cytotoxicity against HCT-116, with IC_50_ values of 135.37, 177.11, and 153.11 μM, respectively.

Three new polyoxygenated steroids, michosterols A–C, and four known compounds were isolated from *Lobophytum michaelae* by Huang et al. [39]. The cytotoxicity of the new metabolites against A-549 (human lung epithelial carcinoma), DLD-1 (human colon adenocarcinoma), and LNCap (human prostate adenocarcinoma) cells was evaluated, and the results showed that only michosterol A (**38**) exhibited a cytotoxicity effect against the A549 cell line with an IC_50_ value of 14.9 ± 5.7 μg/mL. 

Peng et al. [40] studied the soft coral *L. crassum*. They extracted and investigated two new cembrane-based diterpenoids together with ten known cembranoids. The known lobophylin (**39**), crassocolide E (**40**), sarcocrassocolide (**41**), 13-acetoxysarcocrassocolide (**42**), sarcocrassocolide M (**43**), 14-deoxycrassin (**44**), lobocrassin B (**18**), sarcocrassocolide F (**45**), and sarcocrassocolide G (**46**) showed anti-proliferative activity against diverse leukemia cell lines (K562, U937, Molt4, and Sup-T1) and 13-acetoxysarcocrassocolide (**42**), lobocrassin B (**18**), and 14-deoxycrassin (**44**) resulted the most effective with IC_50_ values ranging from 1.2 to 7.1 μg/mL. 

Zhang et al. [41] isolated four new polyhydroxylated steroids (lobophysterols A–D), together with two known related compounds (sarcophytosterol and klyflaccisteroid B), from the soft coral *Lobophytum* sp. Only lobophysterol D (**47**) showed cytotoxicity against HT-29 (colonic carcinoma), SNU-398 (hepatocellular carcinoma), and Capan-1 (pancreatic carcinoma) cells with IC_50_ values ranging from 21.56 to 38.83 μM, while lobophysterol B (**48**) was found to exhibit cytotoxicity against SNU-398 cancer cells with IC_50_ value of 40.04 μM.

Seven new cembrane-type diterpenes, lobophytolins C–I, and one new prenylated-guiane-type diterpene, lobophytolin J, along with six known compounds, have been isolated from *Lobophytum* sp. by Li et al. [42]. Lobophytolin D (**49**) exhibited cytotoxicity toward HT-29, Capan-1, A-549, and SNU-398 human cancer cell lines with IC_50_ values of 4.52 ± 0.82, 6.62 ± 4.02, 5.17 ± 0.86, and 6.15 ± 2.88 μM, respectively, while lobophytolin C (**50**) and lobophytolin J (**51**) showed cytotoxicity against SNU-398 with IC_50_ values of 42.54 ± 6.26 and 26.85 ± 19.97 μM, respectively.

**Figure 3:**
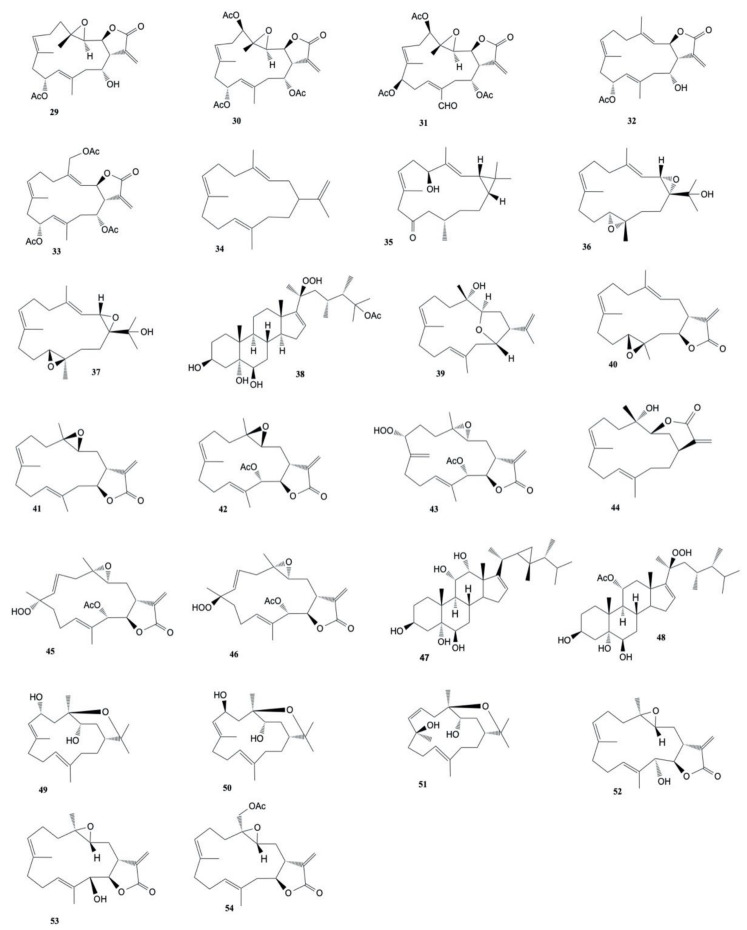
Cytotoxic compounds **29**–**54** derived from the genus *Lobophytum*.

The known cembranoids durumolide J (**52**), lobophytolide D (**53**), and lobolide A (**54**), isolated from *L. crassum* by Yin et al. [43], were found to exhibit cytotoxic activity against A549, HT-29, SNU-398 and Capan-1 cancer cell lines with IC_50_ values ranging from 1.5 to 7.4 μmol/L. The structures of the cytotoxic compounds **29**–**54** derived from the genus *Lobophytum* are shown in Figure 3.

### 2.2. Cytotoxic Compounds from the Genus Sarcophyton

The genus *Sarcophyton* is by far the most studied source of bioactive compounds among soft corals, and several scientific papers dealt with cytotoxic compounds extracted from this taxon. The very early and iconic example dates back to 1989 with the discovery by Fujiki et al. [44] of sarcophytol A (**55**) from *Sarcophyton* sp. in Ishigaki Island (Okinawa, Southern Japan). 

Duh et al. [45] studied the cytotoxicity of four cembrenolide diterpenes, sarcocrassolide (**56**), crassolide (**2**), 13-acetoxysarcocrassolide (**57**), and denticulatolide (**58**), and two steroids isolated from the soft coral *Sarcophyton crassocaule*. The results showed that the cembrenolide diterpenes and the steroid (24*S*)-24-methylcholestane-3β,5α,6β-triol (**59**) exhibited cytotoxicity against P-388 (mouse lymphocytic leukemia) cancer cells with ED_50_ values ranging from 0.14 to 0.38 μg/mL and a growth inhibition on A-549 (human lung epithelial carcinoma), HT-29 (human colon adenocarcinoma), and KB (nasopharyngeal carcinoma) cell lines with ED_50_ values ranging from 4.29 to 9.15 μg/mL. Instead, the other steroid, 24ξ-methylcholestane-3β,5α,6β,25-tetraol-25-monoacetate (**60**), was effective only on P-388 (ED_50_ of 3.96 μg/mL) and HT-29 (ED_50_ of 4.32 μg/mL) cancer cell lines. 

Ten different compounds extracted from the soft coral *Sarcophyton trocheliophorum* were tested for cytotoxicity against HL-60 (human leukemia), M-14 (skin melanoma), MCF-7 (breast carcinoma) cancer cells, and normal human peripheral blood lymphocytes. Only the steroid 23,24-dimethylcholest-16(17)-*E*-en-3β,5α,6β,20(*S*)-tetraol (**61**) was found to exhibit cytotoxicity on cancer cell lines with EC_50_ values ranging from 2.8 to 4.9 μg/mL and it also showed toxicity to human lymphocytes [46].

*Sarcophyton cherbonnieri* was investigated by Gross et al. [47] and the chromatographic separation of the extracts yielded three new furano-cembranoids, 13-dehydroxysarcoglaucol (**62**), sarcoglaucol-16-one (**63**), and decaryol (**64**) that were found to be cytotoxic against HM02 (gastric adenocarcinoma), HepG2 (hepatocellular carcinoma), and MCF-7 (breast adenocarcinoma) cell lines with GI_50_ values ranging from 0.15 to 8.6 µg mL^−1^.

Two tetracyclic tetraterpenoids, methyl tortuoate A and methyl tortuoate B, along with the known methyl sartortuoate, were isolated from *Sarcophyton tortuosum* by Zeng et al. [48]. Methyl tortuoate A (**65**) and methyl tortuoate B (**27**) exhibited cytotoxicity against the human nasopharyngeal carcinoma CNE-2 cell line, with IC_50_ values of 22.7 and 24.7 μg/mL, and the murine P-388 cancer cell line, with IC_50_ values of 3.5 and 5.0 μg/mL, respectively.

Six polyoxygenated cembrane-based diterpenoids, crassocolides A–F, were isolated along with the known epoxycembranolide from the ethyl acetate extract of *S. crassocaule* [49]. 13-acetoxy-3,4-dihydroxycembranolide (crassocolide A (**66**)) and crassocolide F (**67**) were found to be cytotoxic (IC_50_ values ranging from 3.1 to 11.9 μg/mL) against HepG2 (human hepatocellular carcinoma), MCF-7 (human breast carcinoma), MDA-MB-231 (human breast adenocarcinoma), and A-549 (human lung epithelial carcinoma), respectively. Moreover, the known epoxycembranolide, lobophytolide (**53**) showed cytotoxicity against HepG2 cells (IC_50_ = 6.3 μg/mL) and cytotoxicity (IC_50_ ≈ 2.0 μg/mL) against the other cancer cell lines.

A known natural product (emblide) and five new cembrane diterpenes (sarcrassins A–E) were isolated from *S. crassocaule* by Zhang et al. [50]. Sarcrassin B (**68**), sacrassin D (**69**), and emblide (**70**) exhibited cytotoxicity against KB cell lines with IC_50_ values of 5.0, 4.0, and 5.0 μg/mL, respectively, while sarcrassin A (**71**) and sarcrassin E (**72**) showed activity with IC_50_ values of 19.0 and 13.0 μg/mL, respectively.

Seven new 14-membered carbocyclic cembrane, sarcostolides A–G (73–79), isolated by Cheng et al. [51] from *Sarcophyton stolidotum*, exhibited cytotoxic activity against HeLa (human cervical epithelioid carcinoma) and WiDr (human colon adenocarcinoma) cell lines. Moreover, sarcostolide E (**75**) showed cytotoxicity against Daoy with ED_50_ at 5.5 μg/mL, while others were inactive. 

**Figure 4:**
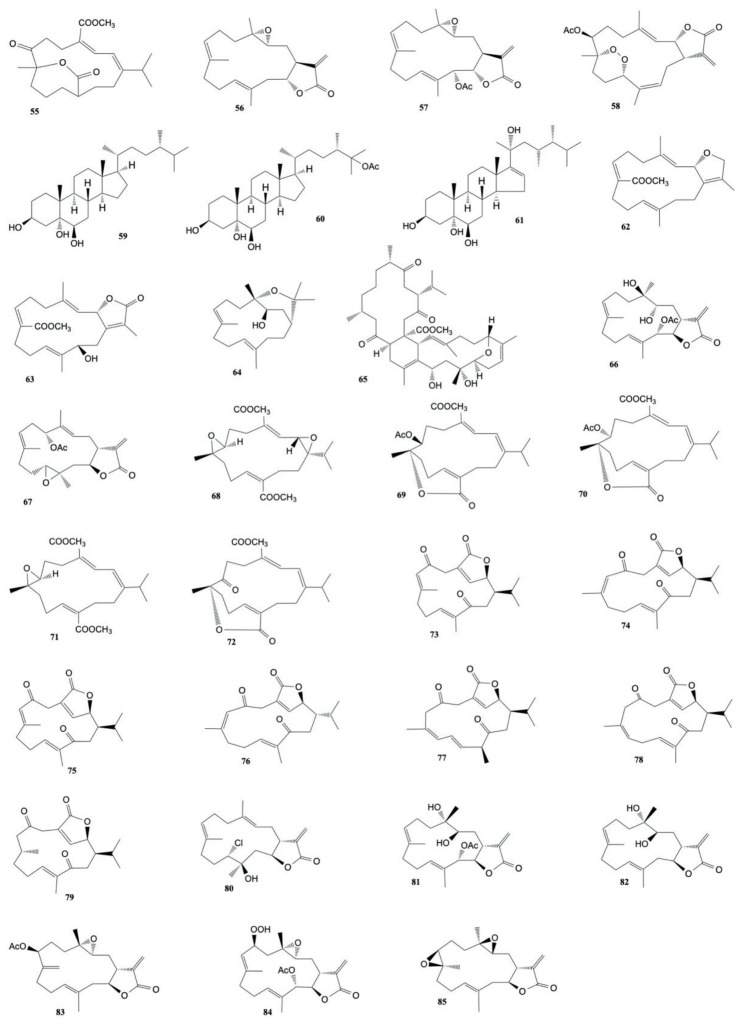
Cytotoxic compounds **55**–**85** derived from the genus *Sarcophyton*.

The cytotoxicity of polyoxygenated cembranoids (crassocolides G–M) isolated by Huang et al. [52] from *S. crassocaule*, against KB, Hela, and Daoy cells was studied. The results displayed that crassocolides H–M (**80**–**85**) showed cytotoxicity against Daoy cells where crassocolides I (**81**) and M (**85**) were found to be more active (IC_50_ 0.8 ± 0.1 and 1.1 ± 0.2 µg/mL, respectively). Moreover, crassocolides H (**80**) and L (**84**) were found to be active against the growth of KB and cells. The structures of the cytotoxic compounds **55**–**85** derived from the genus *Sarcophyton* are shown in Figure 4.

Sarcocrassolides A–D (**86**–**89**) exhibited cytotoxic activity against human breast carcinoma (MCF-7), human colon carcinoma (WiDr), human laryngeal carcinoma (HEp-2), and human medulloblastoma (Daoy) with ED_50_ values ranging from 1.2 to 8.8 μM) [53]. Sarcocrassolides F–I (**90**–**93**) were also found to exhibit cytotoxicity against Daoy, HEp-2, MCF-7, and WiDr cells. Among them, compound I (**93**) (ED_50_ values of 5.1, 5.8, 8.4, and 6.4 μM) was the most potent one [54]. Moreover, sarcocrassolides P-R (**94**–**96**) showed cytotoxicity against human colon adenocarcinoma (DLD-1), human T-cell acute lymphoblastic leukemia (CCRF-CEM), and human promyelocytic leukemia (HL-60) cell lines with sarcocrassolide R being the most cytotoxic [55]. Lin et al. also analyzed the cytotoxicity of crassocolides A (**66**), B (**97**), D (**98**), and E (**40**), which resulted to be more active than sarcocrassolides P-R except for the compound D. 

7β-acetoxy-8α-hydroxydeepoxysarcophine (**99**), isolated from *Sarcophyton glaucum*, was found to be cytotoxic against HepG2, HCT-116, and HeLa cells with IC_50_ values of 3.6 ± 1.0, 2.3 ± 1.5, and 6.7 ± 0.8 μg/mL, respectively [56].

Three new cembranoids, crassocolides N–P, were isolated from the organic extract of *S. crassocaule* by Wang et al. [57]. Crassocolide N (**100**) was found to exhibit cytotoxic activity against KB, HeLa and Daoy cells (IC_50_ of 4.7, 4.7, and 2.8 μg/mL, respectively); crassocolide O (**101**) showed cytotoxic against Daoy cells (IC_50_: 4.5 μg/mL), similarly to crassocolide P (**102**) (IC_50_: 1.9 μg/mL).

Su et al. [58] investigated the cytotoxic effects of 13-acetoxysarcocrassolide (**57**), isolated from *S. crassocaule*, on bladder female transitional cancer (BFTC) cells. The concentration of 13-acetoxysarcocrassolide, ranging from 0.5 μg/mL to 5 μg/mL dose-dependently, inhibited the growth of BFTC cells: the population of cancer cells was clearly reduced after treatment with 1.5 and 3 μg/mL.

Three new cembranoids, sarcocrassocolides M–O (**103**–**105**), were isolated from *S. crassocaule* [59]. The natural products were found to be cytotoxic towards MCF-7, HEp-2, and Daoy cancer cells with ED_50_ values ranging from 5.0 ± 0.7 to 12.4 ± 2.1 μM.

Whang et al. [60] investigated on the acetone extract of *Sarcophyton ehrenbergi* and isolated two new diterpenoids: ehrenbergol C (**106**) and acetyl ehrenberoxide B (**107**) that exhibited cytotoxicity against P-388 cells with EC_50_ of 25.9 and 24.7 µg/mL, respectively.

Chemical investigation of *S. glaucum* [61] led to the isolation of 11 isoprenoid metabolites: a new sesquiterpenoid (6-oxo-germacra-4(15),8,11-triene), a new natural cembranoid (sarcophinediol), along with two known sesquiterpenoids and seven known cembranoids. Sarcophinediol (**108**) and sarcotrocheliol acetate (**109**) were found to be cytotoxic toward HepG2 with IC_50_ values of 18.8 ± 0.07 and 19.9 ± 0.02 μM, respectively; moreover, deoxosarcophine (**110**), sarcotrocheliol acetate (**109**), and sarcotrocheliol (**111**) exhibited cytotoxicity against MCF-7 cells with IC_50_ values of 9.9 ± 0.03, 2.4 ± 0.04 and 3.2 ± 0.02 μM, respectively; finally, 6-oxo-germacra-4(15),8,11-triene, sarcophinediol (**108**), and deoxosarcophine (**110**) showed activity towards HCT-116 cells with IC_50_ values of 29.4 ± 0.03, 19.4 ± 0.02, and 25.8 ± 0.03 μM, respectively. 

**Figure 5:**
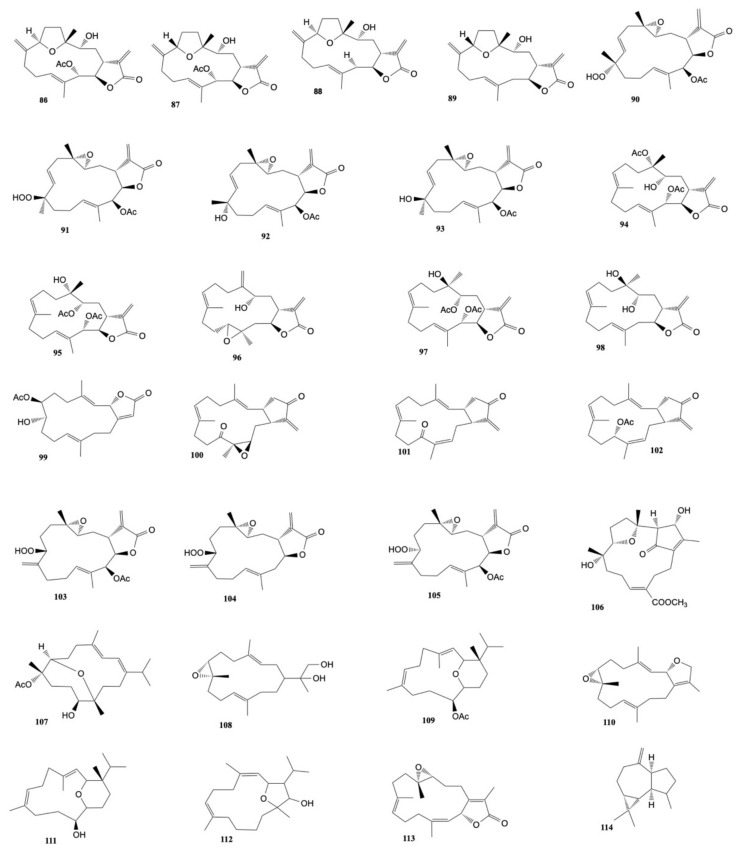
Cytotoxic compounds **86**–**114** derived from the genus *Sarcophyton*.

Three new cembranoids, sarcophytolol, sarcophytolide B, and sarcophytolide C, along with three known metabolites, 10(14)aromadendrene, deoxosarcophine, and sarcophine, were obtained from *S. glaucum* [62]. Sarcophytolol (**112**), sarcophytolide C (**113**), and 10(14)aromadendrene (**114**) were found to exhibit similar cytotoxic effects towards HepG2 cancer cells (IC_50_ of 20 μM); sarchophytolides C (**113**) showed cytotoxicity against MCF-7 cancer cells (IC_50_: 25 ± 0.0164 and 29 ± 0.030 μM, respectively); finally, 10(14)aromadendrene (**114**) exhibited cytotoxicity towards PC-3 (Prostate cancer) cells with IC_50_ of 9.3 ± 0.164 μM). The structures of the cytotoxic compounds **86**–**114** derived from the genus *Sarcophyton* are shown in Figure 5.

A chemical investigation of *Sarcophyton auritum* [63] led to the isolation of two new cembranoids, 2-epi-sarcophine and (1*R*,2*E*,4*S*,6*E*,8*R*,11*R*,12*R*)-2,6-cembradiene-4,8,11,12-tetrol, along with two known diterpene cembranoids, sarcophine, and (+)-7α,8β-dihydroxydeepoxysarcophine. (+)-7α,8β-dihydroxydeepoxysarcophine (**115**) was found to possess the highest cytotoxic activity against both breast (MCF-7) and liver (HepG2) cancer cell lines with IC_50_ values of 11 ± 0.22 and18.4 ± 0.16 μg/mL, respectively.

Glaucumolides A and B, novel biscembranes composed of an unprecedented α,β-unsaturated ε-lactone, were isolated from *Sarcophyton glaucum* by Huang et al. [64]. Glaucumolide B (**117**) was found to be cytotoxic against HL-60 (promyelocytic leukemia) and CCRF-CEM (T-cell acute lymphoblastic leukemia) human cancer cell lines with ED_50_ values of 3.8 ± 0.9 and 5.3 ± 1.4 μg/mL, respectively. Moreover, glaucumolide A (**116**) showed cytotoxicity toward the above two cell lines with ED_50_ values of 6.6 ± 1.2 and 7.4 ± 1.5 μg/mL, respectively. Moreover, glaucumolides A (**116**) and B (**117**) exhibited cytotoxicity against Molt-4 (acute lymphoblastic leukemia) and K-562 (chronic myelogenous leukemia) human cancer cell lines (ED_50_ 11.0–19.2 μg/mL). 

Chemical investigation of *S. glaucum* led to the isolation of four diterpenes (sarcophinone, 8-epi-sarcophinone, (+)-7α,8β-dihydroxydeepoxysarcophine (**115**), sinumaximol G, and (+)-sarcophine), the sesquiterpene prostantherol, and the sterol 3β,24S-ergost-5-en-ol. The compounds were tested against HepG2 cancer cell lines. Sarcophinone (**118**) and 8-epi-sarcophinone (**119**) were tested together and exhibited an EC_50_ value of 11.32 μg/mL, while (+)-7α,8β-dihydroxydeepoxysarcophine (**114**), sinumaximol G (**120**), sarcophine (**121**), and prostantherol (**122**) showed EC_50_ values of 17.84, 9.97, 10.32, and 12.22 μg/mL, respectively [65]. 

Three new cembrene diterpenoids, sarcoehrenbergilid A–C (**123**–**125**), along with four known diterpenoids, sarcophine (**121**), (+)-7α,8β-dihydroxydeepoxysarcophine (**115**), sinulolide A (**126**), sinulolide B (**127**), and one steroid, sardisterol (**128**) were isolated and characterized by Hegazy et al. [66] from *Sarcophyton ehrenbergi*. The cytotoxicity of the natural products was tested against HepG2, A-549, and Caco-2 cancer cell line and sardisterol (**128**) resulted the most potent of the metabolites isolated with an IC_50_ of 27.3 µM against the A-549 cancer cell line. 

Chao et al. [67] isolated 15 compounds from EtOAc extract of *S. glaucum*. This study showed that sarcomilasterol (**129**) exhibited a cytotoxic effect against MDA-MB-231 (human breast adenocarcinoma), MOLT-4 (human acute lymphoblastic leukemia), SUP-T (human T-cell lymphoblastic lymphoma), and U-937 (human histiocytic lymphoma) cell lines with IC_50_ values of 13.8, 6.7, 10.5, and 17.7 μg/mL, respectively, while sarcoaldesterol B (**130**) was found to possess cytotoxicity against HepG2, MDA-MB-231, and A-549 cell lines with IC_50_ values of 9.7, 14.0, and 15.8 μg/mL.

Analyzing *Sarcophyton* sp., Januar et al. [68] found a new cembranoid, namely, 2-hydroxy-crassocolide E (**131**), along with five known cembranoids: sarcophytoxide (**132**), sarcrassin E (**72**), 3,7,11-cembreriene-2,15-diol (**133**), 11,12-epoxy-sarcophytol A (**134**), and sarcophytol A (**55**). All these cembranoids were found to inhibit the growth of MCF-7 (human breast cancer) cells, with IG50 18.13 ppm, 12.22 ppm, 24.2 ppm, 22.27 ppm, 18.88 ppm, and 20.041 ppm, respectively.

Eight new cembrane-type diterpenoids were isolated from the soft coral *Sarcophyton mililatensis* by Li et al. [69] together with two known related analogues, (+)-isosarcophytoxide and (+)-isosarcophine. In in vitro bioassays, (+)-isosarcophytoxide (**135**) displayed cytotoxicity against human promyelocytic leukemia cells (HL-60) and human lung adenocarcinoma cells (A-549) with IC_50_ values of 0.78 ± 0.21 and 1.26 ± 0.80 μmol/L, respectively. 

**Figure 6:**
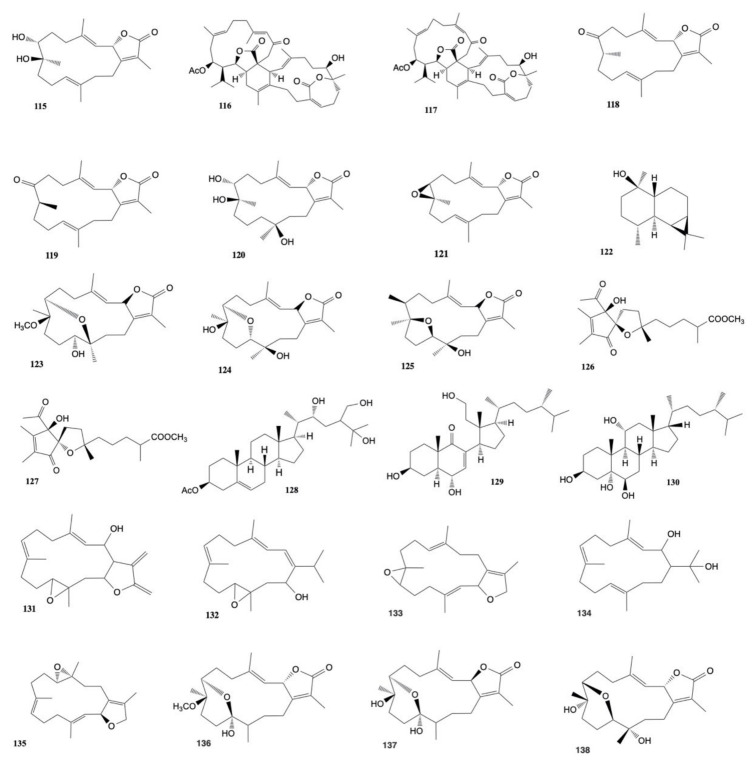
Cytotoxic compounds **115**–**138** derived from the genus *Sarcophyton*.

Hegazy et al. [70] isolated three cembrene diterpenoids, sarcoehrenbergilides D–F (**136**–**138**), from *S. ehrenbergi*. The compounds showed the highest cytotoxic activity toward A549 cells with IC_50_ values of 23.3, 27.3, and 25.4 μM, respectively. Moreover, sarcoehrenbergilides E, F (**137**,**138**) also exhibited activity toward HpG2 human cancer cell lines with IC_50_ values of 22.6 and 31.8 μM, respectively. The structures of the cytotoxic compounds **115**–**138** derived from the genus *Sarcophyton* are shown in Figure 6.

Hassan et al. [71] isolated five new cembrane-type diterpenoids, 7-acetyl-8-epi-sinumaximol G (**139**), 8-epi-sinumaximol G (**140**), 12-acetyl-7,12-epi- sinumaximol G (**141**), 12-hydroxysarcoph-10-ene (**142**), and 8-hydroxy-epi-sarcophinone (**143**), along with two known analogs (sinumaximol G (**120**) and sarcophine (**121**)) from *Sarcophyton* species. All the tested compounds had cytotoxic potential against MCF-7 with IC_50_ values of 22.39–27.12 µg/mL, with a slightly reduced cytotoxic effect in comparison to the standard anticancer drug doxorubicin (IC_50_: 12.78 µg/mL), highlighting the role of the α, β-unsaturated-γ-lactone moiety for the antiproliferative properties of these compounds.

Chemical investigation of *Sarcophyton digitatum* resulted in the isolation of four new biscembranoidal metabolites, sardigitolides A–D, along with three previously isolated biscembranoids, sarcophytolide L, glaucumolide A, and glaucumolide B, and two known cembranoids. Sardigitolide B (**144**) was found to be cytotoxic against MCF-7 and MDA-MB-231 cells with IC_50_ values of 9.6 ± 3.0 and 14.8 ± 4.0 μg/mL, respectively, and glacumolide A (**116**) showed cytotoxicity toward MCF-7, HepG2, and HeLa cell lines with IC_50_ values of 10.1 ± 3.3, 14.9 ± 3.5, and 17.1 ± 4.5 µg/mL, respectively. Moreover, glacumolide B (**117**) resulted cytotoxic toward MCF-7, MDA-MB-231, and HepG2 cell lines, with IC_50_ values of 9.4 ± 3.0, 17.8 ± 4.5, and 14.9 ± 4.2 µg/mL, respectively. Moreover, isosarcophytonolide D (**145**) showed cytotoxic activity against the growth of MCF-7 cancer cell line with IC_50_ value of 10.9 ± 4.3 µg/mL [72].

Two new polyhydroxy sterols, acutumosterols A and B, and four known analogues were extracted from the EtOAc fraction of *Sarcophyton acutum* by Zidan et al. [73]. The known (24*S*)-ergostane-3β,5α,6β,25 tetraol (**146**) displayed cytotoxic activity against HepG2 cancer cell line (IC_50_ of 17.2 ± 1.5 μg/mL), comparable to the standard cytotoxic agent etoposide (IC_50_ of 17.6 ± 0.2 μg/mL). Moreover, the new acutumosterol A (**147**) and the known sarcoaldosterol B (**148**) exhibited cytotoxic activity toward MCF-7 (IC_50_ 28.8 ± 1.9 and 30.2 ± 4.0 μg/mL, respectively) and A549 (IC_50_ 27.4 ± 1.2 and 24.8 ± 2.8 μg/mL, respectively) cancer cell lines. The other compounds showed cytotoxic activity (IC_50_ 31.6–57.2 μg/mL).

Chemical investigation of *Sarcophyton tenuispiculatum* resulted in the isolation of 1,4-dihydrobenzoquinone, sarcotenuhydroquinone, three new cembranoids, namely sarcotenusenes A–C, and ten known metabolites. Sarcotenuhydroquinone (**149**), sarcotenusene A (**150**), (2*S*,7*S*,8*S*)-sarcophytoxide (**151**), (2*S*,7*R*,8*R*)-sarcophytoxide (**152**), sarcophytonin F (**153**), 3,4-dihydro-4α-hydroxy-∆2-sarcophine (**154**), and a hydroperoxide (obtained by autoxidation of dihydrofuranocembranoid) (**155**) exhibited cytotoxicity against MCF-7 cells with IC_50_ values of 25.3 ± 2.8, 34.3 ± 3.7, 37.6 ± 4.2, 33.3 ± 3.5, 30.1 ± 3.1, 24.3 ± 3.0, and 27.2 ± 4.0 µM, respectively. Moreover, compound (2*S*,7*S*,8*S*)-sarcophytoxide (**151**), (2*S*,7*R*,8*R*)-sarcophytoxide (**152**), 3,4-dihydro-4α-hydroxy-∆2-sarcophine (**154**), and the hydroperoxide (**155**) showed cytotoxicity against HepG2 cells with IC_50_ values of 35.2 ± 4.4, 28.6 ± 3.4, 34.5 ± 4.2, and 36.4 ± 5.3 µM respectively. Furthermore, compounds sarcotenuhydroquinone (**149**) and sarcophytonin F (**153**) resulted cytotoxic toward the MDA-MB-231 cell line with an IC_50_ value of 36.4 ± 3.6 and 38.6 ± 5.0 µM, respectively [74].

**Figure 7:**
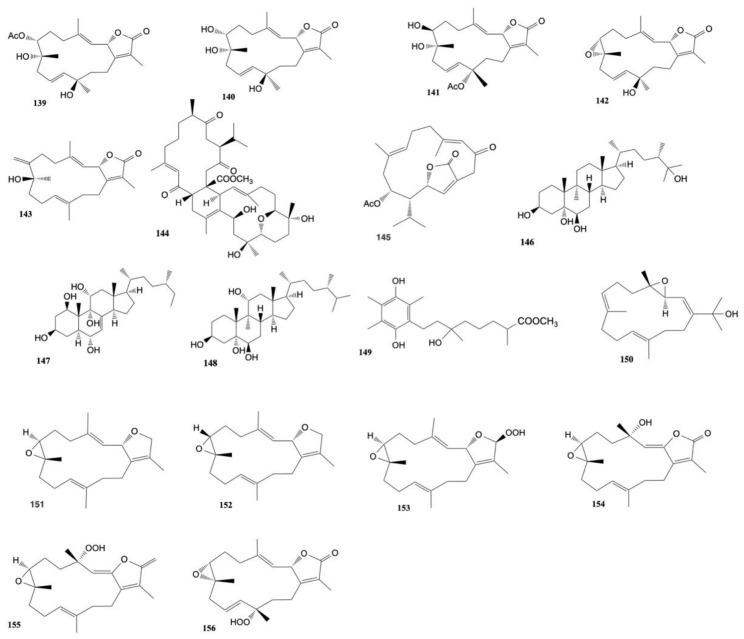
Cytotoxic compounds **139**–**156** derived from the genus *Sarcophyton*.

Five oxygenated cembrenoids, sarcoconvolutum A–E, were extracted from *Sarcophyton convolutum*. Only sarcoconvolutum D (**156**) showed cytotoxicity against A-549, HeLa, and HSC2 cancer cell lines with IC_50_ values of 49.70 ± 0.05, 91.98 ± 0.15, and 53.17 ± 0.03 µM, respectively [75]. The structures of the cytotoxic compounds **139**–**156** derived from the genus *Sarcophyton* are shown in Figure 7.

### 2.3. Cytotoxic Compounds from the Genus Sinularia

The genus *Sinularia* includes an estimated number of ~190 nominal species, 47 of which have been described only in the last 25 years [76]. Many of these species have been chemically evaluated, including hybrid species. It is by far the most studied genus of corals as a resource of metabolites with potential use as anticancer drugs. 

The diterpene sinugibberol (**157**), extracted from *Sinularia gibberosa* by Hou et al. [77] induced cytotoxicity to HT-29 (human colon adenocarcinoma) cells (ED_50_ of 0.5 μg/mL) and activity against the P-388 (murin lymphocytic leukemia) cell line (ED_50_ of 11.7 μg/mL).

Four new cytotoxic cembranoid diterpenes were extracted from *S. gibberosa* by Duh and Hou [78] and exhibited cytotoxicity toward the growth of A-549 (human lung adenocarcinoma), HT-29 (human colon adenocarcinoma), KB (human epidermoid carcinoma), and P-388 (murine lymphocytic leukemia) cell lines. 11,12-epoxy-(1*E*,3*E*,7*E*)-cembratrien-15-ol (**158**) resulted in the most potent of the metabolites with IC_50_ values ranging from 0.01 to 1.03 μg/mL. 

A new heptacyclic norcembranoid dimer, singardin (**159**), and a known sesquiterpene, guaianediol (**160**), isolated from *Sinularia gardineri* by El Sayed and Hamann [79] exhibited cytotoxicity toward P-388, A-549, HT-29, and MEL-28 cancer cell lines with values ranging from 1 to 5 μg/mL. 

Duh et al. [80] isolated three cembranoid diterpenes, sinuflexolide, dihydrosinuflexolide, and sinuflexibilin from *Sinularia flexibilis*. Sinuflexolide (**161**) and sinuflexibilin (**162**) were cytotoxic for A549, HT-29, KB, and P-388 cancer cells with ED_50_ values ranging from 0.16 to 1.73 μg/mL.

A new sterol 7β-hydroperoxy-24-methylenecholesterol along with five known compounds, sarcophytol A (**55**), (*Z*)-*N*-[2-(4-hydroxyphenyl)ethyl]-3-methyldodec-2-enamide, 5α,7α*H*-eduesm-11(13)en-4α-ol, 24-methylenecholesterol, and 1β-hydroxy-α-cyperone were isolated from *Sinularia* sp. by Sheu et al. [81]. 7β-hydroperoxy-24-methylenecholesterol (**163**), sarcophytol A (**55**), and 1β-hydroxy-α-cyperone (**164**) resulted cytotoxic against P-388 cancer cells with EDs values of 2.6, 1.3 and 2.9 μg/mL, respectively.

Chemical investigation on the soft coral *Sinularia capillosa* led to the isolation of a new cembranolide (capillolide) and three known compounds (sinulariolide (**165**), flexibilide (**166**), and (1*R*,13*S*,12*S*,9*S*,8*R*,5*S*,4*R*)-9-acetoxy-5,8:12,13-diepoxycembr-15(17)-en-16,4-olide (**167**)) that exhibited cytotoxic activity against P-388 and L1210 (mouse lymphocytic leukemia) cell lines. Capillolide (**168**) was found to have IC_50_ values of 15.0 and 18.5 μg/mL, respectively, while the known metabolites gave IC_50_ values ranging from 1.5 to 10.0 μg/mL [82].

A lobane diterpene, ineleganene (**169**) isolated from a hexane extract of *Sinularia inelegans* exhibited cytotoxicity against A-549 cells (GI_50_ of 3.63 μg/mL) and P-388 cells (GI_50_ of 0.20 μg/mL) [83].

A new sterol, (22*R*,23*R*,24*R*)-5α,8α-epidioxy-22,23-methylene-24-methylcholest-6-en-3β-ol (**170**), as well as two known sterols, numersterol A (**171**) and pregnenolone (**172**), isolated from *Sinularia* sp. [84] were found to exhibit cytotoxicity toward the growth of P-388, KB, A-549, and HT-29 cells with IC_50_ values ranging from 0.4 to 10.8 μg/mL.

Four new norditerpenoids, scabrolides A–D, along with four known metabolites (norditerpenes 5–8), were isolated from the dichloromethane extract of *Sinularia scabra*. Norditerpenes 6 (**173**) and 7 (**174**) were found to be cytotoxic against the growth of KB (human oral epidermoid carcinoma) and Hepa 59T/VGH (human liver carcinoma) cells with ED_50_ values ranging from 2.3 to 2.6 μg/mL, whereasscabrolide C (**176**) exhibited only activity toward KB and Hepa 59T/VGH cells and norditerpene 8 (**175**) was found to display selective activity against Hepa 59T/VGH cells. The remaining metabolites were shown to be inactive (ED_50_ > 20 μg/mL) to both cell lines [85].

A chemical investigation on the dichloromethane extract of *Sinularia parva* led to the isolation of three new bicyclic norcembranoids, leptocladolide A, 1-epi-leptocladolide A, and 7*E*-leptocladolide A [86]. Leptocladolide A (**177**) exhibited cytotoxicity against KB and Hepa 59T/VGH cancer cells lines with ED_50_ values ranging from 2.6 to 15.1 μg/mL.

A novel steroid, gibberoketosterol, along with four known steroids, were extracted from *Sinularia gibberosa* by Ahmed et al. [87]. Gibberoketosterol (**178**) and two of the known steroids showed a cytotoxicity against the growth of Hepa 59T/VGH cancer cells with ED_50_ values of 10.0, 9.3, and 6.8 μg/mL, respectively.

Ahmed et al. [88] also isolated three new norditerpenoids, scabrolides E–G, along with dissectolide A, from *Sinularia scabra.* The tricyclic norditerpenoid scabrolide E (**179**) was found to be cytotoxic against the proliferation of Hepa 59T/VGH and KB cells with ED_50_ values of 0.5 and 0.7 μg/mL, respectively.

Nanolobatin A (**180**) and nanolobatin B (**181**), two new norsesquiterpenoids isolated by Ahmed et al. [89] from *Sinularia nanolobata*, were found to be cytotoxic toward KB and Hepa 59T/VGH cancer cell lines with ED_50_ values ranging from 4.5 to 8.3 μg/mL.

The chemical investigation of *Sinularia microclavata,* by Zhang et al. [90] yielded a new diterpenoid (microclavatin) and a known cembranolide (capillolide). Microclavatin (**182**) was found to be cytotoxic against cancer cell lines KB and MCF-7 with IC_50_ values of 5.0 and 20.0 μg/mL, respectively, while capillolide (**168**) showed cytotoxicity against A-549 cancer cell lines with an IC_50_ value of 0.5 μg/mL.

**Figure 8:**
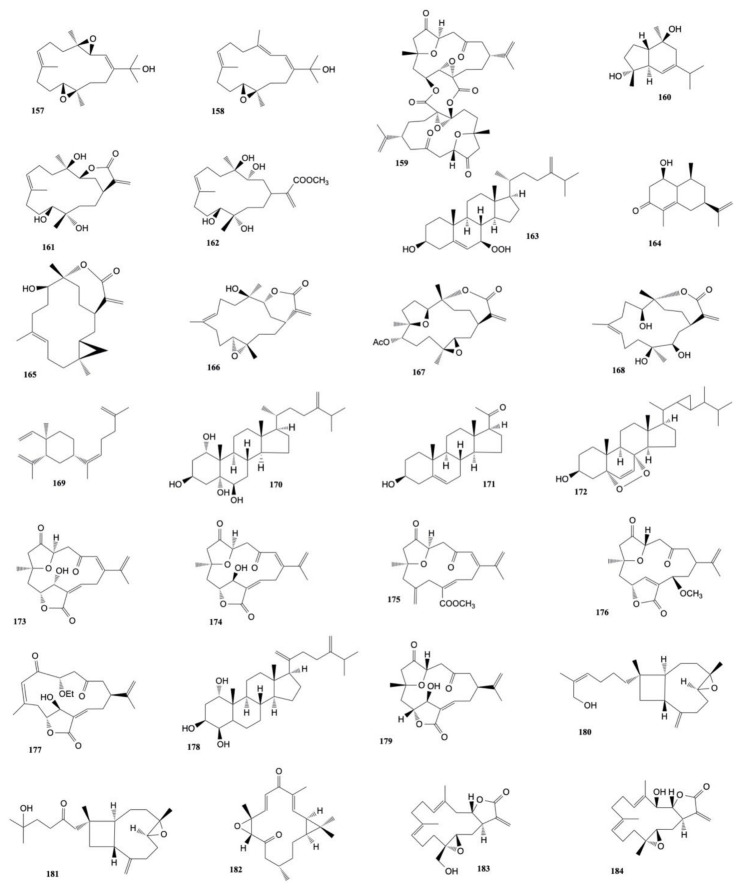
Cytotoxic compounds **157**–**184** derived from the genus *Sinularia*.

Li et al. [91] isolated five new α-methylene-γ-lactone-bearing cembranoid diterpenes, sinularolides A–E, from *S. gibberosa*. Sinularolides B–E (**8, 10, 183, 184**) were found to exhibit cytotoxicity toward HL-60 (human leukemia), BGC-823 (human gastric), and MDA-MB-435 (human breast), cancer cells with IC_50_ values ranging from 2.1 to 8.6 μg/mL. The structures of the cytotoxic compounds **157**–**184** derived from the genus *Sinularia* are shown in Figure 8.

A new 9,11-secosteroid, 3β,11-dihydroxy-24-methylene-9,11-secocholestan-9-one (**23**), isolated from *Sinularia leptoclados* was found to be cytotoxic against human hepatocellular carcinoma (HepG2), human breast carcinoma (MCF-7 and MDA-MB-231), and human lung carcinoma (A-549) cell lines with IC_50_ values of 8.2, 9.2, 11.3, and 9.8 μg/mL, respectively [92].

Su et al. [93] in another work, also isolated nine cembrane-type diterpenoids, manaarenolides A–I, along with two known cembranolides from *Sinularia manaarensis.* Only manaarenolides G (**185**) and H (**186**) resulted cytotoxic against Hepa 59T/VGH, KB, Hela, and Med cancer cell lines with ED_50_ values ranging from 5.8 to 13.4 μg/mL).

Two new sesquiterpenes, 1*S*,4*R*,5*S*,6*R*,7*S*,10*S*-1(5),6(7)-diepoxy-4-guaiol (**187**) and 1*S*,4*S*,5*S*,10*R*-4,10-guaianediol (**188**) have been isolated from *Sinularia* sp. by Zhang et al. [94]. Both the metabolites showed cytotoxic activity against B-16 (mouse melanoma) and HT-29 (human colon carcinoma) cell lines with IC_50_ ranging values from 1.2 to 5.7 µg mL^−1^.

Ahmed et al. [95] performed another chemical investigation on *S. gibberosa* and they isolated three new polyoxygenated sterols, gibberoketosterols B, C, and gibberoepoxysterol, along with two known steroids, gibberoketosterol and 24-methylenecholest-5-en-3β-ol. Gibberoketosterol (**178**) was found to show cytotoxicity against the proliferation of HepG2 (human liver carcinoma), MCF7, MDA-MB-23 (human breast carcinoma), and A-549 (human lung carcinoma) cell lines with IC_50_ values of 13.0, 14.1, 14.4, and 14.5 μg/mL, respectively. Moreover, gibberoepoxysterol (**189**) showed selective cytotoxicity toward MDA-MB-231 and A-549 cells (IC_50_ 15.9 and 15.5 μg/mL, respectively). 

Ahmed et al. [96] also isolated four new trihydroxysteroids, sinugrandisterols A–D, from *Sinularia grandilobata*. Sinugrandisterol A (**190**) showed cytotoxicity against the proliferation of HepG2 and MDA-MB-231 cells (IC_50_ of 9.1 and 9.2 μg/mL, respectively), while sinugrandisterol B (**191**) was found to be cytotoxic exclusively to HepG2 cancer cell line with IC_50_ of 6.8 μg/mL. 

Two new xeniaphyllane-derived metabolites, gibberosins K (**192**) and L (**193**), extracted from *S. gibberosa* by Chen et al. [97], exhibited activity against A-549 (human lung carcinoma), HepG2 (human hepatocellular carcinoma), and MDA-MB-231 (human breast carcinoma) cell lines with IC_50_ values ranging from 5.5 to 14.5 mg/mL. 

Chen et al. [98] also extracted a novel skeletal norhumulene (sinugibberoside) and six xeniaphyllane-derived compounds (Gibberosins N–S) from *S. gibberosa*. Gibberosin O (**194**) was found to display cytotoxicity toward HepG2 and A-549 cell lines with IC_50_ values of 18.7 and 19.5 mg/mL, respectively, whereas gibberosin Q (**195**) exhibited cytotoxicity exclusively toward A549 cells with IC_50_ value of 11.0 mg/mL.

Ten new cembrane diterpenoids, flexilarins A–J, along with seventeen known compounds were isolated by Lin et al. [99] from *Sinularia flexibilis*. Flexilarin D (**196**) was found to exhibit cytotoxicity against Hep2 (human hepatocarcinoma) cells with ED_50_ at 0.07 μg/mL, comparable to the standard mitomycin C (ED_50_ of 0.06 μg/mL). The compound also showed cytotoxic activity against HeLa (human cervical epithelioid carcinoma), Daoy (hunan medulloblastoma), and MCF-7 (human breast carcinoma) cell lines with ED_50_ of 0.41, 1.24, and 1.24 μg/mL, respectively. Moreover, the cembranolide analogue 11-dehydrosinulariolide (**197**) showed cytotoxic activity toward the above cancer cell lines with ED_50_ values ranging from 1.58 to 3.14 μg/mL.

Liu et al. [100] investigated the cytotoxic effects of 11-dehydrosinulariolode, isolated from *S. leptoclados,* on CAL-27 (oral adenosquamous carcinoma) cells. When a concentration of 1.5 µg/mL of 11-dehydrosinulariolide (**197**) was applied, the results showed that CAL-27 cells viability was reduced to a level of 70% of the control sample. 

Two new cembrane diterpenes, sinulaparvalides A and B, along with the four known related cembranoids, were isolated from S*inularia parva*. The in vitro cytotoxic activity of these natural products was tested on human colon carcinoma (HCT-116), human promyelocytic leukemia (HL-60), and human lung carcinoma (A-549) cell lines. The results showed that only (1*R*,3*S*,4*S*,7*S*,8*R*,11*S*,12*R*)-7-acetoxy-3,4:8,11-diepoxycembr-15(17)-en-16,12-olide (**198**) and sinulaflexiolide K (**199**) were active exclusively against the HCT-116 cancer cell line with IC_50_ values of 14.5 μg/mL and 10.0 μg/mL, respectively [101].

Scabralin A (**200**), a new cadinane-type sesquiterpenoids extracted from *Sinularia scabra* by Su et al. [102] was found to be cytotoxic against human breast (MCF-7), colon (WiDr), medulloblastoma (Daoy), and laryngeal (HEp 2) carcinoma cells with ED_50_ values of 9.6, 10.7, 7.6, and 13.8 µg/mL, respectively.

Seven new diterpenoids, flexibilisolides C–G, flexibilisin C, and 11,12-secoflexibillin along with seven known compounds, were derived from the soft coral *Sinularia flexibilis* [103]. Flexibilisolide C (**201**), 11-dehydrosinulariolide (**197**), 14-deoxycrassin (**44**), 11-episinulariolide acetate (**202**), and 3,4:8,11-bisepoxy-7-acetoxycembra-15(17)-en-1,12-olide (**203**) showed cytotoxicity toward human cervical epithelioid carcinoma (HeLa) with ED_50_ values ranging from 11.6 to 15.6 μg/mL. Moreover, flexibilisolide C (**201**), 11-dehydrosinulariolide (**197**), 14-deoxycrassin (**44**), and 11-episinulariolide acetate (**202**) were found to exhibit cytotoxicity against B-16 (human melanin carcinoma) cell line with ED_50_ values comparable to that of the positive control 5-fluorouracil (9.9 ± 0.8 μg/mL). Moreover, 14-deoxycrassin (**44**) showed activity against the SK-Hepl (human liver carcinoma) cell line with ED_50_ value of 5.7 ± 0.8 μg/mL.

One new sterol, crassarosterol A, and four new steroidal glycosides, crassarosterosides A–D were isolated from *Sinularia crassa* by Chao et al. [104]. Compounds were assayed for cytotoxicity toward human liver carcinoma (HepG2 and HepG3), human breast carcinoma (MCF-7 and MDA-MB-231), and human lung carcinoma (A-549) cells. The results displayed that crassarosterol A (**204**) exhibited cytotoxicity toward the HepG2 cancer cell line with an IC_50_ value of 14.9 µM, while crassarosteroide C showed activity against HepG2 and HepG3 cell lines with IC_50_ values of 17.6 and 18.9 µM, respectively. The other compounds were found to be inactive (IC_50_ > 20 μM) against the tested cancer cells after a 72-h exposure.

Su et al. [105] investigated the cytotoxic activity of sinularin (**205**) (an active compound isolated from *S. flexibilis*) against A2058 melanoma cells. When the melanoma cells were exposed to 3 μg/mL sinularin, cell viability was reduced by 45% compared to the control, so the IC_50_ of sinularin was 3.1 μg/mL. Chromatographic investigation by Lo et al. [106] of *Sinularia flexibilis* afforded six new cembrane diterpenes, sinuladiterpenes A–F, in addition to four known cembranolides (11-episinulariolide acetate, 11-dehydrosinulariolide, 11-episinulariolide, and sinulariolide). Sinuladiterpene B (**206**) was found to exhibit cytotoxic activity against WiDr (human colon adenocarcinoma) cell lines with ED_50_ value of 8.37 mg/mL.

Another chemical investigation on *Sinularia* sp. led to the isolation of two norcembranoidal diterpenes, 5-episinuleptolide acetate and scabrolide D [107]. Cytotoxic activity of the norcembranoidal diterpenes toward K-562 (human erythromyeloblastoid leukemia), MOLT-4 (human acute lymphoblastic leukemia), HTC-116 (human acute promyelocytic leukemia), DLD-1 (human colorectal adenocarcinoma), T-47D (human breast ductal carcinoma), and MDA-MB-231 (human breast adenocarcinoma) cells was studied. The results displayed that 5-episinuleptolide acetate (**207**) exhibited cytotoxicity toward all the tested cells with IC_50_ values ranging from 0.59 to 4.09 μg/mL.

Chemical investigations on the EtOAc-soluble fractions from *Sinularia granosa* led to the isolation of the new 9,11-secosteroid, 8α*H*-3β,11-dihydroxy-5α,6α-expoxy-24-methylene-9,11-secocholestan-9-one (**208**), along with one known steroid 3β,11-dihydroxy-5β,6β-expoxy-24-methylene-9,11-secocholestan-9-one (**209**) [108]. The first metabolite exhibited cytotoxicity (in comparison to the positive control mitomycin-C) against HeLa, HEp2, Daoy, and MCF-7 cancer cell lines with ED_50_ values of 8.21 ± 1.61, 6.21 ± 1.38, 5.53 ± 1.58, and 4.99 ± 0.70 μg/mL, respectively, while the second natural product showed cytotoxicity exclusively against Daoy and MCF-7 cancer cell lines with ED_50_ values of 7.07 ± 0.71 and 9.98 ± 0.32 μg/mL, respectively.

Li et al. [109] extracted from *Sinularia* sp. three new polyoxygenated sterols together with three known ones. Only the known 24-methylenecholestane-3β,5α,6β-triol-6-monoacetate (**210**) was found to show cytotoxicity against the K-562 cell line with an IC_50_ value of 3.18 μM.

Chemical investigations on *Sinularia polydactyla* led to the isolation of two known terpenoids, 24-methylcholestane-3β,5α,6β,25-tetrol 25-monoacetate and 24-methylcholestane-5-en-3β,25-diol, along with a cembranoid diterpene, durumolide C [110]. 24-methylcholestane-3β,5α,6β,25-tetrol 25-monoacetate and durumolide C (**211**) exhibited cytotoxicity against colon (HCT) and epidermoid larynx (Hep2) cancer cells with IC_50_ values ranging from 6.1 and 11.7 μg/mL. Moreover, durumolide C (**211**) was found to have cytotoxic activity against liver cancer cell line (HepG2) with IC_50_ value of 1.0 μg/mL.

Two new 9,11-secosteroids and two known norcembranoids (5-episinuleptolide and sinuleptolide) were isolated from *Sinularia nanolobata* [111]. 22α-acetoxy-24-methylene-3β,6α,11-trihydroxy-9,11-seco-cholest-7-en-9-one (**212**) and 11-acetoxy-24-methylene-1β,3β,6α-trihydroxy-9,11-seco-cholest-7-en-9-one (**213**) displayed cytotoxicity against the P-388 cancer cell line with IC_50_ values of 10.2 and 27.8 μg/mL.

Chemical investigation on *Sinularia flexibilis* led to the isolation of five cembrane-based diterpenoids, including two new metabolites, 11-acetylsinuflexolide and 11-acetyldihydrosinuflexolide. The cytotoxic activity of compounds against four cancer cell lines, including human cervical epithelioid carcinoma (HeLa), laryngeal carcinoma (HEp-2), and breast carcinoma (MCF-7 and MDA-MB-231) were evaluated. The results showed that the known compound sinuflexolide (**161**) was the most potent among the isolated metabolites with IC_50_ values of 8.6, 8.2, 16.0, and 11.3 μg/mL, respectively [112].

A new spatane diterpenoid, leptoclalin A (**214**), extracted from *Sinularia leptoclados* [113] was found to show cytotoxicity toward the proliferation of T-47D (hormone-dependent breast cancer) and K-562 (human chronic myelogenous leukemia) cell lines with IC_50_ values of 15.4 and 12.8 μg/mL, respectively.

Crassarosterol A (**204**), a known steroid isolated from *Sinularia arborea* by Chen et al. [114] exhibited cytotoxicity towards K-562 and MOLT-4 cancer cell lines with IC_50_ of 2.5 and 0.7 μg/mL, respectively.

5-episinuleptolide acetate (**207**), a known norcembranoidal diterpene isolated from *Sinularia* sp., exhibited cytotoxicity against K562, Molt 4 and HL 60 cancer cell lines with IC_50_ values of 4.09, 3.21 and 2.53 μg/mL, respectively [115].

A novel nine-membered macrocyclic polysulfur cembranoid lactone (sinulariaoid A), three new multioxygenated cembranoids (sinulariaoids B, C, and D), together with four known cembranoids (capilloloid, dihydrosinularin, sinularin, and dihydrosinuflexolide) were isolated from *Sinularia* sp. by Lei et al. [116]. Sinulariaoid A (**215**) and sinularin (**205**) were found to inhibit the proliferation of the multidrug-resistant cancer cell lines HepG2/ADM and MCF-7/ADM with IC_50_ values ranging from 9.70 ± 1.77 to 28.88 ± 4.60 μg/mL and they resulted more cytotoxic than the positive control doxorubicin.

**Figure 9:**
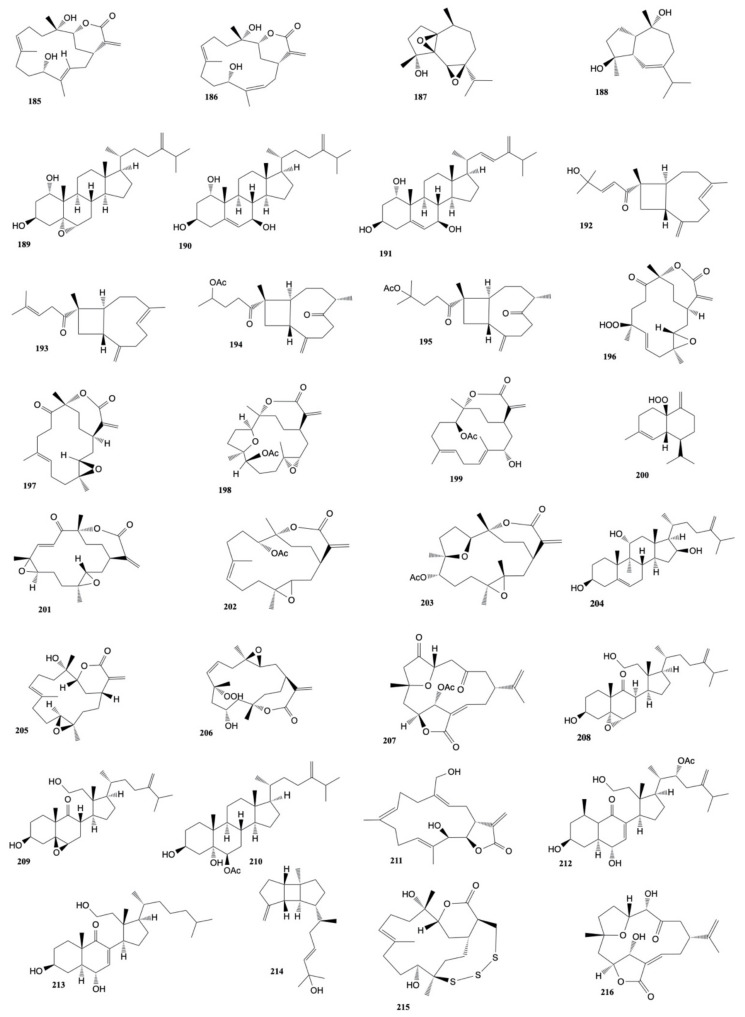
Cytotoxic compounds **185**–**216** derived from the genus *Sinularia*.

Chen et al. [117] isolated a known nor-cembranoidal diterpene, 5-episinuleptolide, along with a new analogue, 4α-hydroxy-5-episinuleptolide, from *Sinularia numerosa*. 4α-hydroxy-5-episinuleptolide (**216**) was found to exhibit cytotoxicity toward the CCRF-CEM (human acute lymphoblastic leukemia) cell line (IC_50_ value of 4.21 μg/mL) and activity against HL-60 (human acute promyelocytic leukemia), K-562 (human chronic myelogenous leukemia), U-937 (human histiocytic lymphoma), and LNCaP (human prostatic carcinoma) cancer cells. The structures of the cytotoxic compounds **185**–**216** derived from the genus *Sinularia* are shown in Figure 9.

Chitturi et al. [118] isolated five new diterpenoids from *Sinularia inelegans*, namely pambanolides A–C (**217**–**219**) and 4,5-secosinulochmodin C (**220**), along with five known compounds. The anti-proliferation effect of the compounds against A-549 (human epithelial lung carcinoma), DU145 (human prostate cancer), HeLa (human epithelial cervical cancer), and MCF-7 (human breast adenocarcinoma) cancer cell lines was evaluated. All these natural products displayed cytotoxic activity toward the four cancer cell lines.

Six steroids, including two new compounds, 3β,4α-dihydroxyergosta-5,24(28)-diene and 24(*S*),28-epoxyergost-5-ene-3β,4α-diol, were isolated from *Sinularia nanolobata*. 24(*S*),28-epoxyergost-5-ene-3β,4α-diol (**221**) showed cytotoxicity against the acute leukemia (HL-60) cell line with IC_50_ value of 33.53 ± 4.25 µM and activity on the hepatoma cancer (HepG2) and colon adenocarcinoma (SW480) cell lines with IC_50_ values of 64.35 ± 7.00 and 71.02 ± 4.00 µM, respectively [119].

Three cadinane-type sesquiterpenoids endoperoxide (**222**) and hydroperoxides (**223, 224**) were isolated from *Sinularia* sp. by Roy et al. [120] and showed cytotoxicity against HCT116.

A methanol extract of *Sinularia microspiculata* revealed five sterols, including two new metabolites, 7-oxogorgosterol and 16α-hydroxysarcosterol [121]. Sarcophytosterol (**225**) showed cytotoxic effects against HL-60 cancer cells (IC_50_ 89.02 ± 9.93 μM), while 3β,7α-dihydroxyergosta-5,24(28)-diene (**226**) was active against HL-60 (IC_50__ _82.80 ± 13.65 μM) and SK-Mel2 (melanoma) (IC_50__ _72.32 ± 1.30 μM).

Four new isoprenoids, including norcembranoids (sinulerectols A and B), a cembranoid (sinulerectol C), and a degraded cembranoid (sinulerectadione), along with three known isoprenoids (an unnamed norcembrene, sinularectin, and ineleganolide) and a known nitrogen-containing compound, were isolated from an extract of *Sinularia erecta* by Huang et al. [122]. The activity of natural products against the growth of promyelocytic leukemia (HL-60), chronic myelogenous leukemia (K-562), T cell lymphoblast-like (CCRF-CEM) and acute lymphoblastic leukemia (MOLT-4) cell lines, were evaluated. The results showed that sinulerectadione (**227**) was cytotoxic toward K-562 and MOLT-4 cancer cell lines with IC_50_ values of 8.6 ± 1.1 and 9.7 ± 2.9 μM, respectively, while the known nitrogen-containing compound (*Z*)-*N*-[2-(4-hydroxyphenyl)ethyl]-3-methyldodec-2-enamide (**228**) exhibited cytotoxicity toward CCRF-CEM and MOLT-4 cells with IC_50_ values of 6.3 ± 1.5 and 9.7 ± 3.6 μM, respectively. Moreover, sinulerectol C (**229**) showed cytotoxicity toward the K-562 cell line with an IC_50_ value of 9.2 ± 3.3 μM. The metabolites displayed comparable and even higher cytotoxicity than the positive control 5-fluorouracil. 

Wu et al. [123] investigated the cytotoxic activity of sinularin (**205**), a known active compound isolated from *S. flexibilis*, against two human gastric cancer cell lines, AGS and NCI-N87. Sinularin at the concentrations of 3, 6, 12, and 18 μM showed a dose-dependent effect on these two cell lines. The effectively reduced cell proliferation of sinularin on AGS cells is 52% (IC_50_ value of 17.73 μM) and on NCI-N87 cells it is 53.5% (IC_50_ value of 15.13 μM).

Fifteen steroids, including two new compounds, leptosteroid and 5,6β-epoxygorgosterol, were derived from *S. leptoclados* by Ngoc et al. [124]. Leptosteroid (**230**) was found to be active against HepG2 (Hepatocellular carcinoma) and SW480 (colon adenocarcinoma) cells with IC_50_ values of 21.13 ± 0.70 and 28.65 ± 1.53 µM, respectively. Moreover, ergost-5-en-3β,7β-diol (**231**) resulted cytotoxic against HL-60 (acute leukemia) and SW48 cell lines with IC_50_ values of 20.53 ± 2.26 µM and 26.61 ± 1.59 µM, respectively. Moreover, 3β,7β-dihydroxyergosta-5,24(28)-diene (**232**) showed cytotoxic activity on all tested cell lines with IC_50_ values ranging from 13.45 ± 1.81 to 29.01 ± 3.21 μM.

*Sinularia brassica* [125] contained five new withanolides (steroidal lactones), sinubrasolides H–L, and the known sinubrasolide A. These metabolites were evaluated for their cytotoxic activity against murine leukemia (P-388), human T-lymphoid (MOLT-4), human erythroleukemia (K-562), and human colon carcinoma (HT-29) cell lines. The results showed that only the metabolites with the hydroxyl group at C-22, sinubrasolide H (**233**), J (**234**), and K (**235**), exhibited activity toward the four cell lines with IC_50_ values ranging from 8.7 ± 1.4 to 39.8 ± 7.7 μM. Moreover, also sinubrasolide A (**236**) was found to display activity against the above cancer cells with IC_50_ of 29.9 ± 3.0 μM, 12.1 ± 1.1 μM, 8.7 ± 1.4 μM, and 18.7 ± 2.5 μM, respectively.

Six ergostane-type steroids, including one new compound (sinubrassione), and two pregnene-type steroid glycosides, including the new sinubrassioside, were isolated from methanol extracts of *Sinularia brassica* [126]. The natural products were tested against three human cancer cell lines: lung carcinoma (A-549), cervical adenocarcinoma (HeLa), and pancreatic epithelioid carcinoma (PANC-1). Ergosta-3β,5α,6β-triol (**237**) exhibited higher cytotoxicity against all three tested cell lines with IC_50_ values of 27.12 ± 1.69, 24.64 ± 1.28, and 20.51 ± 2.72 μM, respectively, compared to that of the positive controls, camptothecin on A-459 (IC_50_ of 12.65 ± 1.01 μM), etoposide on HeLa (IC_50_ of 27.99 ± 2.01 μM), and PANC-1 (IC_50_ of 1.17 ± 0.42 μM). Cytotoxicity was also observed for sinubrassione (**238**) against PANC-1 and A-549 cells with IC_50_ values of 15.24 ± 0.38 and 39.36 ± 0.72 μM. Moreover, ergosta-1α,3β,5α,6β,11α-pentaol (**239**) and pregnedioside A (**240**) showed variable cytotoxic activity toward the three cancer cell lines with IC_50_ values from 16.59 ± 1.77 to 41.76 ± 1.76 μM, while ergosta-1β,3β,5α,6β-tetraol (**241**) displayed activity against PANC-1 with IC_50_ value of 15.39 ± 1.09 μM.

**Figure 10:**
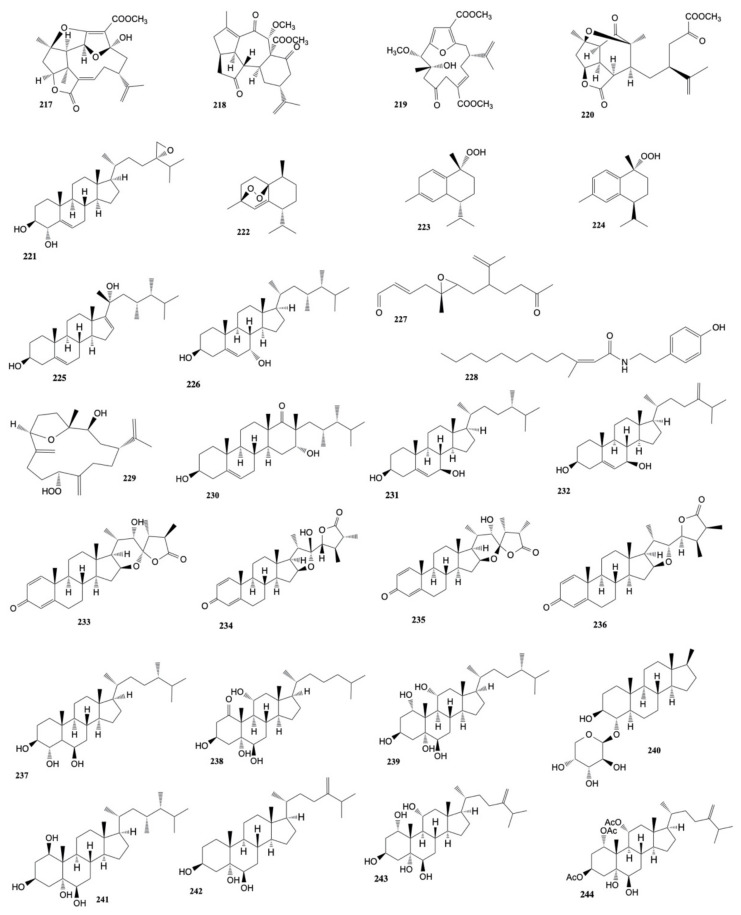
Cytotoxic compounds **217**–**244** derived from the genus *Sinularia*.

Mohammed et al. [127] isolated eight metabolites (five sterols and three sesquiterpenes) from *Sinularia terspilli*. Ergost-24(28)-ene-(3β,5α,6β)-triol, (**242**), ergost-24(28)-ene-(1α,3β,5α,6β,11α)-pentol, (**243**), and ergost-24(28)-ene-(1α,3β,5α,6β,11α)-tetra-acetyl-5-ol, (**244**) with IC_50_ values of 0.004, 0.002, and 0.025 µM, respectively for human acute leukemia HL-60 cells and 0.005, 0.003, and 0.04 µM for the human chronic myelogenous leukemia K-562 cell line compared to taxol, which displayed IC_50_ values of 0.0005 and 0.0023 µM against HL60 and K562, respectively. The other metabolites were less active with IC_50_ values ranging from 0.2 to 0.4 µM. The structures of the cytotoxic compounds **217**–**244** derived from the genus *Sinularia* are shown in Figure 10.

3β,5α-dihydroxyeudesma-4(15),11-diene (**245**), a sesquiterpenoid isolated by Huong et al. [128] from *Sinularia erecta*, exhibited selective cytotoxicity against the A-549 cell line with IC_50_ of 14.79 ± 0.91 μM, relative to that of the positive control, camptothecin, with an IC_50_ value of 11.42 ± 0.13 μM.

A chemical investigation [129] of *Sinularia brassica* cultured in a tank, afforded four new steroids, sinubrasones A–D. The cytotoxicity toward four cancer cell lines, murine macrophage-like (P-388D1), human T-lymphoid (MOLT-4), human erythroleukemia (K-562), and human colon carcinoma (HT-29), were evaluated and sinubrasones B (**247**) and C (**248**) were found to show cytotoxicity against all cell lines (IC_50_ values ranging from 5.2 to 12.1 μM), while sinubrasone A (**246**) and D (**249**) exhibited only cytotoxic activity toward the four cancer cell lines.

A known compound, 5-episinuleptolide (**250**), extracted from *Sinularia compacta* by Wang et al. [130], displayed cytotoxic activity against HCT-116 and A-549 cells, with IC_50_ values of 10.1 and 14.7 μM, respectively.

Sinularin (**205**), a known active compound isolated from *S. flexibilis*, exhibited IC_50_ values of 17.5 ± 6.7 μM and 43.2 ± 8.1 μM against HepG2 and Hep3B, respectively [131].

A novel tetranorditerpenoid (sinubatin A), a new norditerpenoid (sinubatin B), and a known diterpenoid (gibberosin J), were isolated from *Sinularia nanolobata* [132]. Only gibberosin J (**251**) was found to be cytotoxic against P-388, A-549, and HT-29 cancer cell lines with ED_50_ values of 1.0, 1.2, and 0.5 μg/mL, respectively.

A new cembranolide, molestin E (**252**), isolated from *Sinularia* cf. *molesta*, was found to be cytotoxic against HeLa and HCT-116 cell lines with IC_50_ values of 5.26 and 8.37 μM, respectively [133].

Two steroids, ximaosteroid E and ximaosteroid F, along with two known compounds, were extracted from *Sinularia* sp. by Li et al. [134]. Ximaosteroid E (**253**), ximaosteroid F (**254**), and (20*S*)-20-hydroxycholest-1-ene-3,16-dione (**255**) were found to show selective cytotoxicity against the HL-60 cancer cell line with IC_50_ values of 1.79, 4.03, and 0.69 μM, respectively.

Five new cembranoid-related diterpenoids, flexibilisins D and E, secoflexibilisolides A and B, and flexibilisolide H, along with nine known compounds, were isolated from *Sinularia flexibilis* by Wu et al. [135]. The known metabolites 11-dehydrosinulariolide (**197**), 11-episinulariolide acetate (**202**) and 14-deoxycrassin (**44**) were found to inhibit the proliferation of P-388 (murine leukemia), K-562 (human erythromyeloblastoid leukemia), and HT-29 (human colon carcinoma) cell lines with 11-episinulariolide acetate (**202**) exhibiting the most potent cytotoxicity with IC_50_ values ranging from 6.9 μM to 12.2 μM. However, the new compounds were nontoxic toward these cancer cell lines (IC_50_ values > 40 μM).

A sesquiterpenoid isolated by Qin et al. [136] from *Sinularia* sp., sinuketal, showed a cytotoxic toward MDA-MB-231 and U2OS cancer cell lines (IC_50_ values of 32.3 and 41.7 μM, respectively). Moreover, the known cembranoid 5-episinuleptolide (**250**) exhibited cytotoxicity against HeLa and HCT-116 with IC_50_ values of 11.6 and 33.3 μM, respectively.

Two new highly oxygenated ergostane-type sterols (**256, 257**) were isolated from the soft coral *Sinularia* sp. The metabolites exhibited anti-proliferation effect against five human cancer cell lines, including MDA-MB-436, A-549, Hep3B, HT-29, and H157 with IC_50_ values ranging from 10.14 to 41.71 µM [137].

Wu et al. [138] isolated nine compounds from *S. flexibilis*. The compounds were tested against A-549 (lung adenocarcinoma), HT-29 (colonic carcinoma), SNU-398 (hepatocellular carcinoma), and Capan-1 (pancreatic carcinoma) human cancer cell lines. 5-dehydrosinulariolide (**258**) was found to exhibit cytotoxicity against all the above four cell lines with IC_50_ values of 27.4, 22.7, 8.9, and 9.4 µM, respectively, while 11-episinulariolide acetate (**202**) and sinulariolide (**165**) showed cytotoxicity against HT-29, SNU-398, and Capan-1 cells with IC_50_ values ranging from 24.9 to 33.6 µM.

Two novel steroidal derivatives, erectsterates A and B, were isolated from *Sinularia erecta* by Liu et al. [139]. Erecsterates B (**259**) were found to exhibit activity against the proliferation of A-549, HT-29, SNU-398, and Capan-1 cancer cell lines with IC_50_ values of 40.55 ± 7.51, 32.83 ± 14.81, 15.57 ± 5.26, and 23.51 ± 10.46 μM, respectively.

Tammam et al. [140] isolated twenty-six steroids from the organic extract of *Sinularia polydactyla*. Among the tested molecules, 7β-acetoxy-24-methyl-cholesta-5,24(28)-dien-3β,19-diol (**260**) and 7β-acetoxy-cholest-5-en-3β,19-diol (**261**) showed cytotoxic activity against HeLa (IC_50_ values of 7.5 ± 0.1 and 12.0 ± 1.7 μM, respectively) and MCF-7 (IC_50_ values of 8.9 ± 0.0 and 11.2 ± 0.5 μM, respectively) cancer cell lines as compared to the control cisplatin (IC_50_ values of 11.4 ± 3.8 and 7.7 ± 1.7). However, the compounds also showed cytotoxicity toward normal cells (BJ) with IC_50_ values of 14.8 ± 5.8 and 14.5 ± 3.9 μM, respectively.

**Figure 11:**
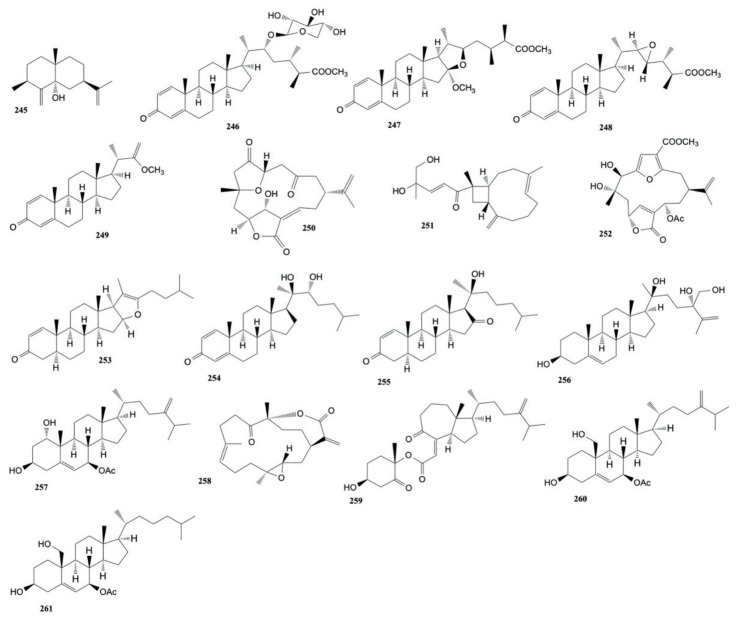
Cytotoxic compounds **245**–**261** derived from the genus *Sinularia*.

A new steroid, 16,17-epoxy-23-methylergostane, isolated from *Sinularia variabilis* by Pour et al. [141], was found to be cytotoxic against MCF-7 and MDA-MB-231 cancer cells with IC_50_ values of 31.44 ± 1.17 and 25.67 ± 1.05 μM, respectively. The structures of the cytotoxic compounds **245**–**261** derived from the genus *Sinularia* are shown in Figure 11.

### 2.4. Cytotoxic Compounds from the Genus Cladiella

The genus *Cladiella* has proven to be a rich source of cytotoxic eunicellin-based diterpenoids. In 2005, Ahmed et al. [142] extracted from *Cladiella australis* four new eunicellin-based diterpenoids, australins A–D. Australin B (**262**) was found to be cytotoxic against MDA-MB-231 (human breast adenocarcinoma), MCF-7 (human breast carcinoma), and HepG2 DMEM-12 (human liver carcinoma) cell lines (ED_50_ values of 6.4, 8.6, and 2.4 µg/mL, respectively), while australin D (**263**) showed only activity toward MDA-MB-231 (ED_50_ 24.4 µg/mL).

Ahmed et al. [143] also isolated (24*S*)-3β-acetoxyergost-5-en-21-oic acid, 5′-*O*-acetylthymidine, 3′-*O*-acetylthymidine, and *p*-vinylbenzyl alcohol, along with a known steroid, (24*S*)-3β-hydroxyergost-5-en-21-oic acid (**264**), from the EtOAc extract of *C. australis*. These compounds were tested against HepG2, Hep3B, MCF-7, and MDA-MB-231 cancer cell lines and the results showed that the known steroid (**264**) was the most potent toward the cancer cells with IC_50_ values ranging from 2.2 to 14.5 µg/mL.

Chen et al. [144] investigated *Cladiella hirsuta* and extracted eight new eunicellin-based diterpenoids, hirsutalins A–H. The cytotoxicity of metabolites against a panel of cancer cell lines including human liver carcinoma (HepG2 and Hep3B), human breast carcinoma (MDA-MB-231 and MCF-7), human lung carcinoma (A-549), and human oral cancer cells (Ca9-22) was investigated. The results showed that hirsutalin A (**265**) had a cytotoxicity toward Hep3B, A-549, and Ca9-22 cell lines with IC_50_ values of 29, 28, and 35 μM, respectively. Moreover, hirsutalin E (**266**) showed to cytotoxicity (IC_50_ values of 14, 41, 35, 34, and 34 μM) toward the proliferation of Hep3B, MDA-MB-231, MCF-7, A-549, and Ca9-22 cells, respectively, and cytotoxicity against the HepG2 (IC_50_ value of 4.7 μM) cell line. Finally, hirsutalin F (**267**) exhibited cytotoxicity toward HepG2, Hep3B, and MCF-7 cell lines with IC_50_ values of 29, 29, and 32 μM, respectively. 

Chen et al. [145] isolated two new eunicellin-type diterpenoids, cladielloides A and B from *Cladiella* sp. Cladielloide B (**268**) showed cytotoxicity toward CCRF-CEM (human T-cell acute lymphoblastic leukemia) and DLD-1 (human colorectal adenocarcinoma) cells with IC_50_ value of 4.7 and 10.2 μM, respectively. The same authors [146] also isolated five new eunicellin-type diterpenoids, cladieunicellins A–E from *Cladiella* sp. Cladieunicellin B (**269**) were found to be cytotoxic toward DLD-1 cancer cells with a IC_50_ value of 2.0 μM and cladieunicellin E (**270**) showed cytotoxicity against the HL-60 leukemia cell line with a IC_50_ value of 2.7 μM. Chen et al. [147] also isolated seven new steroids, hirsutosterols A-G, from *C. hirsuta*. The cytotoxicity of these products against the proliferation of a limited panel of cancer cell lines, including human liver (HepG2 and Hep G3B), breast (MDA-MB-23), and gingival (Ca9-22) carcinoma cells, was evaluated. Hirsusterol A (**271**) was the most potent one toward HepG2, Hep G3B, MDA-MB-23, and Ca9-22 cancer cell lines with IC_50_ values of 16.9, 9.4, 8.2, 18.4, 17.8, and 16.1 μM, respectively. 

Tai et al. [148] isolated four new eunicellin-based diterpenoids, krempfielins A–D, along with two known eunicellin-based diterpenoids, litophynol B and (1*R*,2*R*,3*R*,6*S*,7*S*,9*R*,10*R*,14*R*)-3-butanoyloxycladiell-11(17)-en-6,7-diol from *Cladiella krempfi*. The cytotoxicity of diterpenoids toward lung adenocarcinoma (A-549 and H1299), breast carcinoma (BT-483), liver carcinoma (HepG2), oral cancer (SAS), and human lung bronchial (BEAS-2B) cell lines was evaluated. Only the known metabolites resulted cytotoxic: litophynol B (**272**) showed activity against the proliferation of H1299 and BT-483 cancer cells (ED_50_ values of 18.1 ± 1.5, and 13.2 ± 1.1 μg/mL) and (1R*,2R*,3R*,6S*, 7S*,9R*,10R*,14R*)-3-butanoyloxycladiell-11(17)-en-6,7-diol (**273**) exhibited cytotoxicity toward A549, BT483, and SAS cancer cell lines (ED_50_ values of 15.8 ± 2.0, 8.5 ± 1.0, and 14.3 ± 1.8 μg/mL, respectively). Furthermore, this study also highlighted that these two compounds were non-cytotoxic toward the normal cell BEAS2B.

Tai et al. [149] also isolated five new eunicellin-based diterpenoids from *C. krempfi*, krempfielins E–I, and seven known compounds. Krempfielin I (**274**), litophynol B (**272**), 6-acetoxy litophynin E (**275**), and litophynin F (**276**) displayed cytotoxicity toward A-549, BT-483, H1299, HepG2, and SAS cancer cells with 6-acetoxy litophynin E (**275**) being the most potent one (ED_50_ values ranging from 6.8 ± 1.0 to 11.6 ± 1.0 to 2.8). Unfortunately, except for krempfielin I (**274**), these compounds were also cytotoxic for the human normal cell line BEAS-2B.

Cladieunicellin I (**277**), a new eunicellin diterpenoid isolated from *Cladiella* sp. by Cheng et al. [150], exhibited cytotoxicity against DLD-1 cancer cells with a IC_50_ value of 1.59 μM, being more effective than the positive control doxorubicin (IC_50_ of 10.98 μM).

Cladieunicellin K and cladieunicellin L were extracted from *Cladiella* sp. by Shih et al. [151] and cladieunicellin L (**278**) resulted in cytotoxic towards Molt-4 (human acute T lymphoblastic leukemia) cells with a IC_50_ value of 14.42 μM.

Chen et al. [152] isolated five new 7α-hydroxyeunicellin-based diterpenoids (cladieunicellins M–Q) from *Cladiella* sp. Cladieunicellins M (**279**), O (**280**), and Q (**281**) showed a cytotoxicity toward Molt-4 cancer cells with IC_50_ values of 16.43, 14.17, and 15.55 μM, respectively. Chen et al. [153] also isolated a new 6-hydroperoxyeunicellin diterpenoid, cladieunicellin J (**282**) from *Cladiella* sp. The natural product was cytotoxic toward K-562, Molt-4, CCRF-CEM, and DLD-1 cancer cells with IC_50_ values of 10.9, 6.6, 4.3, 13.4 μg/mL, respectively.

Hirsutalin R (**283**), isolated from *C. hirsuta* by Huang et al. [154], exhibited cytotoxicity toward the proliferation of P-388 (murine leukemia) and K-562 (human erythro myeloblastoid leukemia) cell lines with IC_50_ values of 13.8 and 36.3 μg/mL, respectively, which compared to those of the positive control 5-fluorouracil (8.5 and 24.6 μg/mL, respectively) showed cytotoxicity. Unlike the drug, however, hirsutalin R (**283**) was inactive on HT-29 (human colon adenocarcinoma) and A-549 (human lung adenocarcinoma) cell lines.

Two new pregnane glycosides, hirsutosterosides A (**284**), B (**285**), and two new α-tocopherylhydroquinone glycosides, cladophenol glycosides A (**286**) and B (**287**) were isolated from *C. hirsuta* by Chao et al. [155]. The natural products exhibited cytotoxicity toward four cancer cell lines (K562, P388, HT-29, and A549) with IC_50_ values ranging from 10.2 to 39.3 μM. However, hirsutosteroside A (**284**) was inactive against the growth of P388 and HT-29 cells and hirsutosteroside B (**285**) against the proliferation of A-549 cells.

Cladieunicellin S (**288**), a new eunicellin-type diterpenoid isolated from *Cladiella tuberculosa* by Peng et al. [156], was found to exhibit cytotoxicity against MOLT-4 (human acute lymphoblastic leukemia), K-562 (human chronic myelogenous leukemia), and SUP-T1 (human T-cell lymphoblastic lymphoma) cells with IC_50_ values of 6.04, 6.80, and 6.90 μM, respectively.

**Figure 12:**
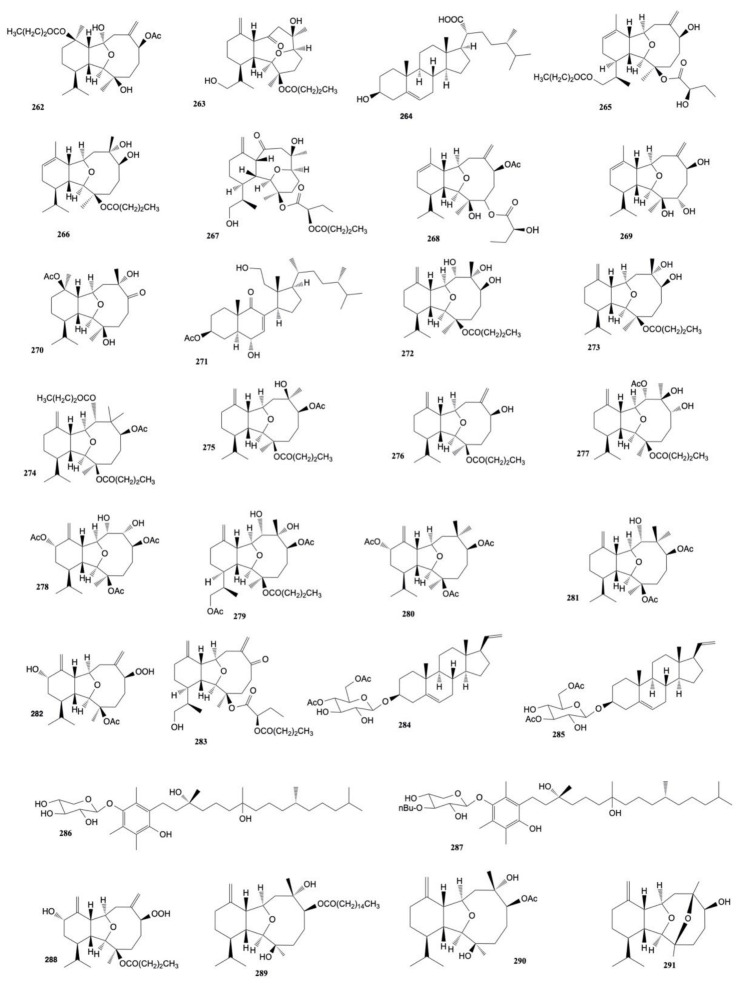
Cytotoxic compounds **262**–**291** derived from the genus *Cladiella*.

Cladieunicellin U, along with a new natural eunicellin, cladieunicellin V, and two known analogues, sclerophytins A and B, were obtained from *Cladiella* sp. by Chen et al. [157]. Cladieunicellin U (**289**) and sclerophytin B (**290**) were found to exhibit cytotoxicity toward the leukemia K-562 cells (IC_50_ of 12.8 and 11.4 μg/mL, respectively). Moreover, cladieunicellin V (**291**) displayed cytotoxicity toward the leukemia MOLT-4 cells (IC_50_ of 18.8 μg/mL). The structures of the cytotoxic compounds **262**–**291** derived from the genus *Cladiella* are shown in Figure 12.

### 2.5. Cytotoxic Compounds from the Genus Klyxum

Wu et al. [158] investigated *Klyxum simplex* and isolated nine eunicellin-based diterpenoids, simplexins A–I, and studied cytotoxicity against human medulloblastoma (Daoy), human breast carcinoma (MCF-7), human cervical epithelioid (HeLa), and human laryngeal (Hep 2) carcinoma cells. The results showed that simplexin E (**294**) exhibited cytotoxicity (in comparison to the positive control doxorubicin) toward the four tested cancer cell lines (IC_50_ values ranging from 7.19 to 17.36 μg/mL) and simplexins A (**292**) and D (**293**) displayed cytotoxicity toward Daoy and MCF-7 cancer cell lines (IC_50_ values ranging from 10.37 to 15.34 μg/mL). Moreover, simplexin A (**292**) displayed cytotoxicity against Hep2 cancer cells with IC_50_ values of 12.10 μg/mL, while simplexins B, C, F, and I were not cytotoxic toward the above cancer cells.

Eight eunicellin-based diterpenoids, klysimplexins A–H, were isolated from the soft coral *K. simplex* by Chen et al. [159]. Klysimplexins B (**295**) and H (**296**) were cytotoxic toward HepG2 and Hep3B (human hepatocellular carcinoma), MDA-MB-231 and MCF-7 (human breast carcinoma), A-549 (human lung epithelial carcinoma), and Ca9-22 (human gingival carcinoma) cell lines with IC_50_ values ranging from 1.8 to 6.9 μg/mL. The results revealed that the α,β-unsaturated ketone in klysimpplexin B (**295**) might be able to enhance the cytotoxicity.

Chen et al. [160] also extracted new eunicellin-base diterpenoids, klysimplexins I–T from *K. simplex.* The results show that klysimplexins Q (**297**) and T (**298**) exhibited cytotoxicity toward HepG2, Hep3B, MDA-MB-231, MCF-7, A-549, and Ca9-22 cell lines with IC_50_ values ranging from 26.4 to 53.2 μM.

Wu et al. [161] isolated four new eunicellin-based diterpenes, simplexins P–S, and the known compound simplexin A, from *K. simplex.* Simplexin R (**300**) showed activity against the proliferation of human chronic myelogenous leukemia (K-562), human T-cell acute lymphoblastic leukemia (CCRF-CEM), human breast carcinoma (T-47D), and human acute lymphoblastic leukemia (MOLT-4) cell lines with ED_50_ values of 7.2 ± 2.4, 2.7 ± 0.1, 13.5 ± 2.8, and 3.8 ± 0.5 µg/mL, respectively. Moreover, simplexins P (**299**), S (**301**), and A (**292**) exhibited to cytotoxicity against CCRF-CEM and MOLT-4 cancer cells with ED_50_ values ranging from 12.0 ± 1.6 to 30.3 ± 3.4 µg/mL.

Lin et al. [162] isolated eleven new eunicellin-based diterpenoids, klymollins I–S, from the EtOAc extract of *Klyxum molle*. Klymollin M (**302**) was found to inhibit the proliferation of K-562, Molt-4, and T-47D cancer cell lines with ED_50_ values 7.97 ± 2.55, 4.35 ± 0.63, and 8.58 ± 1.72 μM, respectively.

Five new eunicellin-based diterpenoids, klymollins T–X, along with two known compounds were isolated from *K. molle* [163]. Among the tested compounds, the known sclerophytin B (**290**) showed activity against the proliferation of CCRF-CEM, K-562, Molt-4, and T-47D cells (ED_50_ values of 4.2, 15.0, 16.5, and 12.4 μg/mL, respectively), and klymollin W (**303**) exhibited cytotoxicity toward CCRF-CEM, Molt 4, and T47D cancer cell lines with ED_50_ values of 9.6, 8.5, and 19.9 μg/mL, respectively.

Six new steroids, klyflaccisteroids A–E and the new 9,11-secogorgosteroid klyflaccisteroid F, along with two known steroids and a known eunicellin-based diterpenoid, were extracted from *Klyxum flaccidum* [164]. Klyflaccisteroid A (**304**) showed selective cytotoxicity toward the growth of A-549 (human lung epithelial carcinoma) cells with ED_50_ 7.7 μg/mL, while klyflaccisteroid E (**307**) showed the strongest cytotoxicity toward the HT-29 (human colon adenocarcinoma) cell line (ED_50_ 6.9 μg/mL). Klyflaccisteroids C (**305**) and E (**307**) displayed the strongest cytotoxic activity against A-549 and P-388 (murin lymphocytic leukemia) cells with ED_50_ values of 6.1 and 3.7 μg/mL, respectively. Also, the known steroid 9,11-secosteroid 3β,11-dihydroxy-9,11-secogorgost-5-en-9-one (**308**) exhibited cytotoxicity against HT29 (IC_50_ of 13.9 μg/mL), A-549 (IC_50_ of 12.5 μg/mL), and P-388 (IC_50_ of 7.1 μg/mL) cell lines, whereas klyflaccisteroid F (**309**) was selectively cytotoxic against A-549 cancer cells with a IC_50_ value of 14.5 μg/mL. Moreover, only klyflaccisteroid C (**305**) e D (**306**) were active against the K-562 (chronic myelogenous leukemia) cell line with ED_50_ of 17.3 and 12.9 μg/mL, respectively.

Two new eunicellin-derived diterpenoid, klymollin Z (**310**) and klyxumollin D (**311**), isolated from *K. molle* exhibited cytotoxicity against the CCRF-CEM cancer cell line, with ED_50_ values of 17.6 and 9.9 μg/mL, respectively [165].

Another chemical investigation on *K. flaccidum* [166] led to the isolation of four new steroids, namely klyflaccisteroids G–J. Klyfaccisteroid J (**313**) was found to be cytotoxic toward the growth of HT-29, P-388, and K-562 cancer cells with IC_50_ values of 15.1, 14.8, and 12.7 μM, respectively. Moreover, Klyfaccisteroid H (**312**) showed selective inhibition toward P-388 cells (IC_50_ value of 15.5 μM).

Ahmed et al. [167] discovered new cembranoids, klyflaccicembranols A–I, along with gibberosene D, from *K. flaccidum.* Klyflaccicembranol B (**314**), F (**316**), and H (**317**) showed cytotoxicity against A-549 human cancer cell lines (IC_50_ of 16.5, 21.4, and 49.4 μM, respectively) in comparison to the positive control Fluorouracil with a IC_50_ value of 110 μM. Klyflaccicembranol B (**314**), D (**315**), and H (**317**) exhibited cytotoxicity toward the growth of K-562 human cancer cells (IC_50_ values of 34.6, 44.9, and 47.4 μM, respectively). Moreover, klyflaccicembranol H (**317**) and I (**318**) were found to be active against P-388 murine cancer line cells with IC_50_ values of 34.6 and 25.9, respectively. 

Chemical investigation [168] on *K. flaccidum* led to the isolation of new steroids, klyflaccisteroids K–M. Klyflaccisteroid K (**319**) possessed to cytotoxicity (in comparison to the positive control Doxorubicin) toward HT-29, K-562, A-549, and DLD-1 cancer cell lines (IC_50_ values of 27.5, 22.5, 15.8, and 27.5 μM, respectively), while klyflaccisteroid M (**320**) exhibited cytotoxicity against HT-29 and P388 cells, with IC_50_ values of 36.7 and 31.8 μM, respectively.

**Figure 13:**
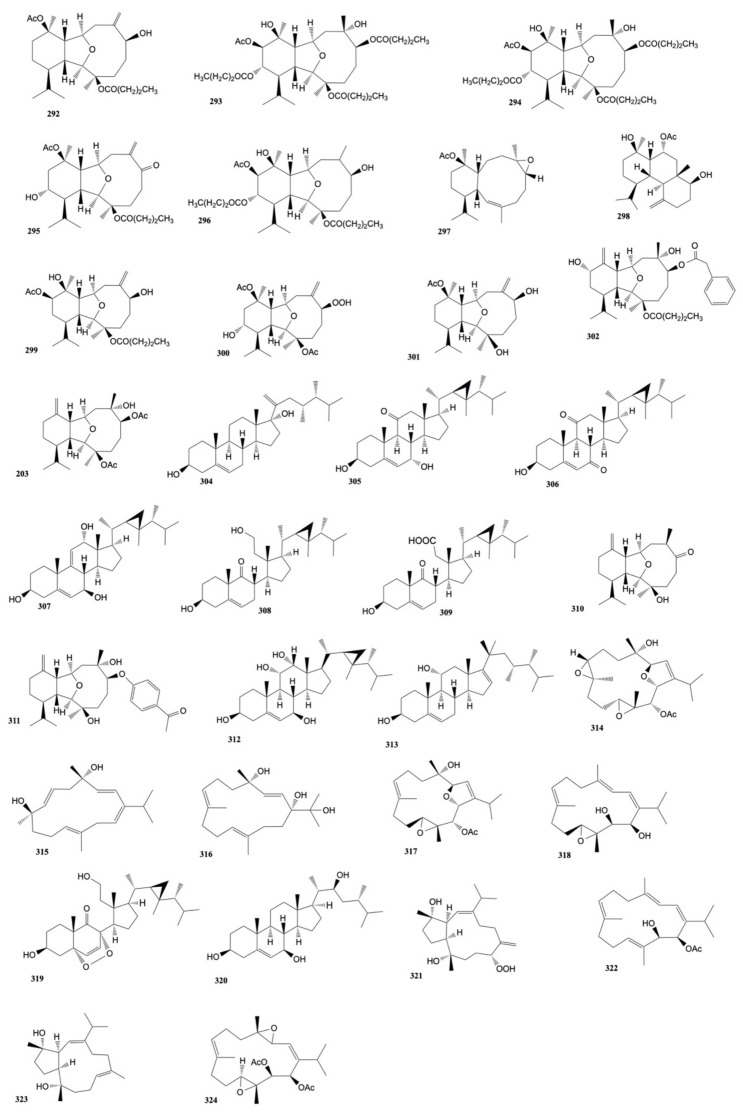
Cytotoxic compounds **292**–**324** derived from the genus *Klyxum*.

Two new capnosane-based diterpenoids, flaccidenol A and 7-epipavidolide D, two new cembranoids, flaccidodioxide and flaccidodiol, and three known compounds were characterized from *K. flaccidum*, by Tseng et al. [169]. Flaccidenol A (**321**) was found to inhibit the growth of human lung adenocarcinoma (A-549), human colorectal adenocarcinoma (DLD-1), and mouse lymphocytic leukemia (P-388D1) cells with IC_50_ values of 9.7 ± 1.2, 6.0 ± 0.4, and 7.2 ± 1.8 µg/mL, respectively. Moreover, 14-*O*-acetylsarcophytol B (**322**) showed cytotoxicity against the above cancer cells (IC_50_ values of 10.8 ± 4.9, 11.7 ± 4.8, and 8.9 ± 2.2 µg/mL, respectively), whereas 7-epipavidolide D (**323**) displayed cytotoxicity toward the tested cells and flaccidodioxide (**324**) was found to be active only against P-388D1 cells. The structures of the cytotoxic compounds **292**–**324** derived from the genus *Klyxum* are shown in Figure 13.

### 2.6. Other Cytotoxic Compounds from Alcyoniidae

A new sterol, 24-methylenecholest-4-ene-3β,6β-diol (**325**) was isolated from the soft coral *Alcyonium patagonicum* and was found to be cytotoxic against the P-388 cell line with an IC_50_ value of 1 µg/mL [170].

Seo et al. [171] extracted seven steroids including five new compounds from *Alcyonium gracillimum*. Hemiketal 2 (**326**) displayed cytotoxicity against the P-388 cancer cell line with a IC_50_ of 7.8 μg/mL.

Xenicane-type diterpenoid, zahavin A (**327**) and B (**328**), isolated from *Alcyonium aureum*, were found to be cytotoxicity against P-388 (murine leukemia), A-549 (human lung carcinoma), MEL-28 (human melanoma), and HT-29 (human colon carcinoma) cell lines with IC_50_ values of 1 µg/mL for almost all cancer cells [172].

Fifteen illudalane sesquiterpenoids, alcyopterosins A–O were isolated from *Alcyonium paessleri* by Palermo et al. [173]. Alcyopterosin E (**331**) showed cytotoxicity toward the Hep-2 (human larynx carcinoma) cell line (IC_50_ value of 13.5 μM), while alcyopterosins A (**329**), C (**330**), and H (**332**) were found to be cytotoxic toward the HT-29 (human colon carcinoma) cell line with a IC_50_ value of 10 μg/mL.

A new diterpene, (Z)-sarcodictyin A (**333**), was isolated from *Bellonella albiflora* [174] and showed cytotoxicity against HeLa human cervix cells.

Seven new steroids were isolated from the Antarctic species *Anthomastus bathyproctus* by Mellado et al. [175]. Methyl (24*E*)-3-oxocholesta-1,4,24-trien-26-oate (**334**) and methyl (22*E*)-3-oxo-24-norcholesta-1,4,22-trien-26-oate (**335**) displayed activity against MDA-MB-231 (breast adenocarcinoma), A-549 (lung carcinoma), and HT-29 (colon adenocarcinoma) cell lines with GI_50_ values ranging from 15.3 to 22.4 μM. Moreover, methyl (22*R*,24*E*)-22-acetoxy-3-oxocholesta-1,4,24-trien-26-oate (**336**) exhibited cytotoxicity toward MDA-MB-231 (GI_50_ of 18.4 μM), while (20*S*)-20-hydroxyergosta-1,4,24(28)-trien-3-one (**337**) against A-549 cells (GI_50_ of 23.4 μM).

Six new withanolides, paraminabeolides A–F, along with five known compounds, minabeolides-1, -2, -4, -5, and -8, were isolated from *Paraminabea acronocephala*. The cytotoxicity of metabolites against HepG2, Hep3B, MDA-MB-231, MCF-7, and A-549 cancer cells was evaluated. The results showed that paraminabeolide A (**338**) and minabeolide-1 (**340**) were selectively cytotoxic toward the HepG2 cancer cell lines with IC_50_ values of 8.0 and 5.2 μM, respectively. Moreover, paraminabeolide B (**339**) was cytotoxic toward MDA-MB-231 and MCF-7 cancer cells with IC_50_ values of 19.3 and 14.9 μM, respectively [176].

Uddin et al. [177] investigated the crude EtOAc extract of *Paraminabea* sp. and reported one new compound (**341**) and five known cholic acid type keto-steroids possessing enone or dienone A-rings and desmosterol. All compounds resulted cytotoxic against NBT-T2 (almost non-invasive rat bladder carcinoma) cells with IC_50_ values of 6.7, 3.1, 17, 5.6, 3.2, 2.1, and 12 μg/mL, respectively.

Three new steroidal carboxylic acids, paraminabic acids A–C, were isolated from *Paraminabea acronocephala*. Paraminabic acid C (**342**) showed cytotoxicity toward Hep3B, MDA-MB-231, MCF-7, and A-549 cancer cell lines, with IC_50_ values ranging from 2.05 to 2.83 μg/mL [178].

**Figure 14:**
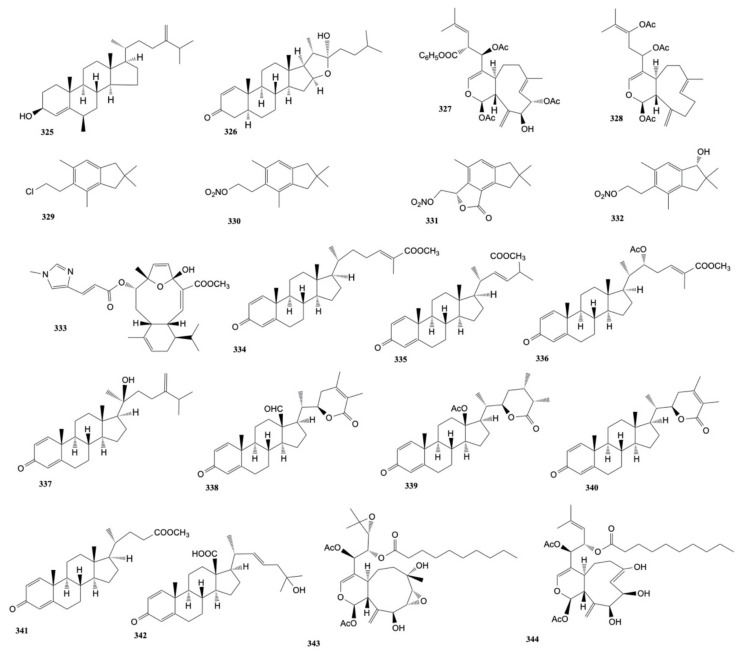
Cytotoxic compounds **325**–**344** derived from other genera of Alcyoniidae.

Two new xenicanes, named protoxenicins A and B, were isolated by Urda et al. [179] from an organic extract of *Protodendron repens*. The cytotoxicity of the natural products against human cancer cell lines NSLC A-549 (lung), HT-29 (colon), and MDA-MB-231 (breast) were evaluated and the result showed that both compounds had a cytotoxic activity: protoxenicin A (**343**) was cytotoxic against the three tested cells (MDA-MB-231, HT-29, and NSLC A-549) with GI_50_ values of 2.1, 0.6, and 1.1 μM, respectively, while protoxenicin B (**344**) exhibited cytotoxic toward HT-29 (GI_50_: 1.7 μM) and cytotoxicity against MDA-MB-231 and NLSC A-549 cells with GI_50_ values of 6.3 and 6.1 μM, respectively. The structures of the cytotoxic compounds **325**–**344** derived from other genera of Alcyoniidae are shown in Figure 14.

## 3. Thirty Years of Compounds Discovery

For a better reading of the data, data reported in the previous section was organized in three tables (Supplementary Materials, Tables S1–S3) compiled by dividing compounds according to their structures: cembranes, cembrane lactones, other terpenes, steroids. The tables report the common chemical names of the selected compounds, the species from which these compounds were isolated, the extraction yields claimed by the authors of the research, the cell lines used for the cytotoxicity test and the results of the assay.

**Figure 15:**
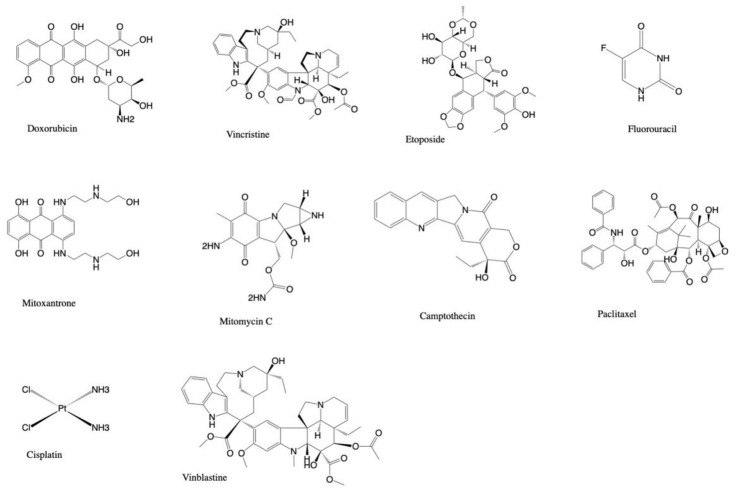
Anticancer drugs used as positive control for the MTT assay.

Considering doxorubicin as positive control, the most active compounds discovered in the last thirty years are 5-episinuleptolide acetate (**207**), sinulariaoid A (**215**), sinularin (**205**), durumolide C (**211**), and cladieunicellin I (**277**), which displayed IC_50_ values of the same order of magnitude of doxorubicin against several cell lines (DLD-1, K-562, HT-29, A-549, HepG2, MCF-7/ADM). In comparison to vincristine, lobophytolin D (**49**) proved to be a very promising compound with a similar cytotoxicity against HT-29 cells and an even higher toxicity against Capan-1 and A-549 cells (IC_50_ values of 6.62 ± 4.02 and 5.17 ± 0.86 μM, respectively; control 6.90 ± 1.81 and 76.06 ± 20.45 μM, respectively). In comparison to etoposide, (24*S*)-ergostane-3β,5α,6β,25 tetraol (**146**) was shown to be the most interesting compound, since it was more cytotoxic than the drug against HepG2 cells. Moreover, acutumosterol A (**147**) and sarcoaldosterol B (**148**) showed IC_50_ values comparable to etoposide toward MCF-7 and A-549 cells. In comparison to 5-fluorouracil, glaucumolides A (**116**) and B (**117**), sinulerectol C (**229**), sinulerectadione (**227**), sinubrasolides H (**233**), J (**234**), K (**235**), and A (**236**) were found to be promising compounds, with a stronger activity than the control against HL-60, CCRF-CEM and K-562 cells. In comparison to mitoxantrone, four compounds were more cytotoxic against several cell lines (A-549 and HT-29 HTC-116, A-549, HL-60), namely (1*S*,2*S*,3*E*,7*E*,11*E*)-3,7,11,15-cembratetraen-17,2-olide (**13**), lobophytosterol (**15**), (22*S*,24*S*)-24-methyl-22,25-epoxyfurost-5-ene-3β,20β-diol (**16**), and 3β,11-dihydroxy-24-methylene-9,11-secocholestan-5-en-9-one (**23**). Noteworthily, the experiments carried out using the anticancer drugs paclitaxel, cisplatin, and vinblastine as positive controls highlighted that no molecules from corals are so far capable to display a comparable cytotoxic activity. This suggests that further tests employing these controls are needed to better verify the cytotoxicity of coral-derived natural products and to identify possible differences in the mechanism of action. The structures of the anticancer drugs used as positive control for the MTT assay in the considered studies are shown in Figure 15.

Considering the isolation yields, the best results were obtained in the extraction of emblide (**70**) with 2790.7 mg per kg of coral (dry weight), 13-acetoxysarcocrassocolide (**42**) with 2033 mg per kg of coral (wet weight), 11-episinulariolide acetate (**202**) with 1757.1 mg per kg of coral (dry weight), and lobohedleolide (**4**) with 1090.9 mg per kg of coral. For the other compounds, the very low yields (more than 1 ton to isolate 1 g of active compound) represent a significant obstacle for the preclinical development, and a synthetic replacement would be required.

Overall, this review clearly shows that considering the chemical structure, the most effective compounds isolated from the Alcyoniidae showed to belong to the group of diterpenoids and steroids. Significant, but scattered, activity was found also from compounds belonging to monoterpenoids, sesquiterpenoids, diterpenoids, triterpenoids, and tetraterpenoids. Most of the described structures are rare and unusual in the natural compounds’ literature and may be specific to the cnidarian world.

The fact that the strongest cytotoxicity was found in compounds belonging to the group of terpenoids is not surprising, since terpenes (and derivatives) represent the largest class of natural products in the biosphere, and their variety of functions in mediating antagonistic and beneficial interactions among organisms is still largely unexplored [180]. Terpenes already include very important chemotherapeutics, such as the taxanes (diterpenes), discovered from the plant world and commonly used in clinical studies, since they are mitotic inhibitors binding tubulin [181].

Among the diterpenoids, cembranoids were the most represented. This name indicates the structure of an isopropyl- and three methyl-substituted 14-membered rings originated by the cyclization of a geranylgeraniol derived precursor between carbons 1 and 14 [182]. Structural changes in the position of double bonds, epoxidation, allylic and isopropyl oxidation, and carbon cyclization are widely observed in these compounds [183] and often correlated with geographic variation and environmental conditions [184], since cembranoids are used by corals as chemical defense to ensure protection against predators, parasites and competing reef organisms [185]. According to a recent review by Nurrachma et al. [186], more than 360 cembranoids are known in the literature. Following Yang et al. [187], cembranoids may be divided in 5 subclasses: (1) simple cembranes, including isopropyl cembranes, isopropenyl cembranes, and isopropyl/isopropenyl acid cembranes subtypes; (2) cembranolides that possess a 14-membered carbocyclic nucleus generally fused to a 5-, 6-, 7-, or 8-membered lactone ring, as observed in *Sarcophyton* with durumolide C (**211**) (5-membered), and in *Sinularia* with manaarelonide A (6-membered) and sinuladiterpene (7-membered); (3) furanocembranoids characterized by a 14-membered carbocyclic nucleus, as well as a furan heterocycle, as observed in *Sinularia* with pukalide; (4) biscembranoids showing a 14-6-14 membered tricyclic backbone of tetraterpenoids, as in lobophytone A found in *Lobophytum pauciflorum.* (5) special cembranes that in turn include several subtypes. Our review clearly shows that among the cembranoids, the best activity at the MTT assay is obtained by molecules owing an odd membered lactone ring (five and seven) such as in the case of deoxycrassin (**44**), flexilarin D (**196**), 11-dehydrosynulariolide (**197**), but also simple cembranes bearing various oxidation display a potent cytotoxic action, such as in sinulerectol C (**229**).

Steroids resulted in the second most represented group in terms of compounds active against cancer cell lines, with a total of 72 compounds. The use of steroids for cancer treatment is clinically well established, and the pharmacology of several active compounds has been described. Steroids mostly work by interacting with specific nuclear hormone receptors that are known to drive cell growth, proliferation, and metastasis for specific tumors (i.e., the estrogen receptor for breast cancer and the androgen receptor for prostate). In contrast, glucocorticoids work through glucocorticoid receptors, which are not considered oncogenes, to perform a variety of functions, including arresting growth or inducing apoptosis in lymphocytes; for these reasons they are widely used in the treatment of all lymphatic cancers [188]. On the other hand, the contribution of the discovery of new steroid structures from the natural word is fundamental to open new clinical development and inspire the work of synthetical chemists.

According to a recent review of Ermolenko et al. [189], about 200 different steroids were discovered from corals, displaying structures belonging to several different types (i.e., secosteroids, spirosteroids, epoxy- and peroxy-steroids, steroid glycosides, halogenated steroids, polyoxygenated steroids, and steroids containing sulfur or nitrogen heteroatoms). In this review, more than 40 steroids were considered potentially capable of displaying cytotoxic activity based on structure-activity relationship predictions (but only a few were tested experimentally). Our review can be considered a completion of this work, since the results of MTT were retrieved and organized for further evaluation: we, in fact, highlighted the presence in nature of at least 33 compounds (from 13 papers) with a very potent cytotoxic action, still not considered in a structure-activity relationship (SAR) study.

A variety of structural variations from the classical steroidal architecture was found in both the tetracyclic ring and the alkyl chain, such as in the case of klyffacisteroid A (**304**) and (22*S*,24*S*)-24-methyl-22,25-epoxyfurost-5-ene-3 (**16**). Interestingly, several active molecules showed the methyl/ethyl substitution typical of phytosterol, as in sarcoaldosterol B (**148**) and acutumosterol A (**147**). Another interesting modification displayed in the coral cytotoxic steroids was the change of the position of the unsaturation in the steroid ring, something commonly observed in algae. On the other hand, the loss of the methyl substituents, which is equally common in the algae, was not observed. Since corals are not capable of providing de novo synthesis of cholesterol and its derivatives, these structures are obtained from phytosterols (provided by intracellular algal symbionts) through de-alkylation and successive oxidation reactions. Signs of the oxidative pathway are clearly observable in various cytotoxic structures, such as the addition of an 11α-hydroxyl group in acutumosterol A (**147**) and sarcoaldosterol B (**148**), a 17α hydroxylation in klyfaccisteroid A (**304**), the oxidation in the lateral chain, even if this does not come with extensive dealkylation, the presence of a conjugated Δ4-3-keto and of an additional unsaturation at Δ1 as in sinubrasolide A (**236**), K (**235**) and in sinubrasones (**247**, **248**).

For some of the more potent cytotoxic compounds listed before, the literature is not limited to in vitro cytotoxicity tests, but studies have gone further by investigating the cellular mechanism of induction of apoptosis and the ability to alter some fundamental properties of cancer cells, such as the capacity to migrate and the potential colony formation were investigated in detail (Suppplementary Materials, Table S4).

Hong et al. [35] showed that (1*S*,2*S*,3*E*,7*E*,11*E*)-3,7,11,15-cembratetraen-17,2-olide (**13**) increased the sub-G1 phase population and induced apoptosis in HT-29 cells through reactive oxygen species generation. Moreover, (1*S*,2*S*,3*E*,7*E*,11*E*)-3,7,11,15-cembratetraen-17,2-olide (**13**) induced apoptosis through the modulation of Wnt/β-catenin and the TGF-β pathways in human colon cancer cells SNU-C5 [36].

Lobophytosterol (**15**) was found to induce apoptosis, in fact chromatin condensation in apoptotic bodies was observed [28].

Sinularin (**205**) induced apoptosis and suppressed the migration capacity of A2058 cancer cells [105]. Wu et al. [123] found that sinularin (**205**) inhibited cell migration capacity and induced apoptosis through mitochondrial dysfunction and inactivation of the pI3K/AKT/mTOR pathway in human gastric carcinoma cell lines. Moreover, sinularin (**205**) activated ATM/Chk2 DNA damage pathway, induced G2/M phase arrest, decreased mitochondial memebrane potential and caused apoptosis in HepG2 (human hepatocellular carcinoma) cell line [131]. Moreover, Sinularin (**205**) induced oxidative stress mediated G2/M arrest and apoptosis in Ca9-22 cancer cells [190] as well as caused G2/M arrest, apoptosis, and oxidative DNA damage in human breast carcinoma (SKBR3) cells [191]. The studies conducted by Ma et al. [192] revealed that sinularin (**205**) induced G2/M arrest and induced apoptosis as well as activation of MAPKs and repression of PI3K/AKT pathways, which are dependent on ROS generation, in human renal cancer (786-O) cells. Recently, Ko et al. [193] have thoroughly investigated the effect of sinularin (**205**) on human hepatocellular cancer (SK-HEP-1) cells and found that wound healing, cell migration, and potential colony formation were attenuated as well as DNA fragmentation and apoptosis were induced and mitochondrial and intracellular reactive oxygen species (ROS) levels were significantly increased following sinularin treatment, which also decreased the mitochondrial membrane potential and caused cytoskeleton disruption [194].

5-Episinuleptolide acetate (**207**) was investigated by Huang et al. [115] and it was found that the compound increased the generation of ROS, the accumulation of intracellular Ca^2+^ as well as decreased the mitochondrial membrane potential and induced apoptosis through the Hsp90 chaperone machinery inhibition in HL-60 (human promyelocytic leukemia) cells.

Sinulariaoid A (**215**) induced apoptosis but its selective toxicity toward HepG2/ADM cells was not related to P-glycoproteins [116].

In addition to these studies, Peng et al. [195] researched the potential synergistic antiproliferation of the combined treatment of ultraviolet-C and sinularin (UVC/sinularin) in oral cancer cells. The results showed that UVC/sinularin synergistically and selectively inhibits cancer cells proliferation with low cytotoxicity on normal oral cells. The cytotoxic mechanism was found to involve apoptosis by increasing cellular and mitochondrial oxidative stress, DNA damage, and mitochondrial dysfunction.

## 4. Challenges and Future Perspective

After several decades of research, marine organisms still deserve to be considered one of the largest untapped resources in the study of natural and chemical diversity. Estimates suggest that from one third to two thirds of eukaryotic marine species still have to be described [196] and the diversity of non-eukaryotic marine microorganisms is also likely far from being characterized [197], suggesting that not only the biodiversity but also the chemical diversity of the marine environments has been only superficially described. A convincing example is the fact that at the present time, less than 5% of the deep-sea has been investigated and an even lower percentage (0.01%) of the deep-sea floor has been sampled and studied [198]. On the other hand, the fast-paced improvement of the explorative technologies is allowing scientists to explore remote and extreme marine environments, such as mesophotic ecosystems and even the deep sea. Indeed, technological advances in remotely operated vehicles, autonomous underwater vehicles, human occupied vehicles, technical SCUBA diving, or even the development of portable hyperbaric chambers, are allowing the discovery and characterization of several previously poorly explored and unknown ecosystems with great potential for biological diversity [199,200,201]. Thus, there is a strong possibility of finding new bioactive natural products in these environments, given the fact that chemical diversity is directly proportional to biological diversity. Soft corals, the taxon considered in this review, with 5800 secondary metabolites already described [202], provide the most astonishing example that support these considerations.

Nowadays, several high throughput technologies are available to study diversity, which can therefore rely on the constantly improved ‘omics’ sciences, such as genomics, transcriptomics, metabolomics, and proteomics [203]. In this sense, the term ‘taxonomics’ has been also proposed to accommodate this highly integrative approach to characterize the Earth’s biodiversity [204]. Specifically, these technologies permit a relatively fast and cost-effective production of huge amounts of data, that can facilitate the description or characterization of species, the deep understanding of their evolution, and the clarification of their relationships with the surrounding environment and organisms. Moreover, these data are useful not only to characterize the diversity of life, but also assist in the discovery of compounds produced by the species and potentially useful for humans [205].

Corals are no exception to this trend, with the knowledge on their diversity, evolution, and ecology being continuously updated, especially in recent years, thanks to the aforementioned ‘omics’ technologies. Indeed, new species are being discovered [206], the diversity of taxonomically problematic taxa is being elucidated [207,208], the evolutionary history of the Cnidaria is being clarified [209,210,211], and highly integrative studies are also revealing that morphologically similar corals ascribable to the same species can be characterized by deep genetic divergence and ecological and physiological differences [212]. As reviewed herein, cnidarians, and in particular octocorals, are promising organisms for the discovery of bioactive compounds [213] and the implementation of the ‘omics’ approach to this group will likely largely enhance the known diversity of the chemical compounds associated with octocorals.

The enormous biological diversity found in the marine environment, as well as the diversity of conditions of sea life, underlie the huge chemical diversity found in marine organisms, exceeding that of terrestrial organisms and representing subspaces in the universe of the chemical diversity different from those available to laboratory synthetic chemistry and terrestrial natural synthesis. Marine compounds are in fact characterized by the high presence of chiral centers and heteroatoms, not only oxygen and nitrogen, but even halogens, as result of their high concentration in seawater and the presence of specialized enzymes [214]. Currently, more than 36,000 compounds of marine origin are already described in the scientific literature [215] and about 1000 more compounds are discovered every year [5]. This is obtained thanks to the fast-pacing improvements in the analytical technology, which provide the access to structure elucidation of unknown compounds even when present at trace concentration levels. Notably, aquaculture techniques applied to corals have experienced significant improvements in the last years, due to aquarium trade, reef restoration, and bioprospecting demand [216]. Accordingly, it has been suggested that coral aquaculture has great potential for metabolite discovery and large-scale production of bioactive compounds [217]. Some examples of bioactive substances extracted from cultivated soft corals, such as *Sinularia*, *Lobophytum,* and *Sarcophyton* species, are already available in literature [218,219,220,221], demonstrating the efficacy of this approach. Therefore, coral aquaculture seems extremely promising for both metabolite discovery, in vitro and in vivo studies, and subsequent production for pharmaceutical use.

In this regard, it is advisable that pharmaceutical industries re-establish natural product-based discovery programs, which may provide new compounds in strategic fields, such as cancer treatments. Drivers for this innovation may be the small biotech companies, which may play important roles in the discovery process if positively supported by academia and government through financed projects. The support of these institutions has a paramount importance since the discovery process is risky, time consuming and needs strong capital investment. An additional challenge is represented by intellectual property protection issues. Also in this case, the involvement of universities and government entities may establish mechanisms that encourage partnerships. Access to the scientific data regarding previous research is equally important.

## 5. Conclusions

The last thirty years of research on cytotoxic compounds from the family Alcyoniidae were reviewed and data were organized to highlight the most promising natural products for further preclinical development. Our work highlighted that 344 distinct secondary metabolites, mainly from the class of cembranoids and steroids, display a cytotoxic activity proven by in vitro assays and deserve attention for further development. Specifically, while for other bioactive compounds mechanistic studies have been undertaken, we found that (22*S*,24*S*)-24-methyl-22,25-epoxyfurost-5-ene-3β,20β-diol (**16**), 3β,11-dihydroxy-24-methylene-9,11-secocholestan-5-en-9-one (**23**), (24*S*)-ergostane-3β,5α,6β,25 tetraol (**146**), sinulerectadione (**227**), sinulerectol C (**229**), and cladieunicellin I (**277**) exhibited stronger cytotoxicity than their respective positive control, but they have not yet been submitted to further investigations devoted to the mechanism of action elucidation.

Moreover, this review clearly showed how corals may be still considered unexploited since the number of published works (164 were retrieved in this review) looks still small when compared to the vastness of biodiversity available and the possibilities offered by coral farming.

## Supplementary Materials

The following supporting information can be downloaded at: https://www.mdpi.com/article/10.3390/md20020134/s1, Table S1: Most active cembranoids; Table S2: Other active terpenes, isolated from Alcyoniidae; Table S3 Most active steroids isolated from Alcyoniidae; Table S4: Futher studies.
