# Supplementary material for: Cytotoxic Compounds from Alcyoniidae: An Overview of the Last 30 Years
Source: Mar Drugs. 2022 Feb 11;20(2):134. doi: 10.3390/md20020134 (PMC8874409; doi:10.3390/md20020134)
Supplement: Supplementary file 1 [file marinedrugs-20-00134-s001.zip › marinedrugs-1547333-supplementary.pdf]

Table S1. Most active cembranoids isolated from Alcyoniidae.

Compound	Species	Cells	Cancer type	Organism	IC_{50} (or ED_{50}) µg/mL or µM	IC_{50} (or ED_{50}) positive control	Quantity of extract per kg of coral	Absolute quantity of isolated products	Ref.
Lobomichaolide (**1**); crassolide (**2**)	*Lobophytum michaelae*	A-549<br>HT-29<br>KB<br>P-388	Lung epithelial carcinoma<br>Colon adenocarcinoma<br>Nasopharyngeal carcinoma<br>Lymphocytic leukemia	Human<br>Human<br>Human<br>Mouse	0.38; 0.39<br>0.37; 0.26<br>0.59; 0.85<br>0.34; 0.08	Not reported	8.9 g/kg	70 mg;<br>50 mg	[22]
Lobohedleolide (**4**)	*Lobophytum crassum*	P-388	Lymphocytic leukemia	Mouse	2.44	Not reported	75.8 g/kg	Not reported	[23]
Lobocrassolide (**3**)	*Lobophytum crassum*	A-549<br>HT-29<br>KB<br>P-388	Lung epithelial carcinoma<br>Colon adenocarcinoma<br>Nasopharyngeal carcinoma<br>Lymphocytic leukemia	Human<br>Human<br>Human<br>Mouse	2.99<br>2.70<br>2.91<br>0.012	Not reported	75.8 g/kg	55 mg	[23]
Lobohedleolide (**4**); Crassumolide A (**5**)	*Lobophytum crassum*	Ca9-22	Gingival squamous cell carcinoma	Human	3.2;<br>2.8	0.1 (Doxorubicin)	20.9 g/kg	1,2 g;<br>9.7 mg	[24]
Lobocrassol (**11**)	*Lobophytum crassum*	P-388	Lymphocytic leukemia	Mouse	3.2	Not reported	30 g/kg	2 mg	[26]
(1S,2S,3E,7E,11E)-3,7,11,15-cembratetraen-17,2-olide (**13**)	*Lobophytum* sp.	A-549<br>HT-29	Lung epithelial carcinoma<br>Colon adenocarcinoma	Human<br>Human	5.1<br>1.8	6.1<br>6.5 (Mitoxantrone)	26.5 g/kg	70 mg	[27]
Drumolide P (**14**)	*Lobophytum durum*	P-388	Lymphocytic leukemia	Mouse	3.8	Not reported	30 g/kg	2 mg	[28]
Lobocrassin B (**18**)	*Lobophytum crassum*	K-562<br>CCRF-CEM<br>Molt-4<br>Hep G2	Chronic myelogenous leukemia<br>T-cell lymphoblastic leukemia<br>Acute lymphoblastic leukemia<br>Hepatocellular carcinoma	Human<br>Human<br>Human<br>Human	2.97<br>0.48<br>0.34<br>3.44	0.24<br>0.05<br>0.07<br>0.71 (Doxorubicin)	Not reported	1.0 mg	[30]
Lobomichaolide (**1**); michaolide L (**29**); michaolide N (**30**); michaolide P (**31**); michaolide Q (**32**)	*Lobophytum michaelae*	P-388<br>HT-29<br>A-549	Lymphocytic leukemia<br>Colon adenocarcinoma<br>Lung epithelial carcinoma	Mouse<br>Human<br>Human	0.3-2.0<br>0.8-4.9<br>1.2-3.2	Not reported	18.2 g/kg	25 mg;<br>5 mg;<br>3 mg;<br>1 mg;<br>1mg	[34]
Lobocrassin B (**18**); 13-acetoxysarcocrassocolide (**42**); 14-deoxycrassin (**44**)	*Lobophytum crassum*	K-562<br>Molt-4<br>U-937<br>Sup-T1	Chronic myelogenous leukemia<br>Acute lymphoblastic leukemia<br>Hystiocytic lymphoma<br>T-lymphoblastic lymphoma	Human<br>Human<br>Human<br>Human	3.3; 3.3; 4.5<br>2.3; 1.2; 2.9<br>5.2; 7.1; 7.0<br>6.2; 1.5; 4.5	0.13<br>0.02<br>0.04<br>0.09 (Doxorubicin)	17.8 g/kg	7.9 mg; 1264.5 mg; 34.3 mg	[40]
Lobophytolin D (**49**)	*Lobophytum* sp.	HT-29<br>Capan-1<br>A-549<br>SNU-398	Colonic carcinoma<br>Pancreatic carcinoma<br>Lung epithelial carcinoma<br>Hepatocellular carcinoma	Human<br>Human<br>Human<br>Human	4.52 ± 0.82<br>6.62 ± 4.02<br>5.17 ± 0.86<br>6.15 ± 2.88	2.39 ± 0.77<br>6.90 ± 1.81<br>76.06 ± 20.45<br>0.36 ± 0.22<br>(Vincristine)	23.8 g/kg (kg of coral in dry weight)	10.5 mg	[42]

Table S1. *Cont.*

Compound	Species	Cells	Cancer type	Organism	IC_{50} (or ED_{50}) μg/mL or μM	IC_{50} (or ED_{50}) positive control	Quantity of extract per kg of coral	Absolute quantity of isolated products	Ref.
Durumolide J (**52**); lobophytolide D (**53**); lobolide A (**54**)	*Lobophytum crassum*	HT-29<br>Capan-1<br>A-549<br>SNU-398	Colonic carcinoma<br>Pancreatic carcinoma<br>Lung epithelial carcinoma<br>Hepatocellular carcinoma	Human<br>Human<br>Human<br>Human	3.1; 2.4; 1.5<br>2.8; 1.9; 1.6<br>7.4; 7.4; 6.9<br>4.0; 2.6; 1.8	0.0024<br>0.0026<br>0.0477<br>0.0008<br>(Vincristine)	7 g/kg	3.7 mg;<br>3.8 mg;<br>28.1 mg	[43]
Sarcocrassolide (**56**); crassolide (**2**); 13-acetoxysarcocrassolide (**57**); denticulatolide (**58**)	*Sarcophyton crassocaule*	A-549<br>HT-29<br>KB<br>P-388	Lung epithelial carcinoma<br>Colon adenocarcinoma<br>Epidermoid carcinoma<br>Lymphocytic leukemia	Human<br>Human<br>Human<br>Mouse	8.31; 4.29; 4.66; 6.46<br>7.55; 4.97; 5.67; 5.78<br>9.15; 8.35; 7.39; 6.29<br>0.16; 0.14; 0.38; 0.15	Not reported	71.4 g/kg	27 mg;<br>54 mg;<br>200 mg;<br>not reported	[45]
Lobophytolide (**53**); Crassocolide A (**66**); crassocolide F (**67**)	*Sarcophyton crassocaule*	HepG2<br>MCF-7<br>MDA-MB-231<br>A-549	Hepatocellular carcinoma<br>Breast carcinoma<br>Breast adenocarcinoma<br>Lung epithelial carcinoma	Human<br>Human<br>Human<br>Human	6.3; 3.1; 2.1<br>2.3; 8.9; 7.4<br>2.0; 8.6; 8.8<br>2.1; 11.9; 3.2	0.2<br>0.3<br>0.1<br>0.2<br>(Doxorubicin)	14.3 g/kg	27.8 mg;<br>47.3 mg;<br>2.2 mg	[49]
Sacrassin A (**71**); sacrassin B (**68**); sacrassin D (**69**); sacrassin E (**72**); emblide (**70**)	*Sarcophyton crassocaule*	KB	Epidermoid carcinoma	Human	19.0; 5.0; 4.0; 13.0; 5.0	Not reported	279 g/kg (kg of coral in dry weight)	30 mg;<br>10 mg;<br>15 mg;<br>6 mg;<br>600 mg	[50]
Sarcostolide E (**75**)	*Sarcophyton stolidotum*	Daoy	Medulloblastoma	Human	5.5	Not reported	10 g/kg	4.5 mg	[51]
Crassocolide H (**80**); crassocolide I (**81**); crassocolide J (**82**); crassocolide K (**83**); crassocolide L (**84**); crassocolide M (**85**)	*Sarcophyton crassocaule*	Daoy	Medulloblastoma	Human	3.8 ± 1.0; 0.8 ± 0.1; 2.8 ± 0.1; 2.5 ± 0.7; 4.1 ± 0.7; 1.1 ± 0.2	0.05 ± 0.02 (Doxorubicin)	14.3 g/kg	1.5 mg;<br>2.1 mg;<br>1.7 mg;<br>5.2 mg;<br>2.6 mg;<br>2.4 mg	[52]
Sarcocrassolide A (**86**); sarcocrassolide B (**87**); sarcocrassolide C (**88**); sarcocrassolide D (**89**)	*Sarcophyton crassocaule*	MCF-7<br>WiDr<br>HEp-2<br>Daoy	Breast carcinoma<br>Colon carcinoma<br>Laryngeal carcinoma<br>Medulloblastoma	Human<br>Human<br>Human<br>Human	4.2; 3.2; 2.0; 4.1<br>4.2; 3.2; 1.2; 1.8<br>6.2; 4.5; 2.6; 4.0<br>8.8; 5.6; 3.2; 5.4	0.14<br>0.15<br>0.07<br>0.14<br>(Mitomycin-C)	14.6 g/kg	10.2 mg;<br>3.0 mg;<br>6.2 mg;<br>5.7 mg	[53]
Sarcocrassolide I (**93**)	*Sarcophyton crassocaule*	MCF-7<br>WiDr<br>HEp-2<br>Daoy	Breast carcinoma<br>Colon carcinoma<br>Laryngeal carcinoma<br>Medulloblastoma	Human<br>Human<br>Human<br>Human	8.4 ± 1.5<br>6.4 ± 2.0<br>5.8 ± 0.5<br>5.1 ± 1.2	0.30 ± 0.12<br>0.47 ± 0.12<br>0.30 ± 0.06<br>0.44 ± 0.06<br>(Mitomycin-C)	14.6 g/kg	4.3 mg	[54]

Table 1. *Cont.*

Compound	Species	Cells	Cancer type	Organism	IC_{50} (or ED_{50}) μg/mL or μM	IC_{50} (or ED_{50}) positive control	Quantity of extract per kg of coral	Absolute quantity of isolated products	Ref.

Crassocolide A (**66**);<br>crassocolide B (**97**);<br>crassocolide E (**40**)	*Sarcophyton crassocaule*	DLD-1<br>CCRF-CEM<br>HL-60	Colonic adenocarcinoma<br>T-cell lymphoblastic leukemia<br>Promyelocytic leukemia	Human<br>Human<br>Human	5.7; 3.8; 7.9<br>6.3; 8.7; 11.1<br>(–); 7.3; 8.4	0.77<br>1.16<br>0.046<br>(Doxorubicin)	14.6 g/kg	3.5 mg;<br>4.3mg;<br>79.8 mg	[55]
7β-Acetoxy-8α-hydroxydeepoxysarcophine (**99**)	*Sarcophyton glaucum*	HepG2<br>HTC-116<br>HeLa	Hepatocellular carcinoma<br>Colon carcinoma<br>Cervical epithelioid carcinoma	Human<br>Human<br>Human	3.6 ± 1.0<br>2.3 ± 1.5<br>6.7 ± 0.8	0.49 ± 0.1<br>0.40 ± 0.1<br>0.60 ± 0.08<br>(Paclitaxel)	5 g/kg	12 mg	[56]
Crassocolide N (**100**);<br>crassocolide O (**101**);<br>crassocolide P (**102**)	*Sarcophyton crassocaule*	KB<br>HeLa<br>Daoy	Epidermoid carcinoma<br>Cervical epitheloid carcinoma<br>Medulloblastoma	Human<br>Human<br>Human	4.7; (–); (–)<br>4.7; (–); 10.8<br>2.8; 4.5; 1,9	0.08<br>0.06<br>0.05<br>(Mitomycin-C)	14.3 g/kg	3.2 mg;<br>2.5 mg;<br>5.2 mg	[57]
Sarcocrassolide M (**103**);<br>sarcocrassolide N (**104**);<br>sarcocrassolide O (**105**)	*Sarcophyton crassocaule*	Daoy	Medulloblastoma	Human	6.6 ± 0.8; 5.2 ± 0.6; 5.0 ± 0.7	0.44 ± 0.06<br>(Mitomycin-C)	14.6 g/kg	4.6 mg;<br>2.2 mg;<br>2.1 mg	[59]
sarcotrocheliol acetate (**109**);<br>deoxosarcophine (**110**);<br>Sarcotrocheliol (**111**)	*Sarcophyton glaucum*	MCF-7	Breast carcinoma	Human	2.4 ± 0.04;<br>9.9 ± 0.03;<br>3.2 ± 0.02;	Not reported	6 g/kg	16 mg;<br>12 mg;<br>30.6 mg;	[61]
7α,8β-dihydroxy-deepoxysarcophine (**115**)	*Sarcophyton auritum*	MCF-7<br>HepG2	Breast carcinoma<br>Hepatocellular carcinoma	Human<br>Human	11.0 ± 0.22<br>18.4 ± 0.16	6.4 ± 0.32<br>4.6 ± 0.11<br>(Cisplatin)	75 g/kg	150 mg	[63]
Glaucumolide A (**116**);<br>glaucumolide B (**117**)	*Sarcophyton glaucum*	HL-60<br>CCRF-CEM	Promyelocytic leukemia<br>T-cell lymphoblastic leukemia	Human<br>Human	6.6 ± 1.2; 3.8 ± 0.9<br>7.4 ± 1.5; 5.3 ± 1.4	10.7 ± 0.5<br>2.30 ± 0.6<br>(Fluorouracil)	7.5 g/kg	4.4;<br>2.8 mg	[64]
Sarcophinone (**118**) and 8-epi-sarcophinone (**119**)	*Sarcophyton glaucom*	HepG2	Hepatocellular carcinoma	Human	11.32 µg/mL (EC50)<br>(tested together)	4.28 µg/mL (EC_{50})<br>(Doxorubicin)	6.64 g/Kg	9.1 mg	[65]
(+)-7α,8β-dihydroxydeepoxysarcophine (**115**);<br>sinumaximol G (**120**);<br>sarcophine (**121**);	*Sarcophyton glaucom*	HepG2	Hepatocellular carcinoma	Human	17.84; 9.97; 10.32 µg/mL (EC50)	4.28 µg/mL (EC_{50})<br>(Doxorubicin)	6.64 g/Kg	52.7 mg; 22.8 mg;<br>530 mg	[65]

Table S1. *Cont.*

Compound	Species	Cells	Cancer type	Organism	IC_{50} (or ED_{50}) µg/mL or µM	IC_{50} (or ED_{50}) positive control	Quantity of extract per kg of coral	Absolute quantity of isolated products	Ref.
(+)-isosarcophytoxide (**135**)	*Sarcophyton mililatensis*	HL-60<br>A-549	Promyelocytic leukemia<br>Lung epithelial carcinoma	Human<br>Human	0.78 ± 0.21<br>1.26 ± 0.80	0.07<br>0.01<br>(Doxorubicin)	29.4 g/kg (dry weight)	51 mg	[69]

7-Acetyl-8-epi-sinumaximol G (**139**);<br>8-epi-Sinumaximol G (**140**);<br>12-Acetyl-7, 12-epi- sinumaximol G (**141**);<br>12-Hydroxysarcoph-10-ene (**142**);<br>8-Hydroxy-epi-sarcophinone (**143**);<br>sinumaximol G (**120**);<br>sarcophine (**121**)	*Sarcophyton* sp.	MCF-7	Breast carcinoma	Human	23.84 ± 0.2; 26.22 ± 0.1; 26.81 ± 0.2; 25.28 ± 0.3;<br>27.2 ± 0.5; 24.97 ± 0.3;<br>22.39 ± 0.2	12.78 ± 0.3<br>(Doxorubicin)	100 g/kg	Not reported	[71]
Sardigitolide B (**144**);<br>glaucumolide A (**116**);<br>glaucumolide B (**117**)	*Sarcophyton digitatum*	MCF-7<br>MDA-MB-231<br>HepG2<br>HeLa	Breast carcinoma<br>Breast carcinoma<br>Hepatocellular carcinoma<br>Cervical epithelioid carcinoma	Human<br>Human<br>Human<br>Human	9.6 ± 3.0; 10.1 ± 3.3; 9.4 ± 3.0<br>14.8 ± 4.0; (–); 17.8 ± 4.5<br>(–); 14.9 ± 3.5; 14.9 ± 4.2<br>(–); 17.1 ± 4.5; (–)	0.7 ± 0.1<br>1.3 ± 0.2<br>1.2 ± 0.4<br>0.4 ± 0.1<br>(Doxorubicin)	1.22 g/kg	4.0 mg;<br>20.2 mg;<br>13.5 mg	[72]
isosarcophytonolide D (**145**)	*Sarcophyton digitatum*	MCF-7	Breast carcinoma	Human	10.9 ± 4.3	0.7 ± 0.1<br>(Doxorubicin)	1.22 g/kg	2.1 mg	[72]
Sarcotenuhydroquinone (**149**);<br>sarcotenusene A (**150**);<br>(2S, 7S, 8S)-sarcophytoxide (**151**);<br>(2S, 7R, 8R)-sarcophytoxide (**152**);<br>sarcophytonin F (**153**);<br>3,4-dihydro-4α-hydroxy-Δ2-sarcophine (**154**);<br>a hydroperoxide (**155**)	*Sarcophyton tenuispiculatum*	MCF-7<br>MDA-MB-231<br>HepG2	Breast carcinoma<br>Breast carcinoma<br>Hepatocellular carcinoma	Human<br>Human<br>Human	25.3 ± 2.8; 34.3 ± 3.7; 37.6 ± 4.2; 33.3 ± 3.5; 30.1 ± 3.1; 24.3 ± 3.0; 27.2 ± 4.0<br>(–); (–); 35.2 ± 4.4; 28.6 ± 3.4; (–); 34.5 ± 4.2; 36.4 ± 5.3<br>36.4 ± 3.6; (–); (–); (–); 38.6±5.0; (–); (–)	6.8 ± 1.4<br>6.3 ± 1.2<br>9.6 ± 1.8<br>(Doxorubicin)	5.1 g/kg	3.5 mg;<br>2 mg;<br>17.5 mg;<br>3 mg;<br>4.5 mg;<br>1.2 mg;<br>3.2 mg	[74]
Sinugibberol (**157**)	*Sinularia gibberosa*	HT-29<br>P-388	Colon adenocarcinoma<br>Lymphocytic leukemia	Human<br>Mouse	0.5<br>11.7	Not reported	75 g/kg	10 mg	[77]
11,12-epoxy-1(E),3(E),7(E)- cembratrien-15-ol (**158**)	*Sinularia gibberosa*	A-549<br>HT-29<br>KB<br>P-388	Lung epithelial carcinoma<br>Colon adenocarcinoma<br>Oral epidermoid carcinoma<br>Lymphocytic leukemia	Human<br>Human<br>Human<br>Mouse	1.03<br>0.64<br>0.63<br>0.01	Not reported	80 g/kg	40mg	[78]

Table S1. *Cont.*

Compound	Species	Cells	Cancer type	Organism	IC_{50} (or ED_{50}) μg/mL or μM	IC_{50} (or ED_{50}) positive control	Quantity of extract per kg of coral	Absolute quantity of isolated products	Ref.
Singardin (**159**)	*Sinularia gardineri*	P-388<br>A-549<br>HT-29<br>MEL-28	Lymphocytic leukemia<br>Lung epithelial carcinoma<br>Colon adenocarcinoa<br>Melanoma	Mouse<br>Human<br>Human<br>Human	1.0<br>2.5<br>5.0<br>5.0	Not reported	4.9 g/kg	19.5 mg	[79]
Sinuflexolide (**161**);<br>sinuflexibilin (**162**)	*Sinularia flexibilis*	P-388<br>A-549	Lymphocytic leukemia<br>Lung epithelial carcinoma	Mouse<br>Human	0.16; 0.27<br>0.68; 0.72	Not reported	150 g/kg	20 mg;<br>8 mg	[80]

		HT-29<br>KB	Colon adenocarcinoa<br>Oral epidermoid carcinoma	Human<br>Human	0.39; 0.22<br>0.46; 1.73				
sarcophytol A (**55**)	*Sinularia* sp.	P-388	Lymphocytic leukemia	Mouse	1.3	Not reported	2.4 g/Kg	Not reported	[81]
Sinulariolide (**165**);<br>flexibilide (**166**);<br>9-Acetoxy-5,8:12,13-diepoxycembr-15(17)-en-16,4-olide (**167**)	*Sinularia capillosa*	P-388<br>L1210	Lymphocytic leukemia<br>Lymphocytic leukemia	Mouse<br>Mouse	8.5; 1.5; 2.5<br>10; 3.0; 5.0	Not reported	Not reported	15 mg;<br>100 mg;<br>20 mg	[82]
Leptocladolide A (**177**);	*Sinularia parva*	KB<br>Hepa59T/VGH	Oral epidermoid carcinoma<br>Liver carcinoma	Human<br>Human	5.9;<br>2.6;	Not reported	4.2 g/kg	0.8 mg;	[86]
Scabrolide E (**179**)	*Sinularia scabra*	KB<br>Hepa59T/VGH	Oral epidermoid carcinoma<br>Liver carcinoma	Human<br>Human	0.7<br>0.5	Not reported	Not reported	8.0 mg	[88]
Capillolide (**168**)	*Sinularia microclavata*	A-549	Lung epithelial carcinoma	Human	0.5	Not reported	14.3 g/kg	30 mg	[90]
Sinularolide B (**8**);<br>sinularolide C (**10**);<br>sinularolide D (**183**);<br>sinularolide E (**184**)	*Sinularia gibberosa*	HL-60<br>BGC-823<br>MDA-MB-435	Promyelocytic leukemia<br>Gastric carcinoma<br>Breast carcinoma	Human<br>Human<br>Human	5.2; 5.1; 2.3; 6.0<br>6.3; 5.2; 6.1; 8.6<br>8.0; 7.7; (–); 2.1	Not reported	22 g/kg	100 mg;<br>48.1 mg;<br>5.0 mg;<br>2.0 mg	[91]
Manaarenolide G (**185**);<br>manaarenolide H (**186**)	*Sinularia manaarensis*	Hepa59T/VGH<br>KB<br>Hela<br>Med	Liver carcinoma<br>Oral epidermoid carcinoma<br>Cervical epithelioid carcinoma<br>Medulloblastoma	Human<br>Human<br>Human<br>Human	7.2; 4.4<br>8.7; 7.6<br>10.9; 9.3<br>13.4; 5.8	Not reported	9.2 g/kg	2.5 mg;<br>2.9 mg	[93]
Flexilarin D (**196**);<br>11-dehydrosinulariolide (**197**)	*Sinularia flexibilis*	HeLa<br>Daoy<br>Hep2<br>MCF-7	Cervical epitheloid carcinoma<br>Medulloblastoma<br>Hepatocarcinoma<br>Breast adenocarcinoma	Human<br>Human<br>Human<br>Human	0.41; 3.04<br>1.24; 2.46<br>0.07; 1.58<br>1.24; 3.14	0.08<br>0.06<br>0.06<br>0.09<br>(Mitomycin-C)	6.9 g/kg	5 mg;<br>34 mg	[99]

Table S1. *Cont.*

Compound	Species	Cells	Cancer type	Organism	IC_{50} (or ED_{50}) μg/mL or μM	IC_{50} (or ED_{50}) positive control	Quantity of extract per kg of coral	Absolute quantity of isolated products	Ref.
Flexibilisolide C (**201**);<br>11-dehydrosinulariolide (**197**);<br>11-epi-sinulariolide acetate (**202**)	*Sinularia flexiblis*	B-16	Melanoma	Human	10.9 ± 0.7;<br>12.5 ± 0.5;<br>12.1 ± 0.8	9.9 ± 0.8<br>(5-Fluorouracil)	38.7 g/ kg (dry weight)	1.2 mg;<br>140 mg; 1230 mg	[103]
14-deoxycrassin (**44**)	*Sinularia flexiblis*	SK-Hepl<br>B-16	Liver carcinoma<br>Melanoma	Human<br>Human	5.7 ± 0.8<br>6.0 ± 0.5	3.4 ± 0.2<br>9.9 ± 0.8<br>(5-Fluorouracil)	31 g/kg (dry weight)	2.1 mg	[103]

5-episinuleptolide acetate (**207**)	*Sinularia* sp.	K-562<br>MOLT-4<br>HTC-116<br>DLD-1<br>T-47<br>MDA-MB-231	Chronic myelogenous leukemia<br>Acute lymphoblastic leukemia<br>Acute promyelocytitc leukemia<br>Colorectal adenocarcinoma<br>Breast ductal carcinoma<br>Breast adenocarcinoma	Human<br>Human<br>Human<br>Human<br>Human<br>Human	0.67<br>0.59<br>4.09<br>0.92<br>3.09<br>2.95	0.15<br>0.01<br>1.11<br>0.22<br>0.40<br>1.30<br>(Doxorubicin)	8.8 g/kg	29.5 mg	[107]
durumolide C (**211**)	*Sinularia polydactyla*	Hep G2<br>HCT<br>Hep2	Hepatocellular carcinoma<br>Colonic carcinoma<br>Epidermoid larynx carcinoma	Human<br>Human<br>Human	1.0<br>11.7<br>10.7	1.2 (Doxorubicin)<br>4.6 (Vinblastine)<br>2.6 (Vinblastine)	Not reported	Not reported	[110]
Sinulariaoid A (**215**);<br>sinularin (**205**)	*Sinularia* sp.	HepG2/ADM<br><br>MCF-7/ADM	Hepatocellular carcinoma multidrug-resistance<br>Breast carcinoma multidrug-resistance	Human<br><br>Human	9.70 ± 1.77; 28.88 ± 4.6<br>16.95 ± 1.82; 23.72 ± 1.38	38.32 ± 6.13<br><br>27.05 ± 2.98<br>(Doxorubicin)	117.4 g/kg<br>(dry weight)	200 mg;<br>8 mg	[116]
4α-hydroxy-5-episinuleptolide (**216**)	*Sinularia numerosa*	CCRF-CEM	T-cell lymphoblastic leukemia	Human	4.21	0.01<br>(Doxorubicin)	10.3 g/kg	2.8 mg	[117]
sinulerectadione (**227**)	*Sinularia erecta*	K-562<br>MOLT-4	Chronic myelogenous leukemia<br>Acute lymphoblastic leukemia	Human<br>Human	8.6 ± 1.1<br>9.7 ± 2.9	33 ± 9<br>6.9 ± 1.5<br>(5-Fluorouracil)	6.4 g/kg	9.0 mg	[122]
Sinulerectol C (**229**)	*Sinularia erecta*	K-562	Chronic myelogenous leukemia	Human	9.2 ± 3.3	33 ± 9<br>(5-Fluorouracil)	6.4 g/kg	1.2 mg	[122]
11-epi-sinulariolide acetate (**202**)	*Sinularia flexibilis*	P-388<br>K-562<br>HT-29	Lymphocytic leukemia<br>Chronic myelogenous leukemia<br>Colonic carcinoma	Mouse<br>Human<br>Human	6.9<br>12.2<br>9.6	0.3<br>1.0<br>0.9<br>(Dox. hydrochloride)	Not reported	84.5 mg	[135]
Klyflaccicembranol B (**314**);<br>klyflaccicembranol D (**215**);<br>klyflaccicembranol F (**316**);<br>klyflaccicembranol H (**317**);<br>klyflaccicembranol I (**318**)	*Klyxum flaccidum*	A-549<br>K-562<br>P-388	Lung epithelial carcinoma<br>Chronic myelogenous leukemia<br>Lymnphocytic leukemia	Human<br>Human<br>Mouse	16.5; (–); 21.4; 49.4; (-)<br>34.6; 44.9; (–); 47.4; (–)<br>(–); (–); (–); 34.6; 25.9	110<br>31.5<br>5.5<br>(5-Fluorouracil)	15 g/kg	1.5 mg;<br>10.2 mg;<br>2.1 mg;<br>3.2 mg;<br>40.5 mg	[167]

Table S2. Other active terpenes, isolated from Alcyoniidae.

Compound	Type	Species	Cells	Cancer type	Organism	IC_{50} (or ED_{50}) μg/mL or μM	IC_{50} (or ED_{50}) positive control	Quantity of extract per kg of coral	Absolute quantity of isolated products	Ref.
Lobophytene (**12**)	Squalene-type triterpene	*Lobophytum* sp.	A-549<br>HT-29	Lung epithelial carcinoma<br>Colon adenocarcinoma	Human<br>Human	8.2; 5.1<br>5.6; 1.8	6.1<br>6.5<br>(Mitoxantrone)	26.5 g/kg	3.5 mg	[27]
Cyclolobatriene (**19**);<br>lobatriene (**20**);<br>eunicol (**21**);<br>fuscol (**22**)	Diterpenes	*Lobophytum pauciflorum*	A-431	Epidermoid carcinoma	Human	0.64; 0.41; 0.35; 0.52	Not reported	4.9 g/kg	7.8 mg;<br>1.2 mg;<br>0.5 mg;<br>1.8 mg	[31]
Methyl tortuate A (**65**);<br>methyl tortuate B (**27**)	Tetracyclic tetraterpenoids	*Sarcophyton tortuosum*	P-388	Lymphocytic leukemia	Mouse	3.5; 5.0	Not reported	Not reported	13 mg;<br>18 mg	[48]
10(14)aromadendrene (**114**)	Sesquiterpene	*Sarcophyton glaucum*	PC-3	Prostate cancer	Human	9.3 ± 0.146	0.50 ± 0.014<br>(Doxorubicin)	6.7 g/kg	15 mg	[62]
prostantherol (**122**)	Sesquiterpene	*Sarcophyton glaucom*	HepG2	Hepatocellular carcinoma	Human	12.22 μg/mL<br>(EC50)	4.28 μg/mL (EC_{50})<br>(Doxorubicin)	6.64 g/Kg	22.2 mg	[65]
guaianediol (**160**)	Sesquiterpene	*Sinularia gardineri*	P-388<br>A-549<br>HT-29<br>MEL-28	Lymphocytic leukemia<br>Lung epithelial carcinoma<br>Colon adenocarcinoa<br>Melanoma	Mouse<br>Human<br>Human<br>Human	1.0<br>2.5<br>5.0<br>5.0	Not reported	4.9 g/kg	16 mg	[79]
1β-hydroxy-α-cyperone (**164**)	Sesquiterpene	*Sinularia* sp.	P-388	Lymphocytic leukemia	Mouse	22.9	Not reported	2.4 g/Kg	Not reported	[81]
Norditerpene 6 (**173**);<br>norditerpene 7 (**174**)	Norditerpenes	*Sinularia scabra*	KB<br>Hepa59/VGH	Oral epidermoid carcinoma<br>Liver carcinoma	Human<br>Human	2.5; 2.3<br>2.6; 2.4	0.3<br>0.3<br>(Doxorubicin)	19.9 g/kg	1.5 mg;<br>5 mg	[85]
Nanolobatin A (**180**);<br>nanolobatinB (**181**)	Norsesquiterpe-noids	*Sinularia nanolobata*	KB<br>Hepa59T/VGH	Oral epidermoid carcinoma<br>Liver carcinoma	Human<br>Human	7.3; 7.6<br>4.6; 8.3	0.25<br>0.47<br>(Doxorubicin)	8 g/kg	5.8 mg;<br>11.4 mg	[89]
1(5),6(7)-diepoxy-4-guaiol (**187**);<br>4,10-guaianediol (**188**)	Sesquiterpenes	*Sinularia* sp.	B-16<br>HT-29	Melanoma<br>Colonic carcinoma	Mouse<br>Human	1.2; 2.2<br>3.5; 4.7	Not reported	117 g/kg (dry weight)	20 mg;<br>6 mg	[94]
Gibberosin K (**192**);<br>gibberosin L (**193**)	Terpenoids	*Sinularia gibberosa*	MDA-MB-231	Breast carcinoma	Human	5.5; 7.7	Not reported	11.8 g/kg	3.8 mg;<br>14.6 mg	[97]

Table S2. *Cont.*

Compound	Type	Species	Cells	Cancer type	Organism	IC_{50} (or ED_{50}) µg/mL or µM	IC_{50} (or ED_{50}) positive control	Quantity of extract per kg of coral	Absolute quantity of isolated products	Ref.
3β,5α-dihydroxyeudesma-4(15),11-diene (**245**)	Sesquiterpenoid	*Sinularia erecta*	A-549	Lung epithelial carcinoma	Human	14.79 ± 0.91	11.42 ± 0.13 (Camptothecin)	100 g/kg	1.5 mg	[128]
Gibberosin J (**251**)	Tetranorditerpe-noid	*Sinularia nanolobata*	P-388<br>A-549<br>HT-29	Lymphocytic leukemia<br>Chronic myelogenous leukemia<br>Colonic carcinoma	Mouse<br>Human<br>Human	1.0<br>1.2<br>0.5	Not reported	10 g/kg	5.0 mg	[132]
Australin B (**262**)	Eunicellin-based diterpenoid	*Cladiella australis*	MDA-MB-231<br>MCF-7<br>HepaG2/DMEM-12	Breast adenocarcinoma<br>Breast carcinoma<br>Liver carcinoma	Human<br>Human<br>Human	6.4<br>8.6<br>2.4	Not reported	14.7 g/kg	3.5 mg	[142]
Hirsutalin E (**266**)	Eunicellin-based diterpenoid	*Cladiella hirsuta*	Hep G2	Hepatocellular carcinoma	Human	4.7	0.4 (Doxorubicin)	10.6 g/kg	31.8 mg	[144]
Cladielloide B (**268**)	Eunicellin-type diterpenoid	*Cladiella* sp.	CCRF-CEM<br>DLD-1	T-cell lymphoblastic leukemia<br>Colorectal adenocarcinoma	Human<br>Human	4.7<br>10.2	0.18<br>0.09<br>(Doxorubicin)	Not reported	2.4 mg	[145]
Cladieunicellin B (**269**)	Eunicellin-type diterpenoid	*Cladiella* sp.	DLD-1	Colorectal adenocarcinoma	Human	2.0	0.09 (Doxorubicin)	Not reported	1.3 mg	[146]
Cladieunicellin E (**270**)	Eunicellin-type diterpenoid	*Cladiella* sp.	HL-60	Promyelocytic leukemia	Human	2.7	0.03 (Doxorubicin)	Not reported	2.6 mg	[146]
6-acetoxy litophynin E (**275**); litophynin F (**276**)	Eunicellin-based diterpenoids	*Cladiella krempfi*	A-549<br>BT-483<br>H1299<br>Hep G2<br>SAS	Lung epithelial carcinoma<br>Breast carcinoma<br>Lung adenocarcinoma<br>Hepatocellular carcinoma<br>Tongue squamous cell carcinoma	Human<br>Human<br>Human<br>Human<br>Human	6.80 ± 1.0; 12.2 ± 1.1<br>11.6 ± 2.8; 6.80 ± 0.6<br>6.70 ± 0.7; 12.8 ± 1.2<br>8.50 ± 1.3; 11.1 ± 0.4<br>9.50 ± 3.7; 10.3 ± 0.5	1.5 ± 0.9<br>3.9 ± 0.8<br>1.2 ± 0.1<br>1.4 ± 0.7<br>2.3 ± 1.5<br>(Taxol)	13.1 g/kg	30.2 mg; 5.4 mg	[149]
Cladieunicellin I (**277**)	Eunicellin diterpenoid	*Cladiella* sp.	DLD-1	Colorectal adenocarcinoma	Human	1.59	10.98 (Doxorubicin)	27.1 g/kg (dry weight)	0.7 mg	[150]

Table S2. *Cont.*

Compound	Type	Species	Cells	Cancer type	Organism	IC_{50} (or ED_{50}) µg/mL or µM	IC_{50} (or ED_{50}) positive control	Quantity of extract per kg of coral	Absolute quantity of isolated products	Ref.
Cladieunicelln J (**282**)	6-hydroxyeuni-cellin-based diterpenoid	*Cladiella* sp.	K-562<br>MOLT-4<br>CCRF-CEM<br>DLD-1	Erythromyeloblastoid leukemia<br>Acute lymphoblastic leukemia<br>T-cell lymphoblastic leukemia<br>Colorectal adenocarcinoma	Human<br>Human<br>Human<br>Human	10.9<br>6.6<br>4.3<br>13.4	Not reported	9.9 g/kg	1.4 mg	[152]
Hirsutalin R (**283**)	Eunicellin-type compound	*Cladiella hirsuta*	P-388<br>K-562	Leukemia<br>Erythron myeloblastoid leuykemia	Mouse<br>Human	13.8<br>36.3	8.50<br>24.6<br>(5-Fluorouracil)	10.6 g/kg	1.4 mg	[154]
Cladieunicellin S (**288**)	Eunicellin-type diterpenoid	*Cladiella tuberculosa*	MOLT-4<br>K-562<br>SUP-T1	Acute lymphoblastic leukemia<br>Erythromyeloblastoid leukemia<br>T-cell lymphoblastic lymphoma	Human<br>Human<br>Human	6.04<br>6.80<br>6.90	0.01<br>0.15<br>0.02<br>(Doxorubicin)	10 g/kg	1.2 mg	[156]
Klysimplexin B (**295**); klysimplexin H (**296**)	Eunicellin-based diterpenoids	*Klyxum simplex*	Hep G2<br>Hep 3B<br>MDA-MB-231<br>MCF-7<br>A-549<br>Ca9-22	Hepatocellular carcinoma<br>Hepatocellular carcinoma<br>Breast carcinoma<br>Breast carcinoma<br>Lung epithelial carcinoma<br>Gingival carcinoma	Human<br>Human<br>Human<br>Human<br>Human<br>Human	3.0; 5.6<br>3.6; 6.9<br>6.9; 4.4<br>3.0; 5.6<br>2.0; 2.8<br>1.8; 6.1	Not reported	10.1 g/kg	1.1 mg;<br>1.0 mg	[159]
Simplexin R (**300**)	Eunicelin-based diterpene	*Klyxum simplex*	K-562<br>CCRF-CEM<br>T-47D<br>MOLT-4	Chronic myelogenous leukemia<br>T-cell lymphoblastic leukemia<br>Breast carcinoma<br>Acute lymphoblastic leukemia	Human<br>Human<br>Human<br>Human	7.20 ± 2.4<br>2.70 ± 0.1<br>13.5 ± 2.8<br>3.80 ± 0.5	2.3 ± 0.2<br>1.8 ± 0.3<br>9.8 ± 1.5<br>2.3 ± 0.3<br>(5-Fluorouracil)	10.9 g/kg	0.9 mg	[161]
Klymollin M (**302**)	Eunicellin-based diiterpenoids	*Klyxum molle*	K-562<br>MOLT-.4<br>T-47D	Chronic myelogenous leukemia<br>Acute lymphoblastic leukemia<br>Breast carcinoma	Human<br>Human<br>Human	7.97 ± 2.55<br>4.35 ± 0.63<br>8.58 ± 1.72	16.22 ± 1.77<br>15.07 ± 1.61<br>50.20 ± 13.22<br>(5-Fluorouracil)	Not reported	3.4 mg	[162]
Flaccidenol A (**321**)	Capnosane-based diterpenoid	*Klyxum flaccidum*	A-549<br>DLD-1<br>P-388D1	Lung epithelial carcinoma<br>Colorectal adenocarcinoma<br>Lymphocytic leukemia	Human<br>Human<br>Mouse	9.7 ± 1.2<br>6.0 ± 0.4<br>7.2 ± 1.8	0.3 ± 0.1<br>1.5 ± 0.2<br>0.9 ± 0.2<br>(Doxorubicin)	15 g/kg	1.5 mg	[169]

Table S3. Most active steroids isolated from Alcyoniidae.

Compound	Type	Species	Cells	Cancer type	Organism	IC_{50} (or ED_{50}) µg/mL or µM	IC_{50} (or ED_{50}) positive control	Quantity of extract per kg of coral	Absolute quantity of isolated products	Ref.

Lobophytosterol (**15**)	Sterol	*Lobophytum laevigatum*	HTC-116<br>A-549<br>HL-60	Colon carcinoma<br>Lung carcinoma<br>Promyelocytic leukemia	Human<br>Human<br>Human	3.2 ± 0.9<br>4.5 ± 0.5<br>5.6 ± 0.4	7.2 ± 0.3<br>7.8 ± 0.4<br>8.2 ± 0.9<br>(Mitoxantrone)	40 g/kg	15 mg	[29]
(22S,24S)-24-methyl-22,25-epoxyfurost-5-ene-3β,20β-diol (**16**);<br>(24S)-24-methylcholest-5-ene-3β,25-diol (**17**)	Sterols	*Lobophytum laevigatum*	HTC-116	Colon carcinoma	Human	6.90 ± 0.8;<br>18.1 ± 1.2	7.2 ± 0.3<br>(Mitoxantrone)	40 g/kg	6 mg;<br>18 mg	[29]
3β,11-dihydroxy-24-methylene-9,11-secocholestan-5-en-9-one (**23**)	Sterol	*Lobophytum compactum*	A-549	Lung epithelial carcinoma	Human	4.97 ± 0.06	7.83 ± 0.04<br>(Mitoxantrone)	7 g/kg	23 mg	[32]
(24S)-24-methylcholestane-3β,5α,6β-triol (**59**);<br>24ξ-methylcholestane-3β,5α,6β,25-tetraol-25-monoacetate (**60**)	Steroids	*Sarcophyton crassocaule*	A-549<br>HT-29<br>KB<br>P-388	Lung epithelial carcinoma<br>Colon adenocarcinoma<br>Epidermoid carcinoma<br>Lymphocytic leukemia	Human<br>Human<br>Human<br>Mouse	6.26; (–)<br>8.35; 4.32<br>5.38; (–)<br>0.14; 3.96	Not reported	71.4 g/kg	Not reported	[45]
23,24-dimethylcholest-16(17)-*E*-ene-3β,5α,6β,20(*S*)-tetraol (**61**)	Polyhydroxy-sterol	*Sarcophyton trocheliopho-rum*	HL-60<br>M-14<br>MCF-7	Promyelocytic leukemia<br>Skin melanoma<br>Breast carcinoma	Human<br>Human<br>Human	2.8<br>4.3<br>4.9	Not reported	12.8 g/kg	6 mg	[46]
(24S)-Ergostane-3β,5α,6β,25 tetraol (**146**)	Polyhydroxy-lated sterol	*Sarchopyton acutum*	HepG2	Hepatocellular carcinoma	Human	17.2 ± 1.5	17.6 ± 0.2<br>(Etoposide)	17.6 g/kg	2.7 mg	[73]
Acutumosterol A (**147**);<br>sarcoaldosterol B (**148**)	Polyhydroxy-lated sterols	*Sarchopyton acutum*	MCF-7<br><br>A-549	Breast carcinoma<br><br>Lung epithelial carcinoma	Human<br><br>Human	28.8 ± 1.9;<br>30.2 ± 4.0<br>27.4 ± 1.2;<br>24.8 ± 2.8	33.6 ± 4.8<br><br>21.5 ± 4.5<br>(Etoposide)	17.6 g/kg	2.2 mg;<br>9 mg	[74]
(22R,23R,24R)-5α,8α-epidioxy-22,23-methylene-24-methylcholest-6-en-3β-ol (**170**);<br>numersterol (**171**);<br>pregnenolone (**172**)	Sterols	*Sinularia* sp.	P-388<br>A-549<br>HT-29<br>KB	Lymphocytic leukemia<br>Lung epithelial carcinoma<br>Colon adenocarcinoa<br>Oral pidermoid carcinoma	Mouse<br>Human<br>Human<br>Human	0.4; 8.3; 7.8<br>2.7; 10.8; 8.6<br>1.4; 1.5; 0.7<br>2.1; 1.9; (–)	Not reported	2.5 g/kg	1.7 mg;<br>18.5 mg;<br>10.2 mg	[84]
Sinugrandisterol A (**190**);<br>sinugrandisterol B (**191**)	Trihydroxy-steroids	*Sinularia grandilobata*	Hep G2	Hepatocellular carcinoma	Human	9.1; 6.8	0.40<br>(Doxorubicin)	33.3 g/kg	12.9 mg;<br>1.8 mg	[96]
24-methylenecholestane-3β,5α,6β-triol-6-monoacetate (**210**)	polyoxyge-nated sterol	*Sinularia* sp.	K-562	Chronic myelogenous leukemia	Human	3.18	Not reported	50 g/kg<br>(dry weight)	27.8 mg	[109]
Crassarosterol A (**204**)	Steroid	*Sinularia arborea*	K-562<br>MOLT-4	Chronic myelogenous leukemia<br>Acute lymphoblastic leukemia	Human<br>Human	2.5<br>0.7	0.3<br>0.001<br>(Doxorubicin)	7.8 g/kg	1.2 mg	[114]

Table S3. *Cont.*

**Compound**	**Species**	**Cells**	**Cancer type**	**Organism**	**IC_{50} (or ED_{50}) μg/mL or μM**	**IC_{50} (or ED_{50}) positive control**	**Quantity of extract per kg of coral**	**Absolute quantity of isolated products**	**Ref.**
Sinubrasolide H (**233**);<br>sinubrasolide J (**234**);<br>sinubrasolide K (**235**);<br>sinubrasolide A (**236**)	*Sinularia brassica*	P-388<br>MOLT-4<br>K-562<br>HT-29	Lymphocytic leukemia<br>Acute lymphoblastic leukemia<br>Chronic myelogenous leukemia<br>Colonic carcinoma	Mouse<br>Human<br>Human<br>Human	(–); 18.7; 18.3; 29.9<br>28.6; 17.2; 13.7; 12.1<br>29.7; 12.6; 17.4; 8.7<br>24.4; 11.2; 20.5; 18.7	5.8 ± 0.6<br>6.2 ± 1.0<br>30.5 ± 6.3<br>7.2 ± 0.7<br>(5-Fluorouracil)	9.3 g/kg	3.0 mg;<br>2.1 mg;<br>0.7 mg;<br>not reported	[125]

Sinubrassione (**238**);<br>ergosta-1α,3β,5α,6β,11α-pentaol (**239**);<br>sarcoaldesterol B (**130**);<br>ergosta-1β,3β,5α,6β-tetraol (**241**);<br>ergosta-3β,5α,6β-triol (**237**); pregnedioside A (**240**)	*Sinularia brassica*	A-549<br><br>Hela<br><br>PANC-1	Lung epithelial carcinoma<br><br>Cervical epitheloid carcinoma<br><br>Pancreatic epithelioid carcinoma	Human<br><br>Human<br><br>Human	23.27–47.46<br><br>(–); 16.59– 81.51<br><br>15.24; 22.47; (–);<br>15.39; 20.51; 38.12	12.65 ± 1.01<br>(Camptothecin)<br>27.99 ± 2.01<br>(Etoposide)<br>1.170 ± 0.42<br>(Etoposide)	34.3 g/kg	1.2 mg;<br>1.6 mg;<br>1.5 mg;<br>1.6 mg;<br>3.7 mg;<br>1.6 mg	[126]
Ergost-24(28)-ene-3, 5, 6-triol, (3β, 5α, 6β)-triol (**242**);<br>ergost-24(28)-ene-1, 3, 5, 6, 11-pentol, (1α, 3β, 5α, 6β, 11α (**243**);<br>ergost-24(28)-ene-1, 3, 6, 11-tetra-acetyl-5-ol, (1α, 3β, 5α, 6β, 11α (**244**)	*Sinularia terspilli*	HL-60<br>K-562	Promyelocytic leukemia<br>Chronic myelogenois leukemia	Human<br>Human	0.004; 0.002; 0.025<br>0.005; 0.003; 0.04	0.0005 ± 0.0008<br>0.0023 ± 0.005<br>(Taxol)	40 g/kg	3.0 mg;<br>3.0 mg;<br>2.0 mg	[127]
Sinubrasone B (**247**);<br>sinubrasone C (**248**)	*Sinularia brassica*	P-388D1<br>MOLT-4<br>K-562<br>HT-29	Lymphoma<br>Acute lymphoblastic leukemia<br>Chronic myelogenous leukemia<br>Colonic carcinoma	Mouse<br>Human<br>Human<br>Human	9.7 ± 1.2; 5.7 ± 1.8<br>6.0 ± 0.4; 5.3 ± 1.3<br>5.2 ± 0.8; 12.1 ± 2.4<br>7.6 ± 2.3; 10.4 ± 2.2	6.2 ± 0.7<br>6.9 ± 1.3<br>33.1 ± 8.9<br>7.7 ± 0.8<br>(5-Fluorouracil)	9.3 g/kg<br>(dry weight)	0.8 mg;<br>1.1 mg	[129]
Ximaosteroid E (**253**);<br>ximaosteroid F (**254**);<br>(20S)-20-hydroxycholest-1-ene-3,16-dione (**255**)	*Sinularia* sp.	HL-60	Promyelocytic leukemia	Human	1.79; 4.03; 0.69	0.03<br>(Doxorubicin)	30.8 g/kg<br>(dry weight)	17.5 mg;<br>8.1 mg;<br>2.1 mg	[134]
7β-acetoxy-24-methyl-cholesta-5,24(28)-dien-3β,19-diol (**260**);<br>7β-acetoxy-cholest-5-en-3β,19-diol (**261**)	*Sinularia polydactyla*	HeLa<br>MCF-7	Cervical epitheloid carcinoma<br>Breast adenocarcinoma	Human<br>Human	7.5 ± 0.1; 12.0 ± 1.7<br>8.9 ± 0.0; 11.2 ± 0.5	11.4 ± 3.8<br>7.7 ± 1.7	154.3 g/kg<br>(dry weight)	2.1 mg;<br>1.0 mg	[140]
(24S)-3β-hydroxyergost-5-en-21-oic acid (**264**);<br>(24S)-3β-acetoxyergost-5-en-21-oic acid	*Cladiella australis*	Hep G2<br>Hep 3B	Hepatocellular carcinoma<br>Hepatocellular carcinoma	Human<br>Human	2.2; 8.6<br>2.8; 3.9	Not reported	14.7 g/kg	10 mg;<br>3.5 mg	[143]
Klyflaccisteroid A (**304**);<br>klyflaccisteroid C (**305**);<br>klyflaccisteroid D (**306**);<br>klyflaccisteroid E (**307**)	*Klyxum flaccidum*	HT-29<br>A-549<br>P-388<br>K-562	Colon adenocarcinoma<br>Lung epithelial carcinoma<br>lymphocytic leukemia<br>Chronic myelogenous leukemia	Human<br>Human<br>Mouse<br>Human	(–); 8.2; 8.0; 6.9<br>7.7; 6.1; 17.5; (–)<br>(–); 10.8; 11.7; 3.7<br>(–); 17.3; 12.9; (–)	1.1<br>14.3<br>0.7<br>4.1<br>(5-Fluorouracil)	15 g/kg	4.3 mg;<br>1.4 mg;<br>1.5 mg;<br>1.1 mg	[164]

Table S3. *Cont.*

**Compound**	**Species**	**Cells**	**Cancer type**	**Organism**	**IC_{50} (or ED_{50}) µg/mL or µM**	**IC_{50} (or ED_{50}) positive control**	**Quantity of extract per kg of coral**	**Absolute quantity of isolated products**	**Ref.**
Klyflaccisteroid F (**309**)	*Klyxum flaccidum*	A-549	Lung epithelial carcinoma	Human	14.5	14.3<br>(5-Fluorouracil)	15 g/kg	4.3 mg	[164]
3β,11-dihydroxy-9,11-secogorgost-5-en-9-one (**308**)	*Klyxum flaccidum*	HT-29<br>A-549<br>P-388	Colon adenocarcinoma<br>Lung epithelial carcinoma<br>lymphocytic leukemia	Human<br>Human<br>Murin	13.9<br>12.5<br>7.1	1.1<br>14.3<br>0.7<br>(5-Fluorouracil)	15 g/kg	200 mg	[164]

24-methylenecholest-4-ene- 3β,6β-diol (**325**)	*Alcyonium Patagonicum*	P-388	Lymphocytic leukemia	Murin	1	Not reported	Not reported	2.3 mg	[170]
Hemiketal 2 (**326**)	*Alcyonium gracillimum*	P-388	Lymphocytic leukemia	Murin	7.8	Not reported	3.1 g/kg	30 mg	[175]
Paraminabeolide A (**338**); Minabeolide-1 (**340**)	*Paraminabea acronocephala*	Hep G2	Hepatocellular carcinoma	Human	8.0; 5.2	0.5 (Doxorubicin)	7.9 g/kg	1.8 mg; 2.0 mg	[180]
Paraminabic acid C (**342**)	*Paraminabea acronocephala*	Hep 3B<br>MCF-7<br>A-549<br>MDA-MB-231	Hepatocellular carcinoma<br>Breast carcinoma<br>Lung epithelial carcinoma<br>Breast adenocarcinoma	Human<br>Human<br>Human<br>Human	2.83<br>2.23<br>2.05<br>2.25	0.40<br>0.68<br>1.33<br>1.32<br>(Doxorubicin)	7.9 g/kg	5.1mg	[178]

Table S4 Further studies.

Compound	Cells	Cancer type	Further studies	Results after treatment	Ref.
5-Episinuleptolide acetate (**207**)	HL-60	Human promyelocytic leukemia	Annexin V-FITC/propidium iodide (PI) double staining apoptotic assay analyzed with flow cytometry	The percentages of annexin-positive cells were increased	[115]
			Evaluation of apoptosis-regulated proteins expression levels by western blotting assay	Caspases -3, -8, and -9 were substantially up-regulated as well as PARP cleavage, H2A.X phosphorylation, and XIAP (a caspase inhibitor) were down-regulated	
			Analysis of the mitochondrial membrane potential (MMP) change by flow cytometric assay with JC-1 cationic dye	The mitochondrial membrane potential was decreased	
			Analysis of reactive oxygen species (ROS) production by flow cytometric assay with a carboxy derivative of fluorescein dye, carboxy-H_2DCFDA	The generation of ROS was increased	
			Analysis of intracellular Ca^{2+} release by flow cytometric assay with a fluorescent calcium indicator, Fluo 3	The accumulation of intracellular Ca^{2+} was increased	
			Evaluation of Hsp90 and its client proteins expression levels by western blotting assay	Hsp90 protein and several client proteins were down-regulated	
Sinulariaoid (**215**)	HepG2/ ADM	Human hepatocellular carcinoma multidrug-resistance	Annexin V-FITC/propidium iodide (PI) double staining apoptotic assay analyzed with flow cytometry	The percentage of late apoptotic cells (Annexin V^+/PI^+) was increased	[112]
			Evaluation of cleaved-PARP, an apoptosis marker, expression levels by western blotting assay	The cleaved form of PARP was up-regulated	
			Evaluation of P-glycoprotein (P-gp) expression levels by western blotting assay	The P-gp expression level was unchanged	
Sinularin (**205**)	A2058	Human melanoma	Wound healing assay and cell migration assay	Cell migration capacity was suppressed	[102]
			Cell cycle distributions determined by propidium iodide (PI) staining and flow cytometry analysis	The population of cells arrested in G2/M checkpoint was increased	
			Annexin V-FITC/propidium iodide (PI) double staining apoptotic assay analyzed with flow cytometry	The percentage of early-stage apoptotic cells (annexin V^+/PI^-) was increased	
			Comparative proteomic analysis validated by western blotting assay	The expression levels of several proteins associated with anti-proliferation, induction of apoptosis as well as oxidative-stress protection were changed	
Sinularin (**205**)	AGS and NCI-N87	Human gastric carcinoma	Transwell cell migration assay	Cell migration capacity was inhibited	[123]
			Analysis of the mitochondrial membrane potential (MMP) change by flow cytometric assay with JC-1 cationic dye	The mitochondrial membrane potential was decreased	
			Evaluation of PI3K/Akt/mTOR pathway by western blotting assay	The expression levels of phosphorylated PI3K, Akt, mTOR, and GSK3β were decreased	
Sinularin (**205**)	HepG2	Human hepatocellular carcinoma	Cell cycle distributions determined by propidium iodide (PI) staining and flow cytometry analysis	The population of cells arrested in G2/M checkpoint was increased	[131]
			Annexin V-FITC/propidium iodide (PI) double staining apoptotic assay analyzed with flow cytometry	The percentages of early apoptotic (annexin V^+/PI^-) and late apoptotic or necrotic cells (annexin V^+/PI^+) were increased	

			Analysis of G2/M-related proteins expression levels by western blotting assay	The expression of G2/M corresponding proteins was changed	
			Analysis of the mitochondrial membrane potential (MMP) change by flow cytometric assay with JC-1 cationic dye	The mitochondrial membrane potential was decreased	
			Evaluation of apoptotic proteins expression levels by western blotting assay	Cleaved caspases 8, 9, 3, PAPR, and Bax were up-regulated as well as Bcl-2 was down-regulated	
			ATM/Chk2 DNA damage pathway analysis by western blotting assay	The expression levels of DNA damage signaling molecules were increased	
Sinularin (**205**)	Ca9-22	Human gingival squamous cell carcinoma	Analysis of ROS flow cytometry patterns	The relative ROS-positive staining of cells were increased	[190]
			Cell morphology analysis of apoptosis	The morphological features of apoptosis, such as apoptotic bodies and cell shrinkage, were visualized	
			Cell cycle distributions determined by propidium iodide (PI) staining and flow cytometry analysis	The population of cells arrested in G2/M checkpoint was increased	
			G2/M arrest signaling proteins analysis by western blotting assay	G2/M regulatory proteins were up-regulated	
			Annexin V-FITC/propidium iodide (PI) double staining apoptotic assay analyzed with flow cytometry	The percentages of annexin V-positive cells (Annexin V^+/PI^+ and Annexin V^+/PI^-) were increased	
			Pancaspase analysis by flow cytometric assay	The percentages of pancaspase-positive cells were enhanced	
Sinularin (**205**)	SKBR3	Human breast carcinoma	Cell cycle analysis by 7-aminoactinomycin D (7AAD) flow cytometric assay	The population of cells arrested in G2/M checkpoint was increased	[191]
			Annexin V/7AAD-based Apoptosis pattern analysis by flow cytometric assay	The percentages of annexin V-positive cells were increased	
			Caspase-based apoptosis patterns analysis by flow cytometric assay	The percentages of pancaspase-positive (Pan (+)) cells were increased	
			ROS generation analysis by DCFH-DA-based flow cytometric assay	The relative ROS-positive staining cells were increased	
			MitoMP change analysis by $DiOC_2(3)$-based flow cytometric assay	The MitoMP-negative (%) cells was increased	
			Analysis of the role of oxidative stress in terms of superoxide detection	The relative MitoSOX-positive (%) cells were increased	
			Evaluation of 8-Oxo-2′-deoxyguanosine (8-oxodG), the main product of oxidative DNA damage, expression	The 8-oxodG staining-positive expression (%) cells was increased	
Sinularin (**205**)	786-O	Human renal carcinoma	Cell cycle distributions determined by propidium iodide (PI) staining and flow cytometry analysis	The population of cells arrested in G2/M checkpoint was increased	[192]
			Evaluation of cell cycle-regulated proteins expression levels by western blotting assay	Cyclin B1, Cdc2 were down-regulated as well as p21 was up-regulated	
			Annexin V-FITC/propidium iodide (PI) double staining apoptotic assay analyzed with flow cytometry	The percentages of Annexin V/PI positive cells were increased	
			Evaluation of several mitochondrial-mediated apoptosis proteins expression levels by western blotting assay	Mitochondrial proteins Smac/DIABLO, Cytochrome c, and pro-apoptotic proteins like Bax and Bad were up-regulated as well as anti-apoptotic proteins like Bcl-2, Mcl-1 and Bcl-xl were down-regulated	

			PI3K/Akt/mTOR pathway analysis by western blotting assay	p-PI3K, p85, p-Akt and p-mTOR were significantly decreased and the levels of PI3K p85, Akt and mTOR were not affected.	
			MAPKs pathway analysis by western blotting assay	The phosphorylated MAPKs (p38 and JNK) were increased	
			Evaluation of intracellular ROS levels using the fluorescent probe DCFH-DA	The relative ROS-positive staining cells were increased	
Sinularin (**205**)	SK-HEP-1	Human hepatocellular carcinoma	Annexin V-FITC/propidium iodide (PI) double staining apoptotic assay analyzed with flow cytometry	The percentages of Annexin V/PI positive cells were increased	[193]
			TUNEL assay used to detect late stages of apoptosis and DNA fragmentation	The percentage TUNEL-positive cells was increased	
			Pro-caspases-3/9 and cleaved-caspases-3/9 expression profiles analysis	The amount of pro-caspases-9 and pro-caspases-3 which was decreased as well as the amount of cleaved-caspase-9 and cleaved-caspase-3 form was increased	
			Measurement of intracellular and mitochondrial ROS levels by flow cytometric assay with CM-H_2DCFDA fluorescent probe and MitoSOX™ Red fluorescent probe, respectively	Both intracellular ROS and mitochondrial O^{2-} levels were elevated	
			Measurement of mitochondrial membrane potential by flow cytometric assay with a fluorescent dyes (Rhodamine 123 or JC-1)	The mitochondrial membrane potential was reduced	
			Mitochondrial functions analysis	The mitochondrial respiration parameters were decreased such as basal mitochondrial respiration, ATP production, maximal respiration, and proton leakage. Also, extracellular acidification rate (ECAR), which is an indicator of cellular glycolysis, was decreased	
			Wound healing measurement (scratch-test assay)	The wound healing potential was significantly decreased	
			Transwell chamber migration assay	The migration ability was decreased	
			Colony formation assay fort the attached cells	The colony formation potential was significantly decreased	
			Soft agar colony formation assay for non-attached cells	The colony formation potential was significantly decreased	
			Microscopically investigation using DAPI and phalloidin fluorescence dyes to track nuclei positions and F-actin filaments, respectively	The distribution of F-actin filaments was significantly altered	
(1*S*,2*S*,3*E*,7*E*,11*E*)-3,7,11,15-cembratetraen-17,2-olide (**13**)	HT-29	Human colon carcinoma	Colony formation assay	The number of colonies was significantly reduced	[35]
			Analysis of reactive oxygen species (ROS) production by flow cytometric assay with a florescent probe, H_2DCFDA	ROS generation was induced	
			Analysis of the mitochondrial membrane potential (MMP) change by flow cytometric assay with Rho-123 dye	The mitochondrial membrane potential was disrupted	
			Measurement of the release of cytochrome c from mitochondria to the cytosol	The release of cytochrome c was increased	
			Cell cycle analysis	The sub-G1 phase population was significantly increased, while the percentages of G0/G1, S and G2/M phase were decreased	
			Evaluation of apoptotic proteins expression levels by western blotting assay	The expression of Bcl-2 and Bid was reduced as well as pro-apoptotic protein Bax, the activation of caspase-3, -8 and -9, and cleavage of PARP was increased	

			Evaluation of the activation of signal transduction by western blotting assay	The phosphorylation of c-Jun N-terminal kinase (JNK) and dephosphorylation of p38, extracellular signal-regulated kinase (ERK), Akt, Src and signal transducer and activator of transcription (STAT)3 were induced	
			Evaluation of antioxidant enzymes expression levels by western blotting assay	The expressions of antioxidant catalase and glutathione peroxidase were abrogated	
(1*S*,2*S*,3*E*,7*E*,11*E*)-3,7,11,15-cembratetraen-17,2-olide (**13**)	SNU-C5/5-FU	Fluorouracil-resistant human colon carcinoma	Cell morphology analysis of apoptosis	The apoptotic bodies were increased	[194]
			Cell cycle distributions determined by propidium iodide (PI) staining and flow cytometry analysis	The sub-G1 phase population was significantly increased, while the percentages of S and G2/M phase were decreased	
			Evaluation of apoptotic proteins expression levels by western blotting assay	Bcl-2 level was decreased as well procaspase-9 cleavage, procaspase-3 cleavage and poly(ADP-ribose) polymerase (PARP) cleavage were increased	
			measurement of the release of cytochrome c from mitochondria to the cytosol	The release of cytochrome c was increased	
			Analysis of the TGF-β signaling pathway by western blotting assay and Co-Immunoprecipitation assay	Smad-3 phosphorylation was increased c-Myc was downregulate. Also, the expression of carcinoembryonic antigen (CEA), a direct inhibitor of TGF-β signaling was decreased. LS-1 decreased the CEA level, as well as the direct interaction between CEA and TGF-βR1 in the apoptosis-induction condition	
(1*S*,2*S*,3*E*,7*E*,11*E*)-3,7,11,15-cembratetraen-17,2-olide (**13**)	SNU-C5	Human colorectal carcinoma	Cell morphology analysis by Hoechst 33342 staining	The sub-G_1 hypodiploid cells and apoptotic bodies were increased	[36]
			Cell cycle distributions determined by propidium iodide (PI) staining and flow cytometry analysis	The sub-G1 phase population significantly increased	
			Evaluation of apoptotic proteins expression levels by western blotting assay	The level of Bax, a pro-apoptotic protein was increased, while the level of Bcl-2, an anti-apoptotic protein, was decreased. In addition, the cleavage of caspase-3 and PARP were increased	
			Analysis of the Wnt/β-catenin signaling pathway by western blotting assay	The levels of phospho-GSK-3β, the inactivated form of GSK-3β, and β-catenin were decreased	
			Analysis of the TGF-β signaling pathway by western blotting assay	Phospho-Smad-3 level was increased as well as the expression of c-Myc, the target protein of TGF-β signaling, was decreased	
Lobophytosterol (**15**)	HCT-116	Human acute promyelocytitc leukemia	Apoptosis assay by staining with DNA-specific fluorescent dye	Chromatin condensation in apoptotic bodies was observed	[28]
